# Supplementary material for: Comparative performance of ReMELD-Na, MELD 3.0 and established scores after TIPS for refractory ascites: A multicenter study
Source: JHEP Rep. 2026 Feb 21;8(5):101795. doi: 10.1016/j.jhepr.2026.101795 (PMC13091292; doi:10.1016/j.jhepr.2026.101795)
Supplement: Multimedia component 1 [file mmc1.pdf]

Comparative performance of ReMELD-Na, MELD 3.0 and established scores after TIPS for refractory ascites: A multicenter study

Markus Kimmann, Nancy Farouk, Dominik Bettinger, Johannes Chang, Roman Kloeckner, Cristina Ripoll, Felix Piecha, Jassin Rashidi-Alavijeh, Juliana Stadtmann, Ahmad Shikh Mousa, Tony Bruns, Cornelius Engelmann, Benjamin Maasoumy, Christian Labenz, Lukas Sturm, Hauke Heinzow, Leon Louis Seifert, Michael Köhler, Max Masthoff, Johannes Kluwe, Alexander Zipprich, Christian Jansen, Carsten Meyer, Michael Schultheiss, Jonel Trebicka, Michael Praktiknjo, German Cirrhosis Study Group

Table of contents

Supplementary text 1…………………………………………………………………2

Supplementary text 2…………………………………………………………………3

Supplementary figures………………………………………………………...…..4

Supplementary tables………………………………………………………………….27

Supplementary references………………………………………………………....36

Supplementary text 1

Calculation of the scores:

MELD = (0.957*ln(creatinine) + 0.378*ln(bilirubin) + 1.120*ln(INR) + 0.643)*10 (dialysis treatment twice a week sets a creatinine value of 4 mg/dl, lower capping for every value of 1, upper capping of 4 mg/dl for creatinine).

MELD-Na = MELD + 1,32*(137-sodium) – (0.033*MELD*(137-sodium) (lower and upper capping of 125-137 mmol/L for sodium).

MELD 3.0 = 1.33 (if female) + 4.56*ln(bilirubin) + 0.82*(137-Na) – 0.24*(137 Na)*ln(bilirubin) + 9.09*ln(INR) + 11.14*ln(creatinine) + 1.85*(3.5 – albumin) – 1.83*(3.5 – albumin)*ln(creatinine) + 6 (lower and upper capping of 125-137 mmol/L for sodium and 1.5-3.5 g/dl for albumin, upper capping of 3 mg/dl for creatinine).

FIPS = 1.43*log10(bilirubin) – 1.71*1/creatinine + 0.02*age – 0.02*albumin + 0.81

ReMELD-Na = 7,85 + 9,03*ln(creatinine) + 2,97*ln(bilirubin) + 9,52*ln(INR) + 0,392*(138,6-sodium) – 0,351*(138,6-sodium)*ln(creatinine) (lower and upper capping of 0.7-0.25 mg/dl for creatinine, 0.3-27 mg/dl for bilirubin, 1.0-2.6 for INR and 120-138.6 mmol/L for sodium).

Supplementary text 2

Description of the spline analyses using R:

The spline analyses were purely descriptive visualizations and were not embedded in a logistic or Cox regression model. While the study outcomes (90-day and one-year combined event (Death/LTx)-free survival) are intrinsically time-to-event outcomes, the spline figures did not use any form of survival modelling. For plotting purposes, the outcome was used only as a binary grouping variable (event vs. event-free), but no logistic regression was fitted. Instead, for each pairwise score comparison, we visualized the relationship between the x-axis score (ReMELD-Na or MELD 3.0) and the comparator score using separate smooth curves for event and non-event patients.

These curves were generated in ggplot2 (R) using:

geom_smooth(method = "lm", formula = y ~ ns(x, df = 3)),

which fits a simple linear model with a natural cubic spline transformation of the x-variable. No risk modelling was applied in these plots.

All spline curves used natural cubic splines (ns() function, splines package in R) with 3 degrees of freedom. For natural cubic splines, the number of interior knots is always equal to df − 1; therefore, df = 3 corresponds to two interior knots, with the remaining flexibility defined by the natural boundary constraints. This specification was applied identically across all comparisons, both for ReMELD-Na and for MELD 3.0, and separate spline fits were generated for the event and non-event groups. The knot locations were not set manually. When using ns() with df = 3, R automatically places the two interior knots at the empirical 33rd and 67th percentiles of the respective x-axis variable (ReMELD-Na or MELD 3.0) within each outcome subgroup, and the boundary knots at the minimum and maximum values. This is the default behaviour of ns() and was used throughout the analysis.

Supplementary figures

1

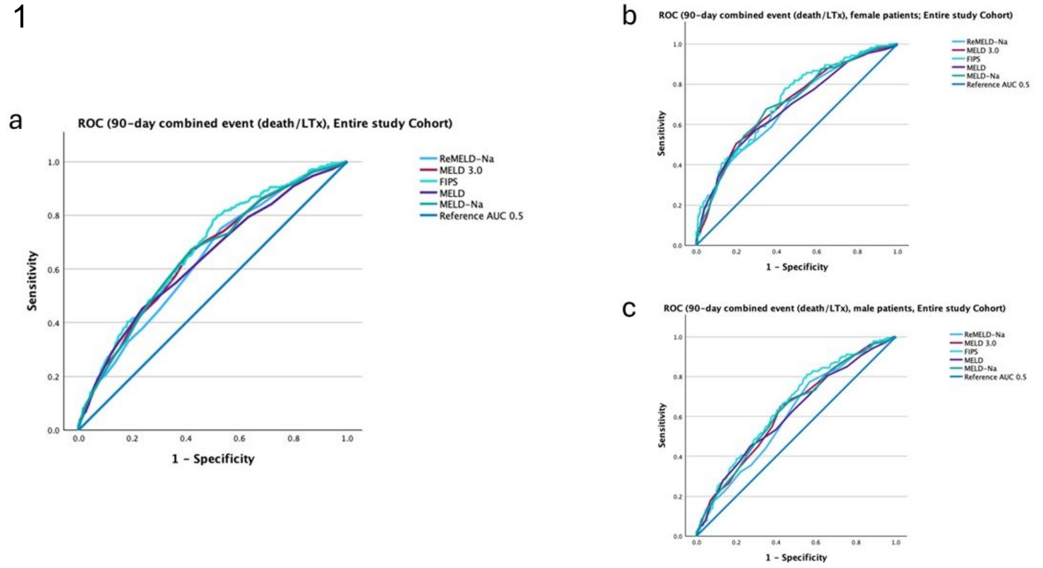

Fig. S1: Results of the Receiver operating characteristic (ROC) analysis in the entire study cohort with ROC curves displayed for ReMELD-Na, MELD 3.0, FIPS, MELD and MELD-Na for the entire cohort (a) as well as the female (b) and male subcohorts (c) separately. Outcome: 90-day combined event (Death/LTx).

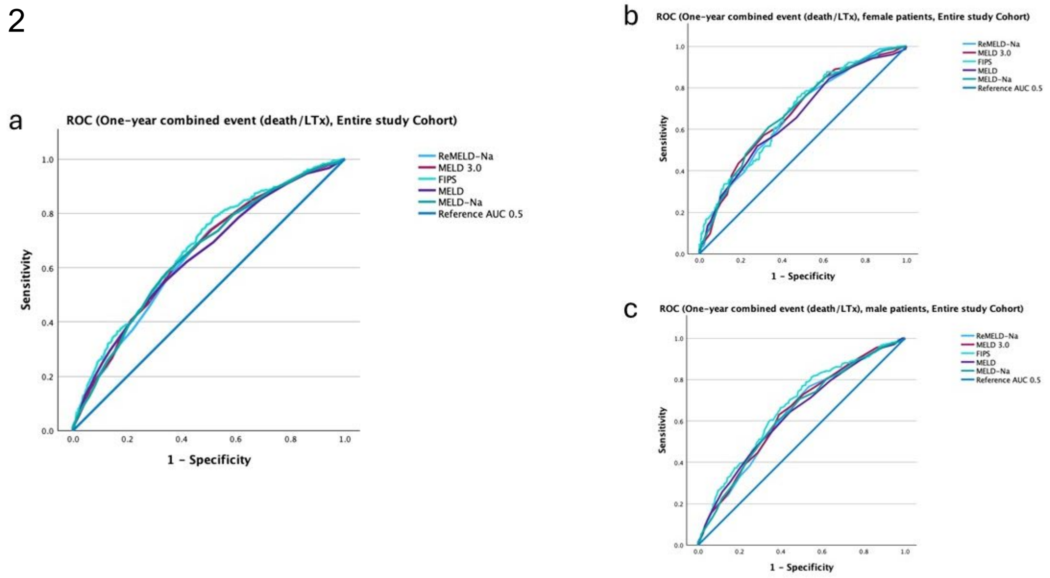

Fig. S2: Results of the Receiver operating characteristic (ROC) analysis in the entire study cohort with ROC curves displayed for ReMELD-Na, MELD 3.0, FIPS, MELD and MELD-Na for the entire cohort (a) as well as the female (b) and male subcohorts (c) separately. Outcome: One-year combined event (Death/LTx).

Supplementary Figure 3

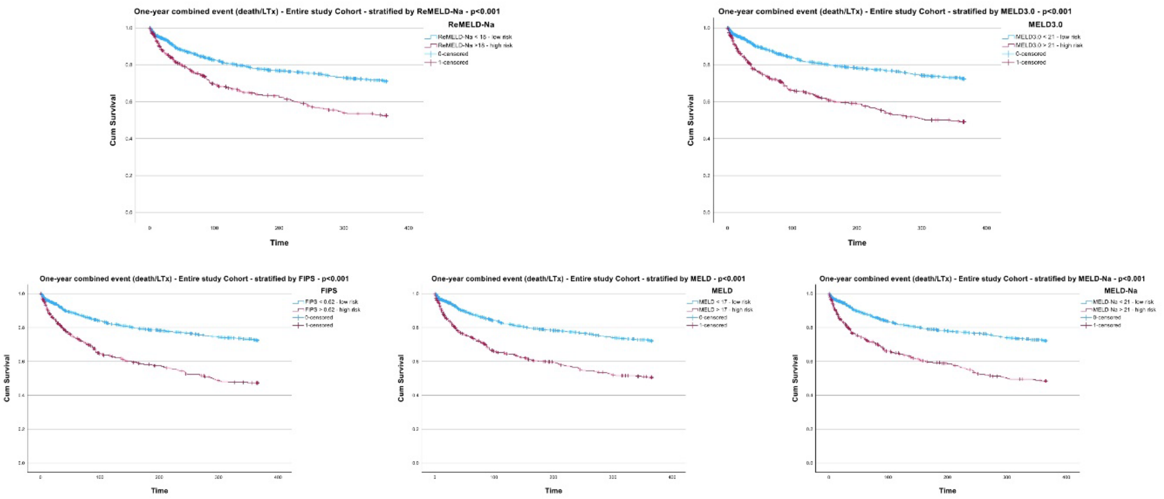

Fig. S3: Kaplan-Meier curves showing one-year combined event (Death/LTx)-free survival for high- vs. low-risk groups, stratified by the 75th percentile of each scoring system. Cutoff values used to define high-risk groups were: ReMELD-Na = 18, MELD 3.0 = 21, FIPS = 0.62, MELD = 17, and MELD-Na = 21. Level of significance for each Kaplan-Meier curve: p < 0.001 (log-rank test).

4a

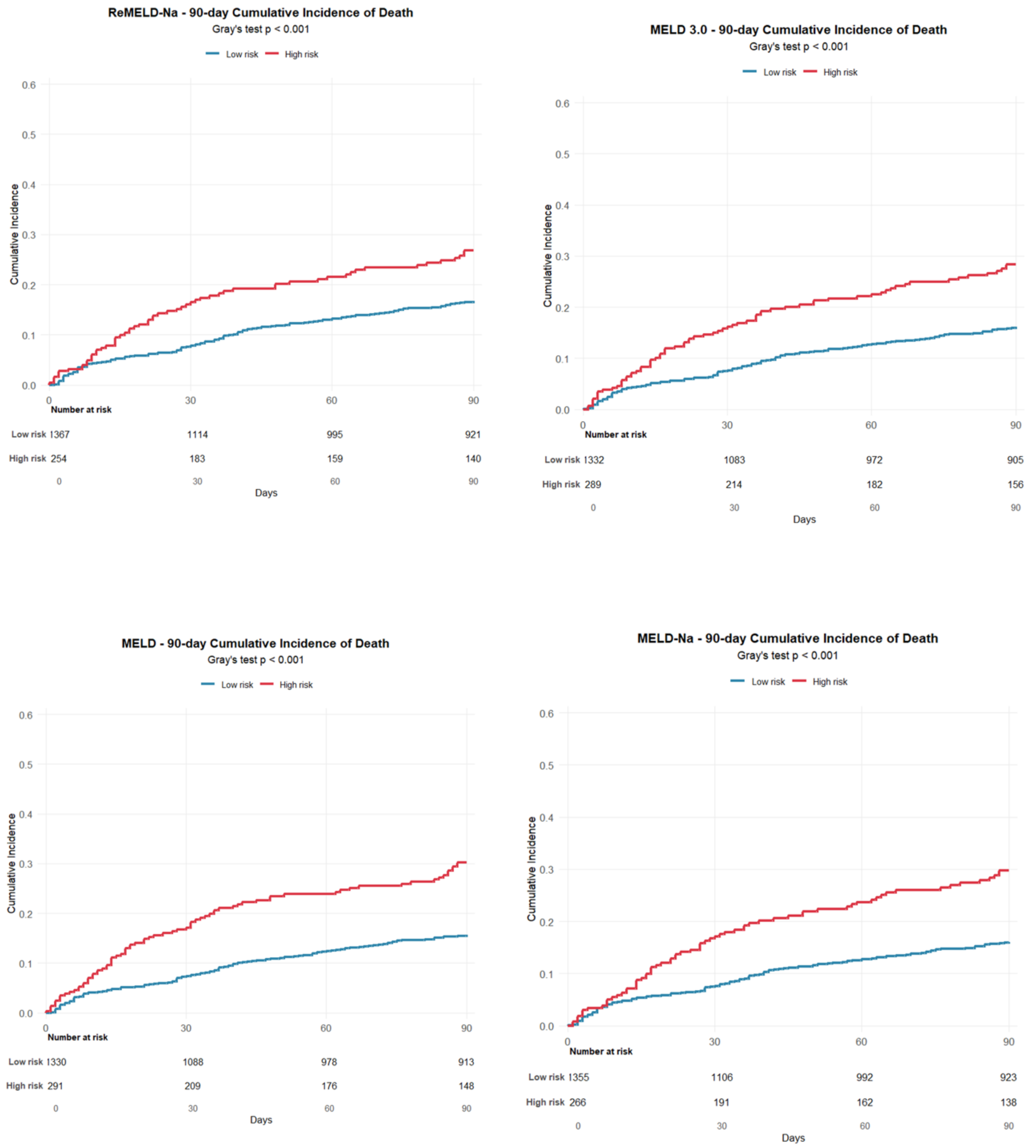
ReMELD-Na - 90-day Cumulative Incidence of Death
Gray's test p < 0.001
Low risk
High risk
Cumulative Incidence
0.0
0.1
0.2
0.3
0.4
0.5
0.6
0
30
60
90
Number at risk
Low risk 1367 1114 995 921
High risk 254 183 159 140
Days
MELD 3.0 - 90-day Cumulative Incidence of Death
Gray's test p < 0.001
Low risk
High risk
Cumulative Incidence
Number at risk
Low risk 1332 1083 972 905
High risk 289 214 182 156
Days
MELD - 90-day Cumulative Incidence of Death
Gray's test p < 0.001
Low risk
High risk
Cumulative Incidence
Number at risk
Low risk 1330 1088 978 913
High risk 291 209 176 148
Days
MELD-Na - 90-day Cumulative Incidence of Death
Gray's test p < 0.001
Low risk
High risk
Cumulative Incidence
Number at risk
Low risk 1355 1106 992 923
High risk 266 191 162 138
Days

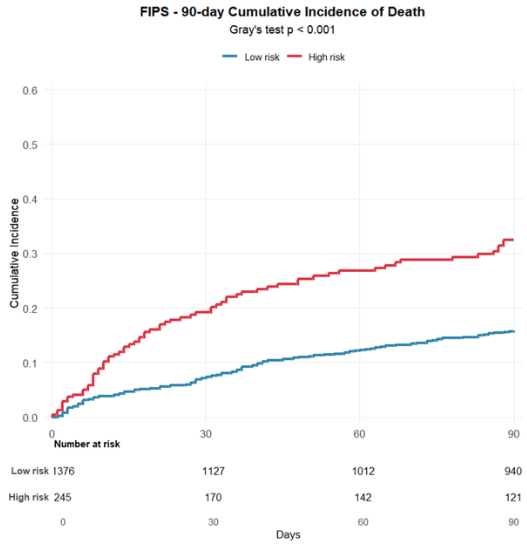

Fig. S4a: Fine-Gray proportional hazard analysis showing 90-day and one-year cumulative incidence of death under the competing risk of LTx for high- vs. low-risk groups, stratified by the 85th (90-day) percentile of each scoring system. Cutoff values used to define high-risk groups were: ReMELD-Na = 20, MELD 3.0 = 23, FIPS = 0.92, MELD = 19, and MELD-Na = 23. Level of significance for each analysis: $p < 0.001$ (Fine-Gray proportional hazard analysis).

4b

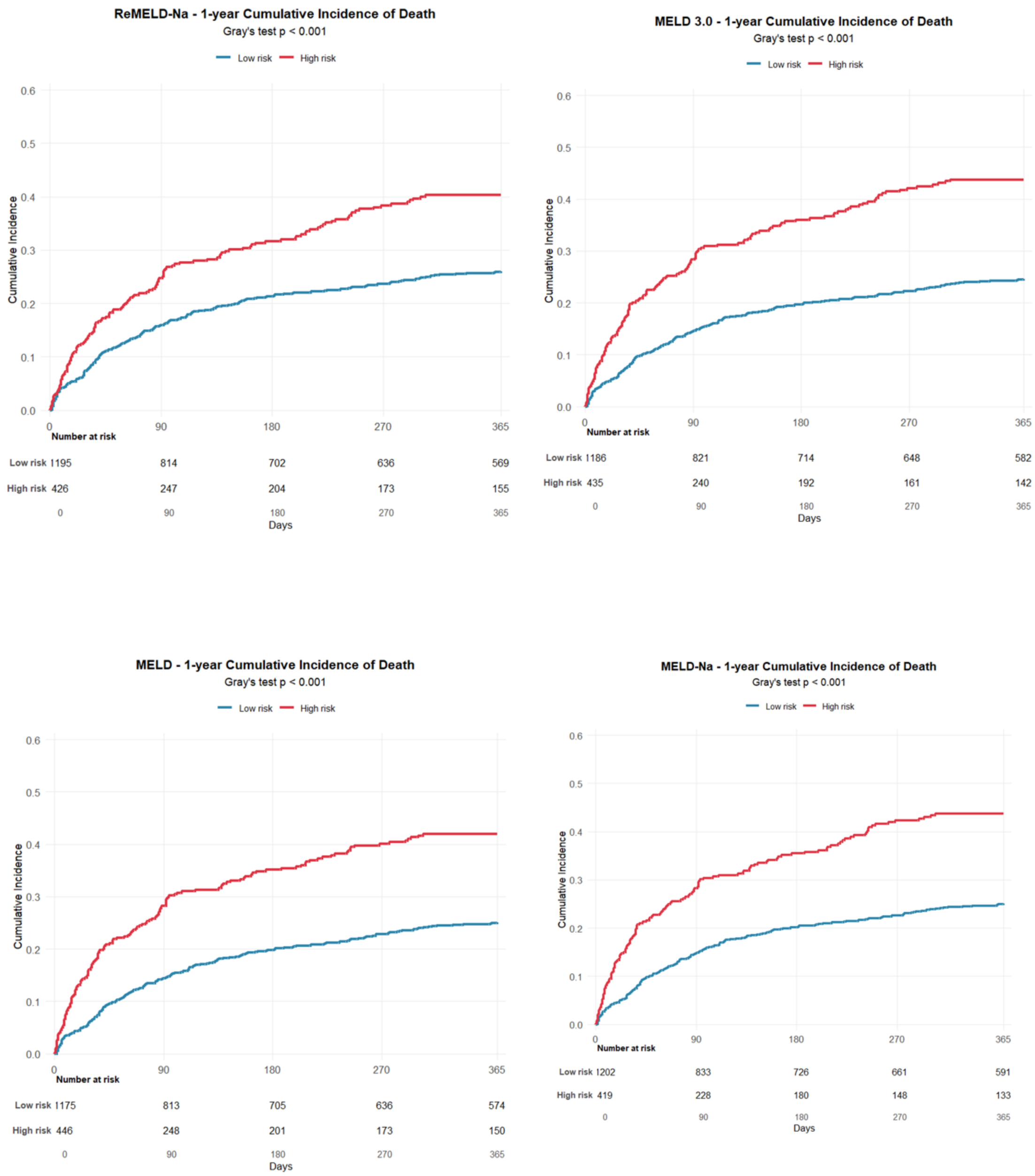
ReMELD-Na - 1-year Cumulative Incidence of Death
Gray's test p < 0.001
Low risk
High risk
Cumulative Incidence
Number at risk
Low risk 1195 814 702 636 569
High risk 426 247 204 173 155
Days
MELD 3.0 - 1-year Cumulative Incidence of Death
Gray's test p < 0.001
Low risk
High risk
Cumulative Incidence
Number at risk
Low risk 1186 821 714 648 582
High risk 435 240 192 161 142
Days
MELD - 1-year Cumulative Incidence of Death
Gray's test p < 0.001
Low risk
High risk
Cumulative Incidence
Number at risk
Low risk 1175 813 705 636 574
High risk 446 248 201 173 150
Days
MELD-Na - 1-year Cumulative Incidence of Death
Gray's test p < 0.001
Low risk
High risk
Cumulative Incidence
Number at risk
Low risk 1202 833 726 661 591
High risk 419 228 180 148 133
Days

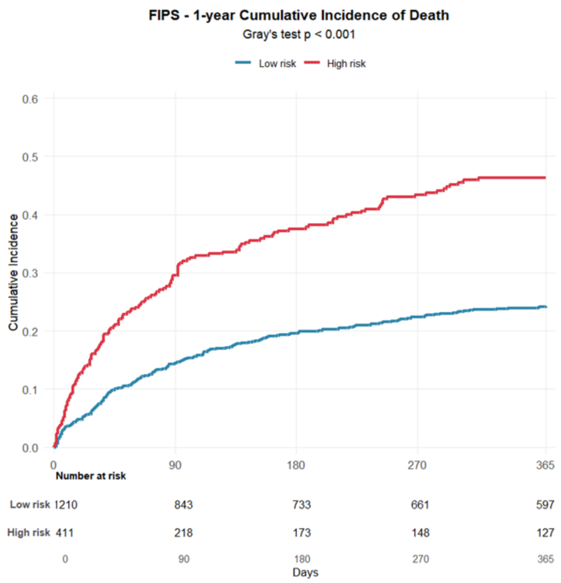

Fig. S4b: Fine-Gray proportional hazard analysis showing 90-day and one-year cumulative incidence of death under the competing risk of LTx for high- vs. low-risk groups, stratified by the 75th (one-year) percentile of each scoring system. Cutoff values used to define high-risk groups were: ReMELD-Na = 18, MELD 3.0 = 21, FIPS = 0.62, MELD = 17, and MELD-Na = 21. Level of significance for each analysis: $p < 0.001$ (Fine-Gray proportional hazard analysis).

Fig. S5a

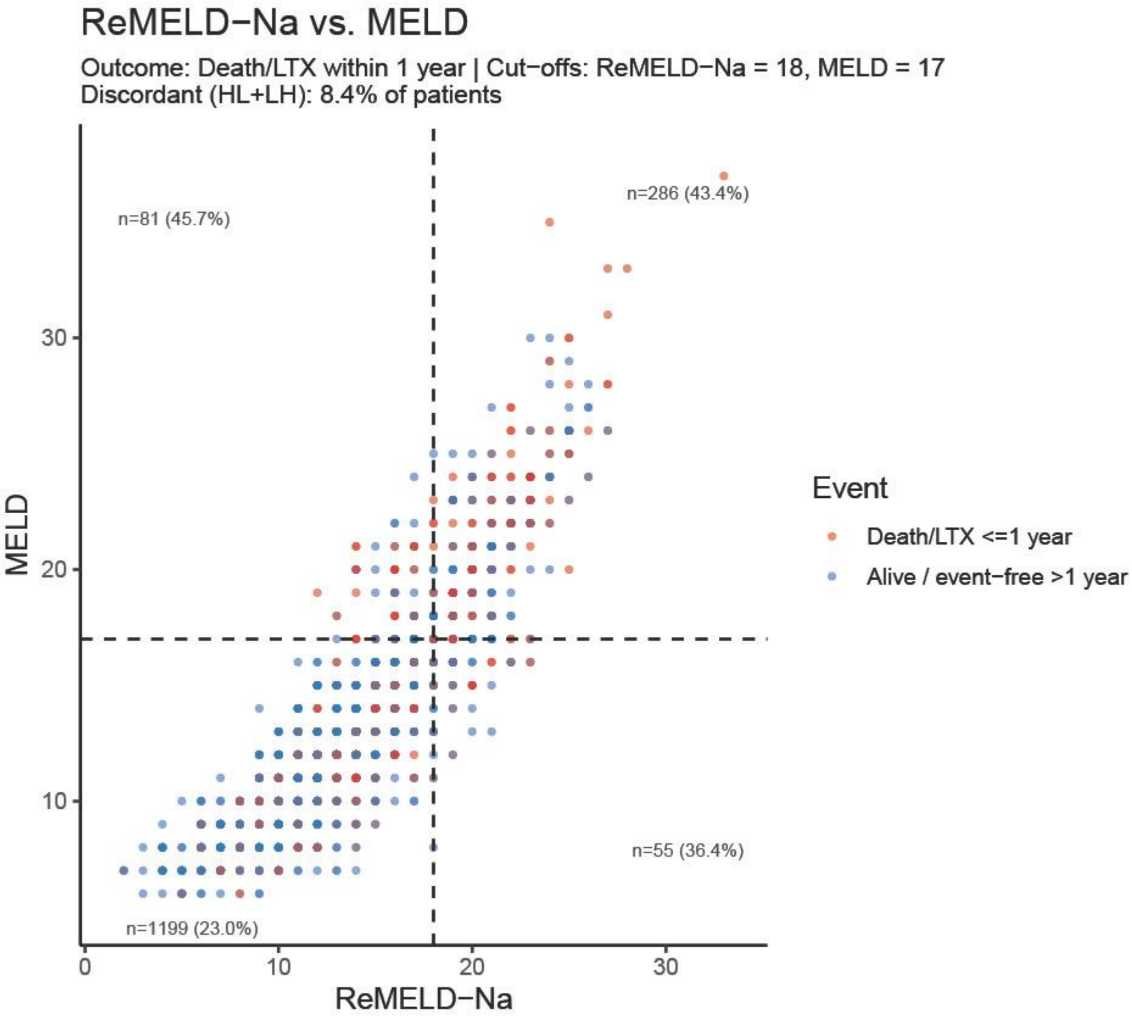

ReMELD−Na vs. MELD
Outcome: Death/LTX within 1 year | Cut−offs: ReMELD−Na = 18, MELD = 17
Discordant (HL+LH): 8.4% of patients
n=81 (45.7%)
n=286 (43.4%)
n=55 (36.4%)
n=1199 (23.0%)
MELD
ReMELD−Na
Event
Death/LTX <=1 year
Alive / event−free >1 year

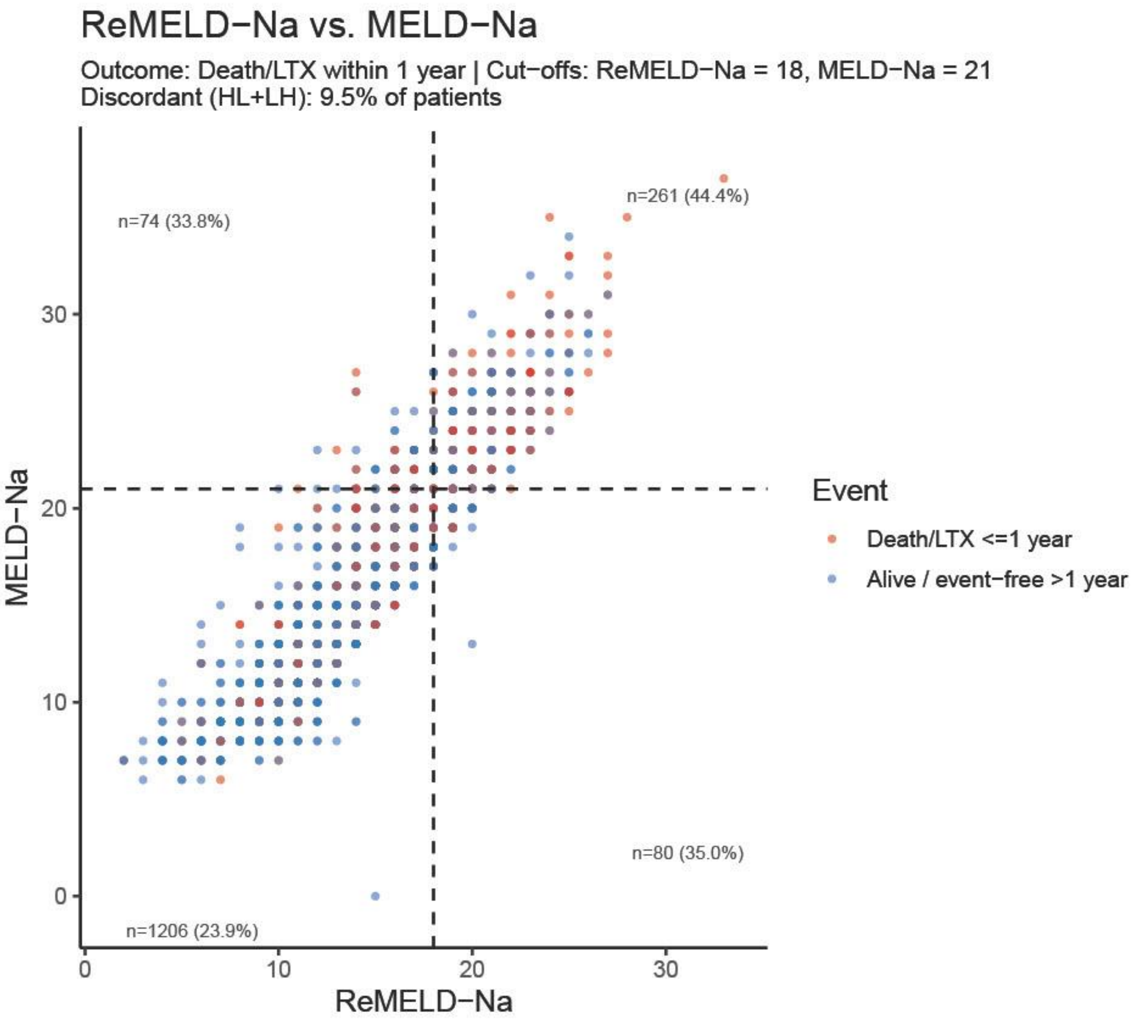

ReMELD−Na vs. MELD−Na
Outcome: Death/LTX within 1 year | Cut−offs: ReMELD−Na = 18, MELD−Na = 21
Discordant (HL+LH): 9.5% of patients
n=74 (33.8%)
n=261 (44.4%)
n=80 (35.0%)
n=1206 (23.9%)
MELD−Na
ReMELD−Na
0
10
20
30
Event
Death/LTX <=1 year
Alive / event−free >1 year

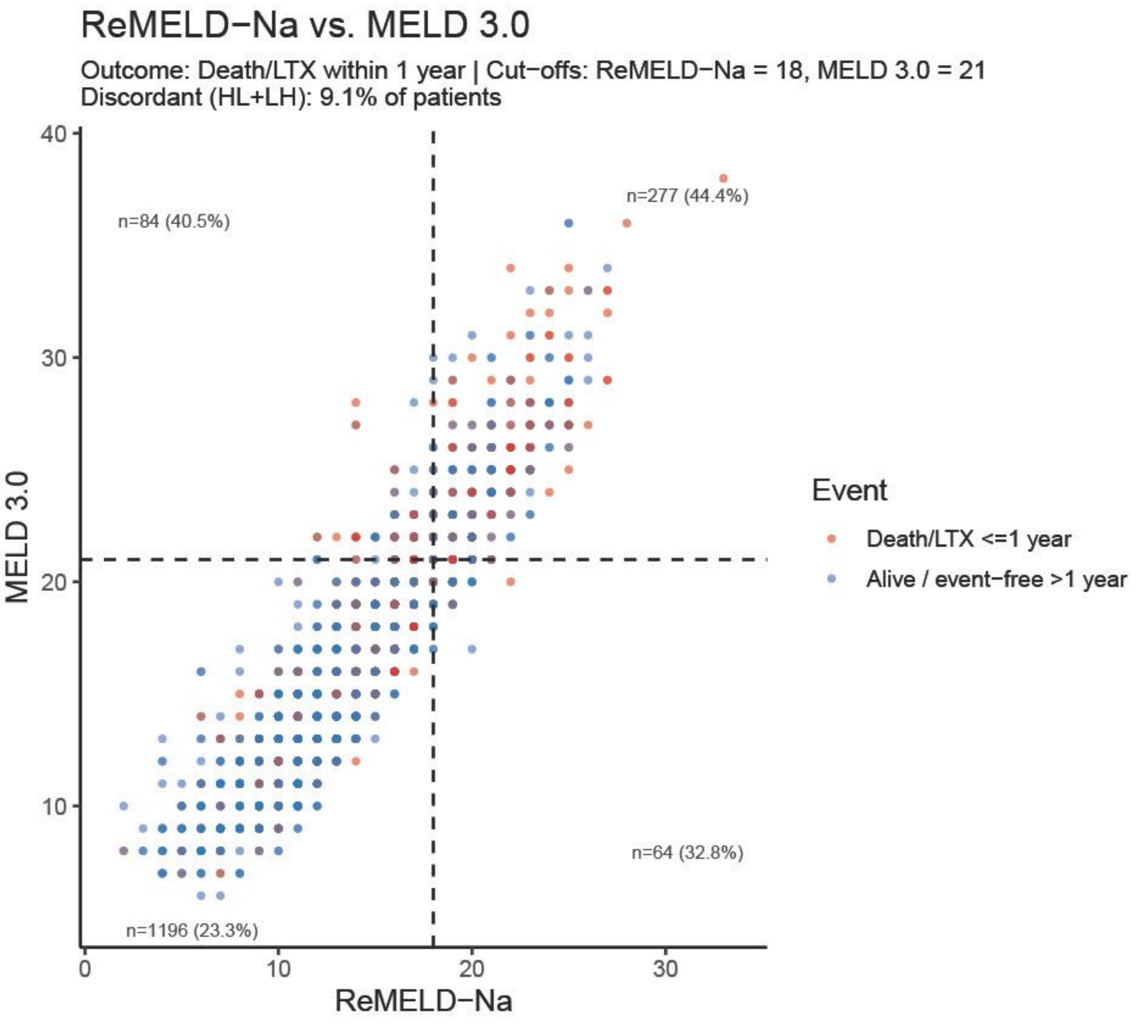

ReMELD−Na vs. MELD 3.0
Outcome: Death/LTX within 1 year | Cut−offs: ReMELD−Na = 18, MELD 3.0 = 21
Discordant (HL+LH): 9.1% of patients
n=84 (40.5%)
n=277 (44.4%)
n=64 (32.8%)
n=1196 (23.3%)
MELD 3.0
ReMELD−Na
Event
Death/LTX <=1 year
Alive / event−free >1 year

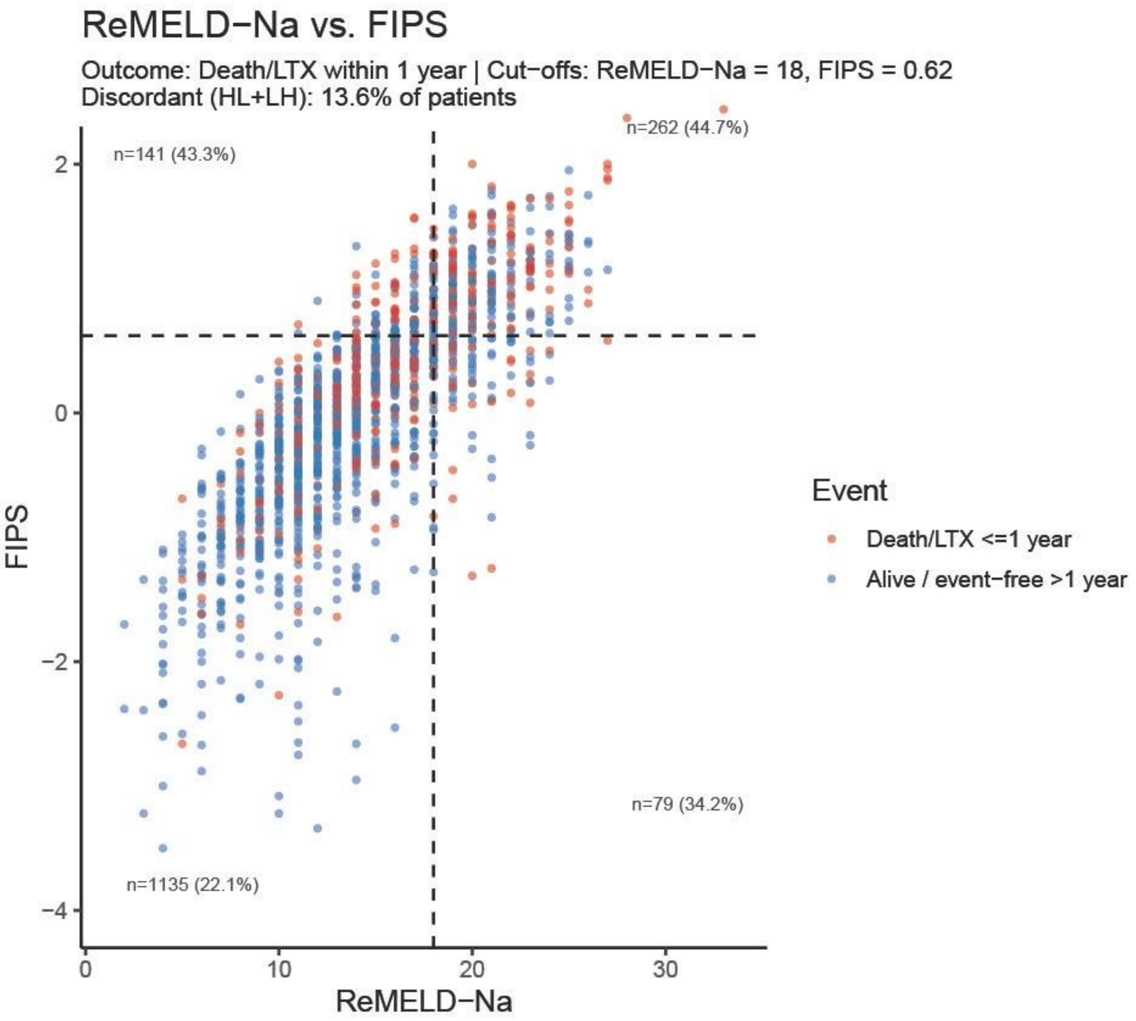
ReMELD−Na vs. FIPS
Outcome: Death/LTX within 1 year | Cut−offs: ReMELD−Na = 18, FIPS = 0.62
Discordant (HL+LH): 13.6% of patients
n=141 (43.3%)
n=262 (44.7%)
n=79 (34.2%)
n=1135 (22.1%)
FIPS
ReMELD−Na
2
0
−2
−4
0
10
20
30
Event
Death/LTX <=1 year
Alive / event−free >1 year

Fig. S5b

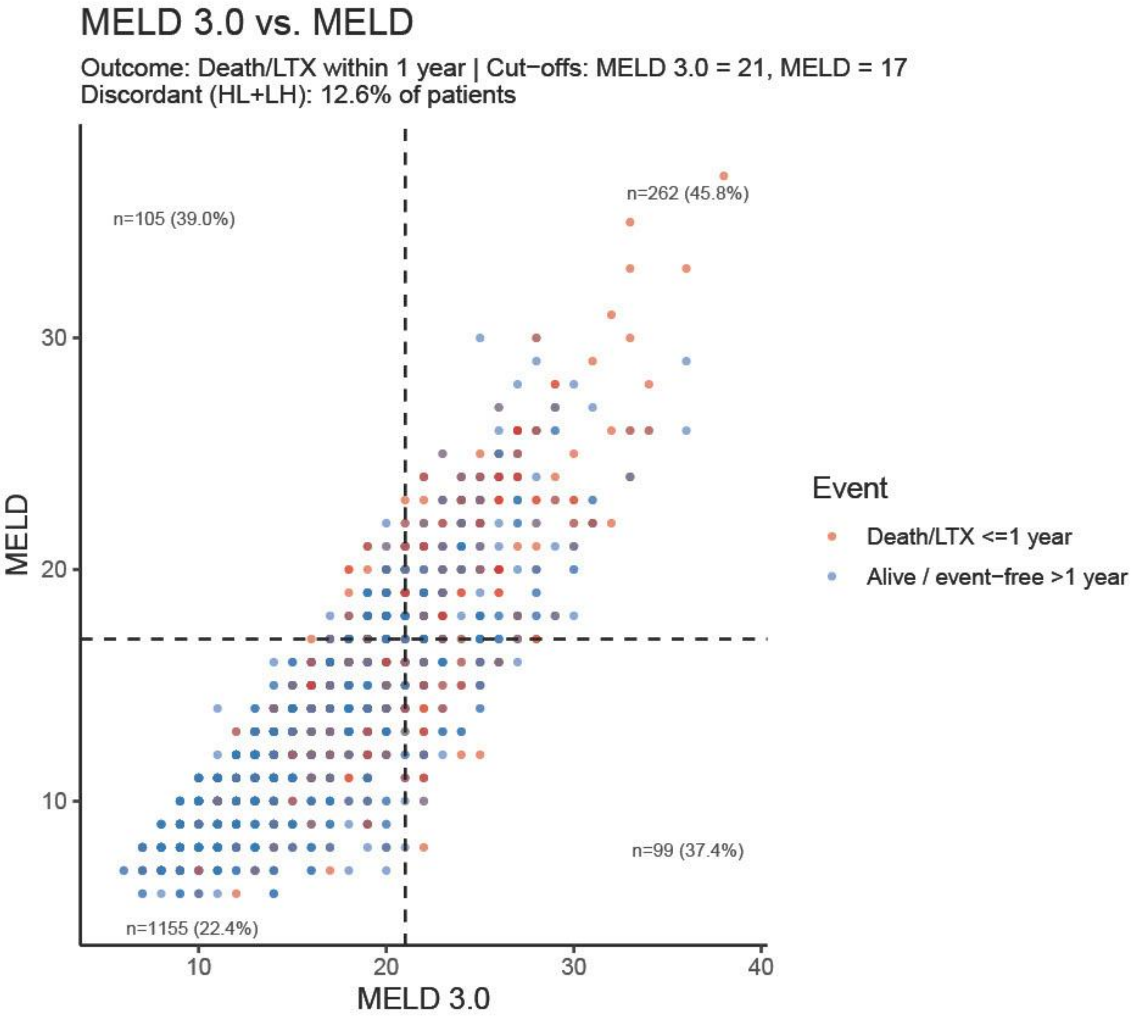

MELD 3.0 vs. MELD
Outcome: Death/LTX within 1 year | Cut−offs: MELD 3.0 = 21, MELD = 17
Discordant (HL+LH): 12.6% of patients
n=105 (39.0%)
n=262 (45.8%)
n=99 (37.4%)
n=1155 (22.4%)
MELD
MELD 3.0
10
20
30
40
Event
Death/LTX <=1 year
Alive / event−free >1 year

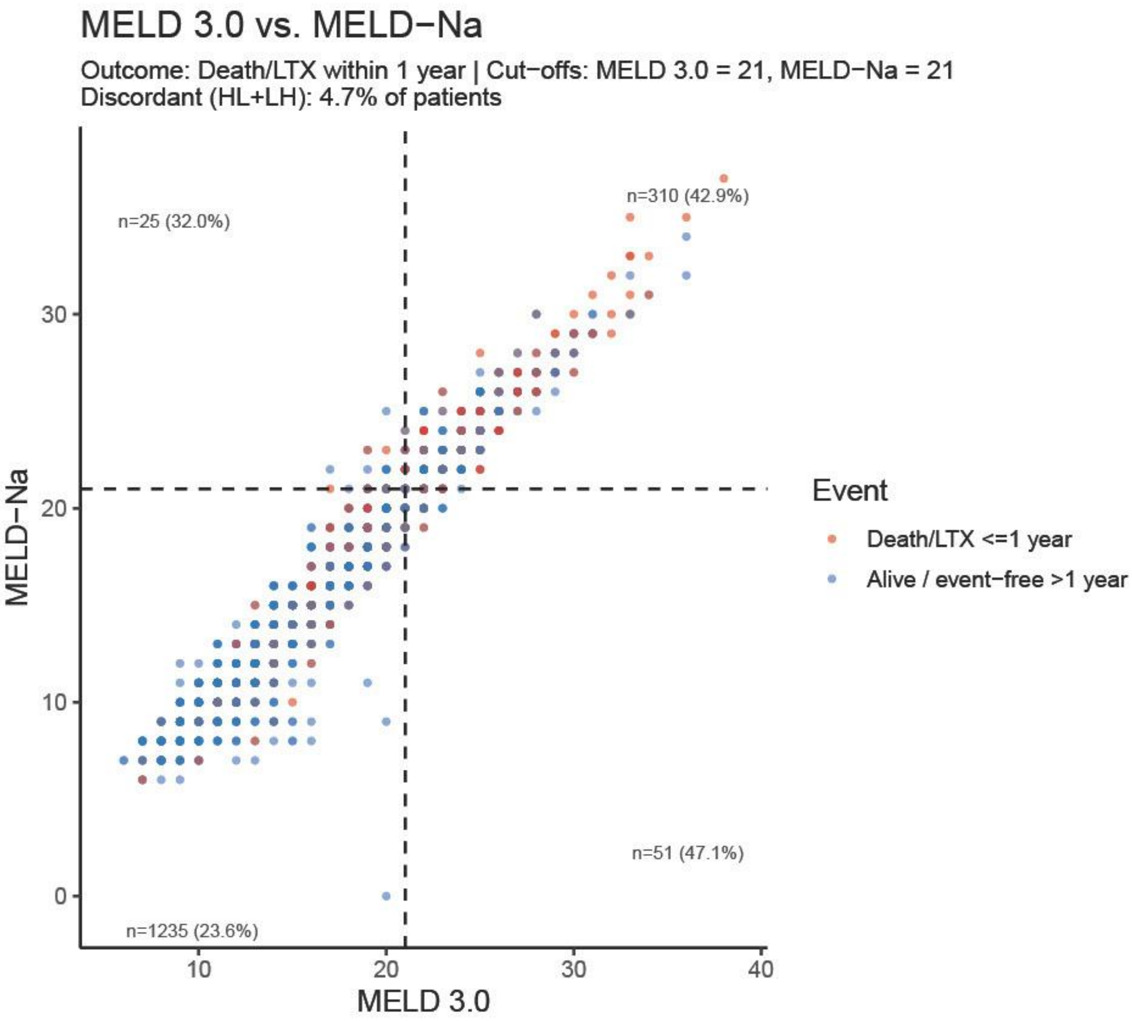
MELD 3.0 vs. MELD−Na
Outcome: Death/LTX within 1 year | Cut−offs: MELD 3.0 = 21, MELD−Na = 21
Discordant (HL+LH): 4.7% of patients
n=25 (32.0%)
n=310 (42.9%)
n=51 (47.1%)
n=1235 (23.6%)
MELD−Na
MELD 3.0
0
10
20
30
40
Event
Death/LTX <=1 year
Alive / event−free >1 year

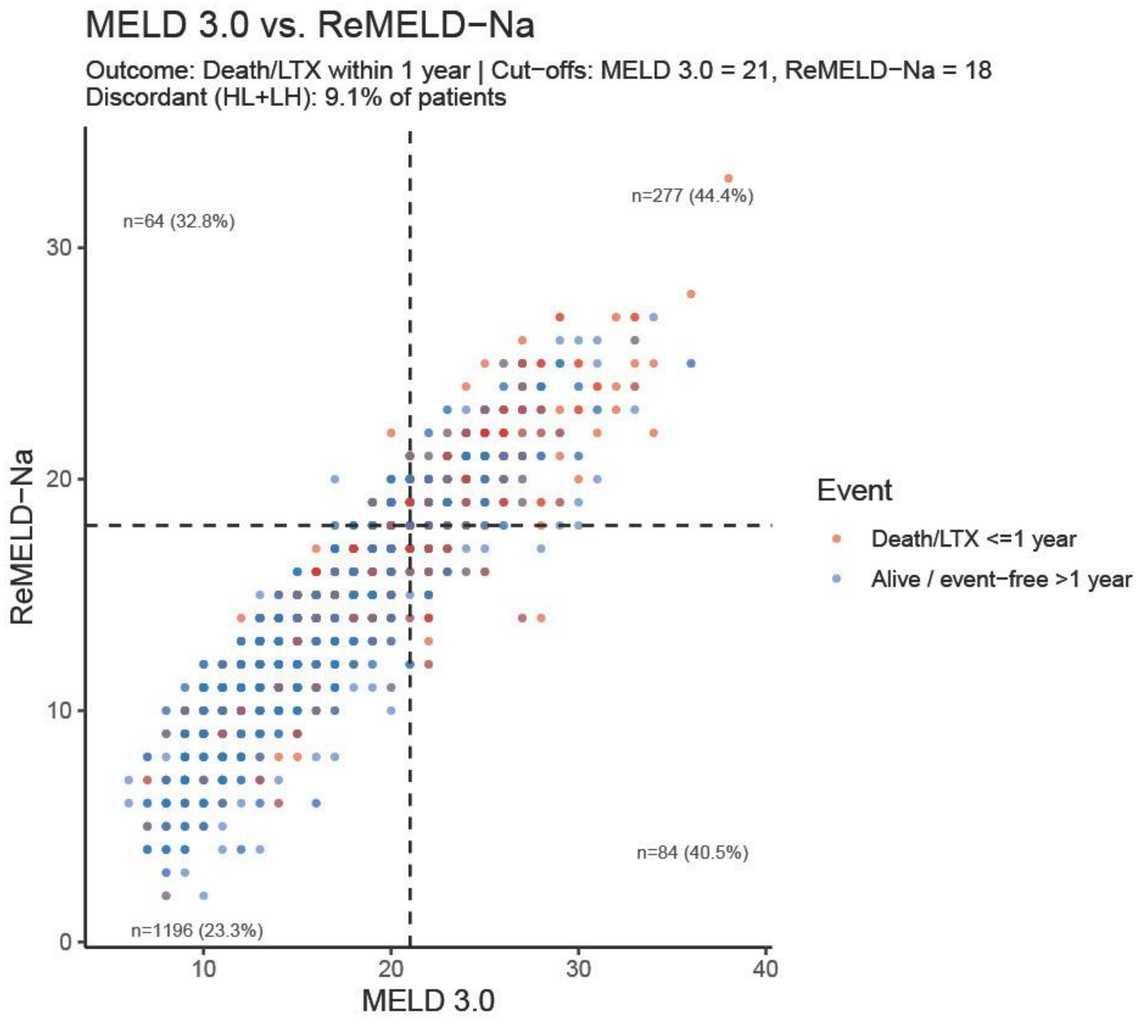
MELD 3.0 vs. ReMELD−Na
Outcome: Death/LTX within 1 year | Cut−offs: MELD 3.0 = 21, ReMELD−Na = 18
Discordant (HL+LH): 9.1% of patients
n=64 (32.8%)
n=277 (44.4%)
n=84 (40.5%)
n=1196 (23.3%)
ReMELD−Na
MELD 3.0
0
10
20
30
10
20
30
40
Event
Death/LTX <=1 year
Alive / event−free >1 year

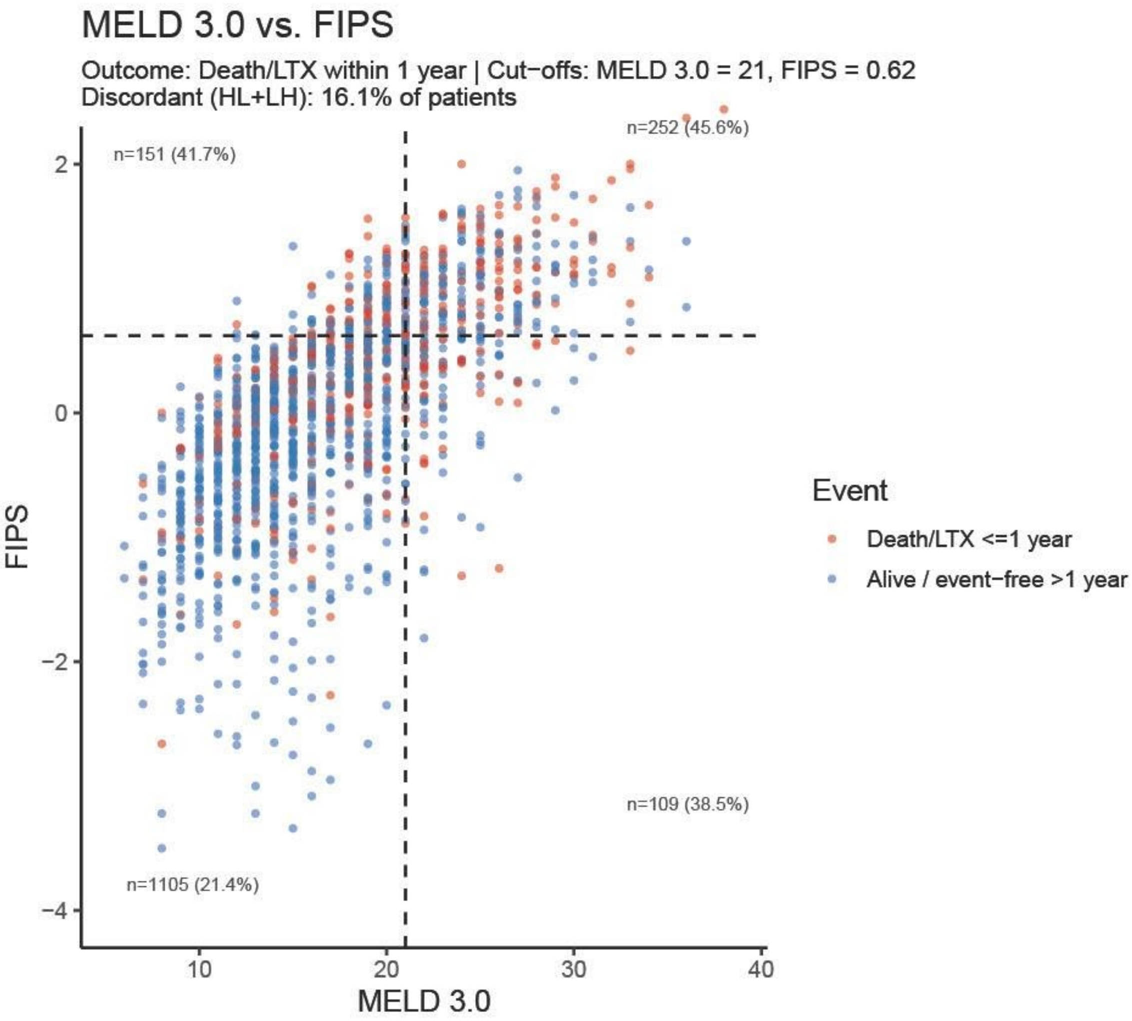

Fig. S5: Scatter plots of patients who reached the combined endpoint (Death/LTx) before censoring (orange) and those who did not (blue) within one year. **a)** Cube positions reflect ReMELD-Na (x-axis) and MELD, MELD-Na, MELD 3.0, or FIPS (y-axis). **b)** Cube positions reflect MELD 3.0 (x-axis) and MELD, MELD-Na, ReMELD-Na, or FIPS (y-axis). Based on high- vs. low-risk thresholds (ReMELD-Na = 18, MELD 3.0 = 21, FIPS = 0.62, MELD = 17, MELD-Na = 21), a 2×2 grid was defined: lower left = low/low-risk, lower right = high/low-risk, upper left = low/high-risk, upper right = high/high-risk. Lower right + upper left show discordant patients. Each quadrant shows n (patients) and % (events).

Fig. S6a

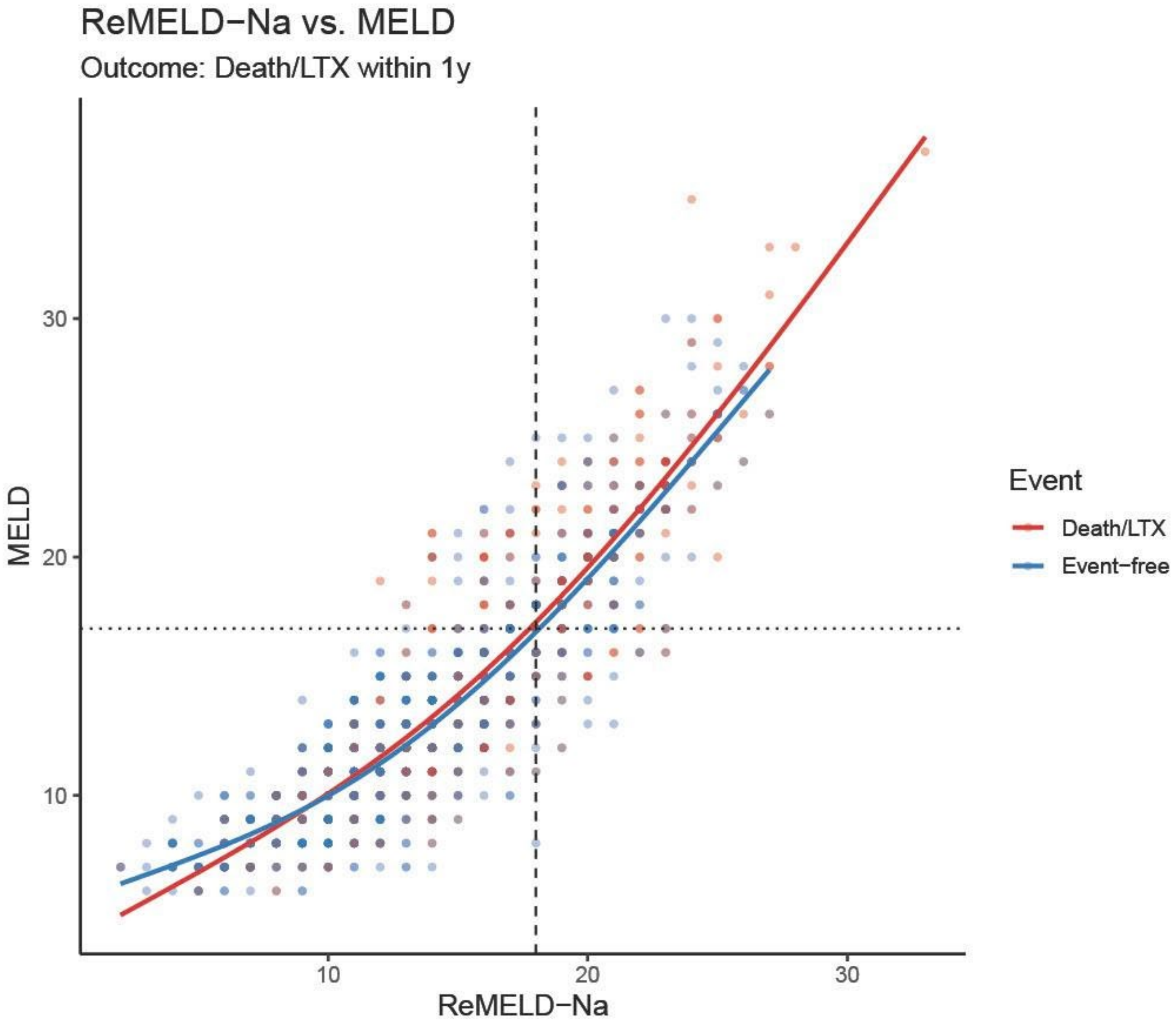

ReMELD−Na vs. MELD
Outcome: Death/LTX within 1y
MELD
30
20
10
10
20
30
ReMELD−Na
Event
Death/LTX
Event−free

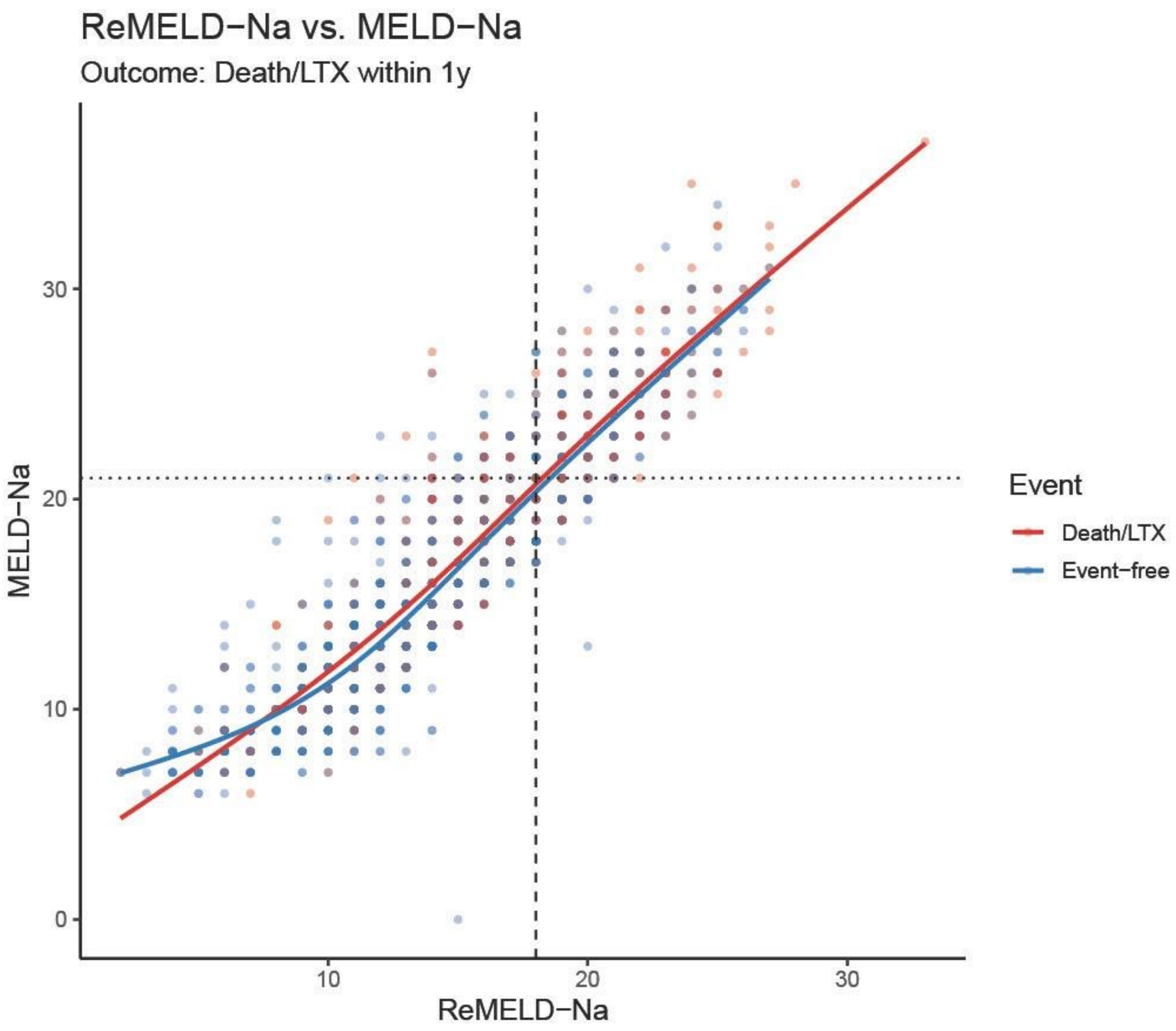
ReMELD−Na vs. MELD−Na
Outcome: Death/LTX within 1y
MELD−Na
ReMELD−Na
0
10
20
30
10
20
30
Event
Death/LTX
Event−free

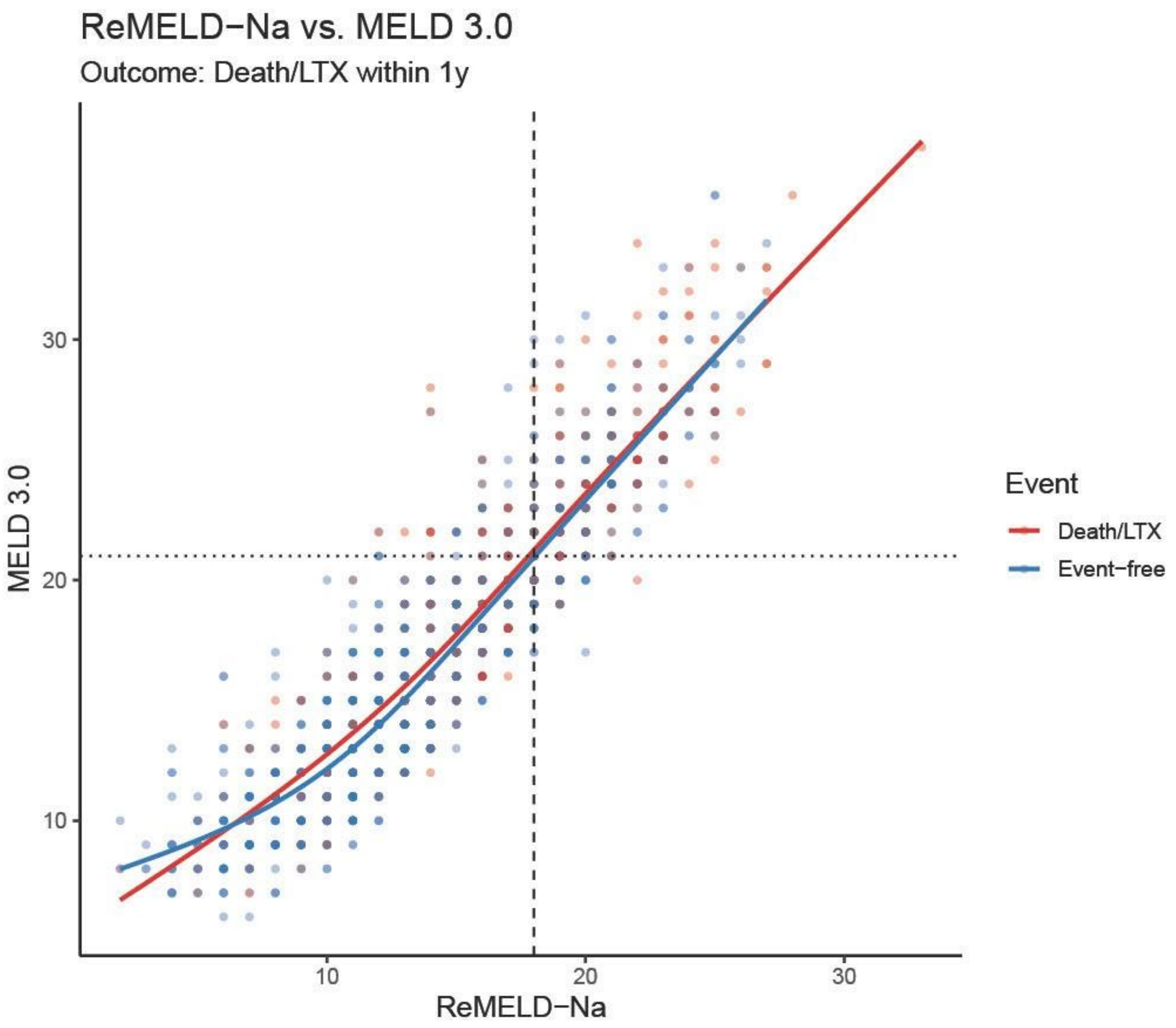
ReMELD−Na vs. MELD 3.0
Outcome: Death/LTX within 1y
MELD 3.0
ReMELD−Na
10
20
30
10
20
30
Event
Death/LTX
Event−free

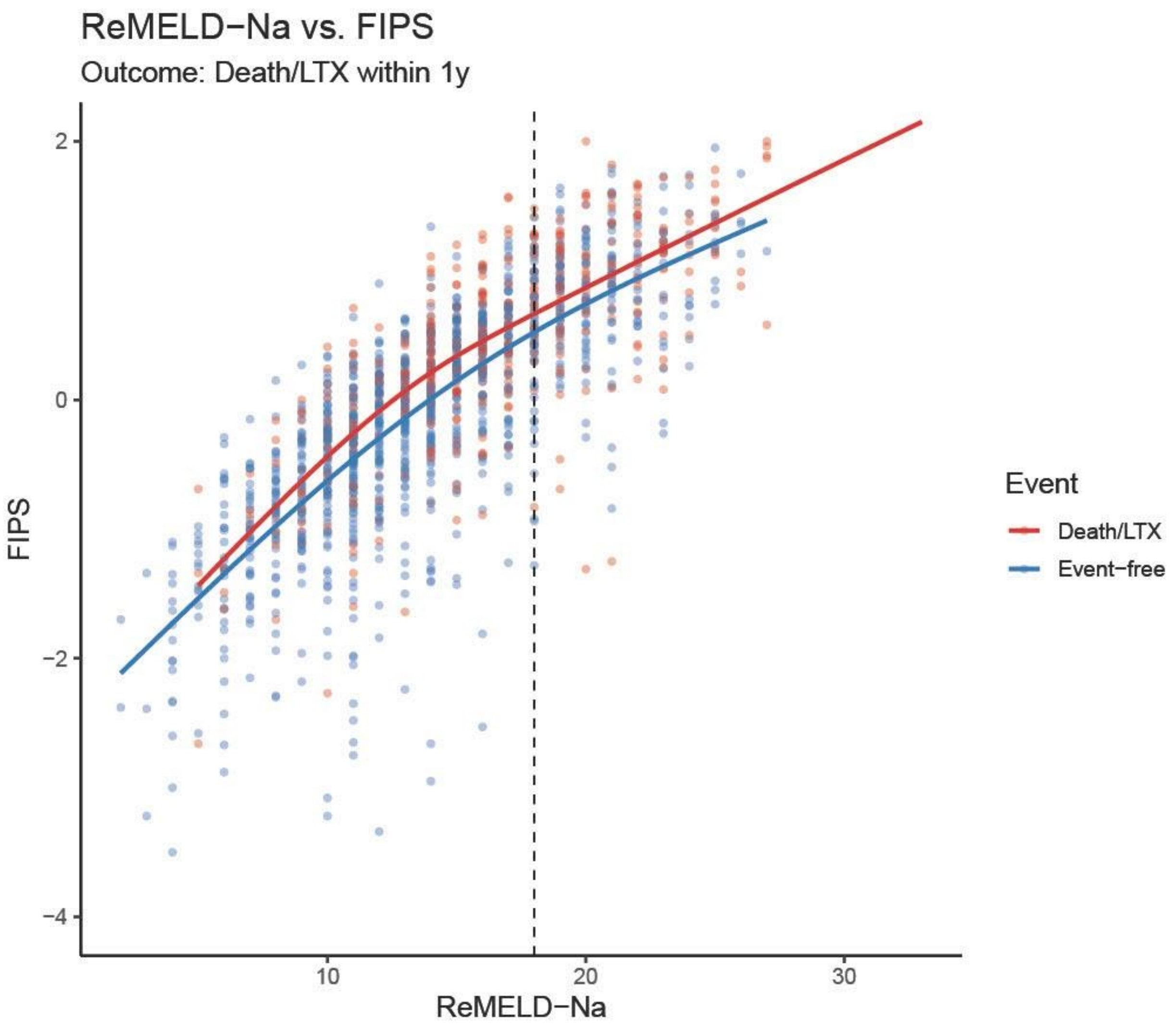
ReMELD−Na vs. FIPS
Outcome: Death/LTX within 1y
2
0
−2
−4
FIPS
10
20
30
ReMELD−Na
Event
Death/LTX
Event−free

Fig. S6b

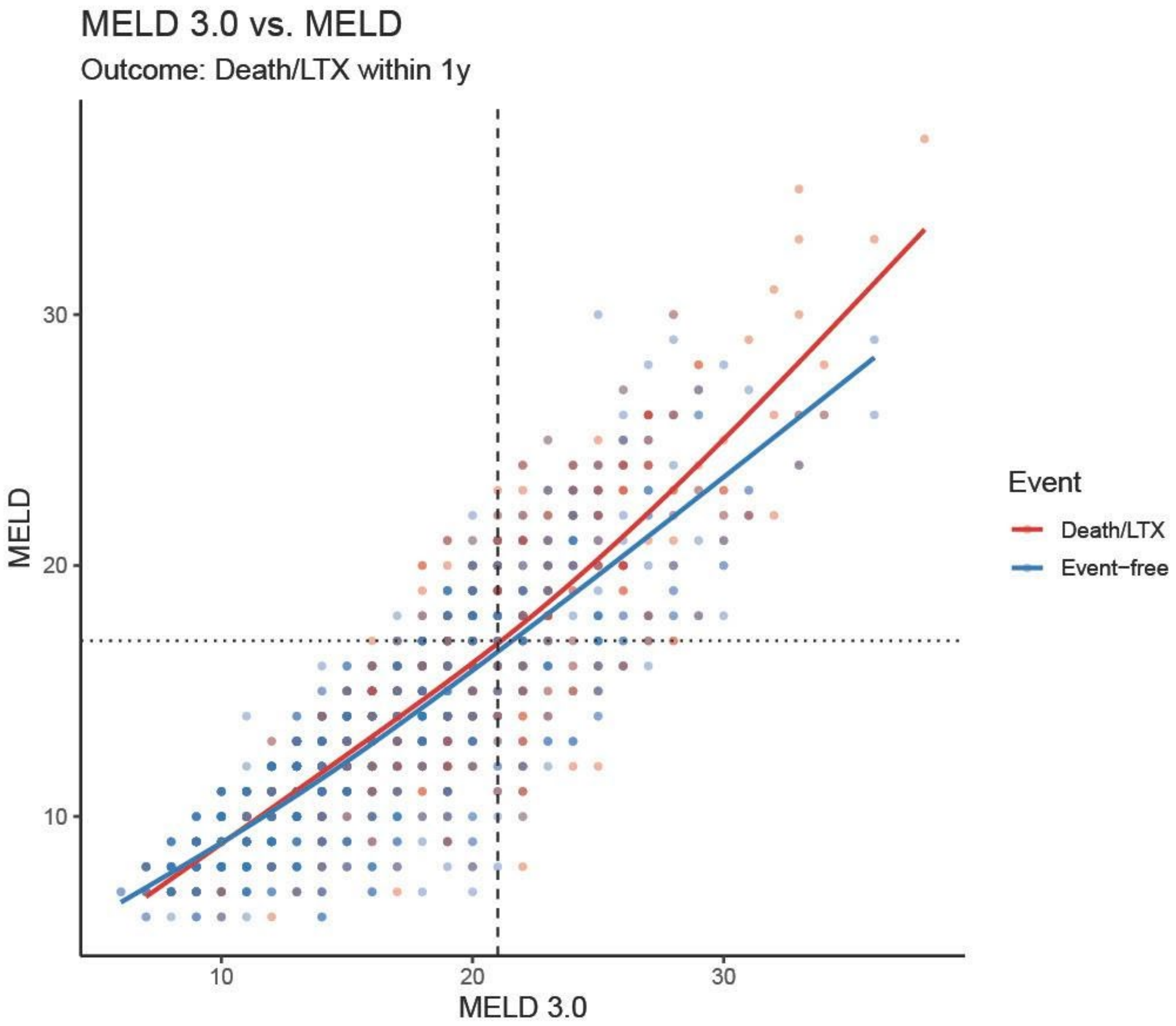
MELD 3.0 vs. MELD
Outcome: Death/LTX within 1y
MELD
30
20
10
10
20
30
MELD 3.0
Event
Death/LTX
Event−free

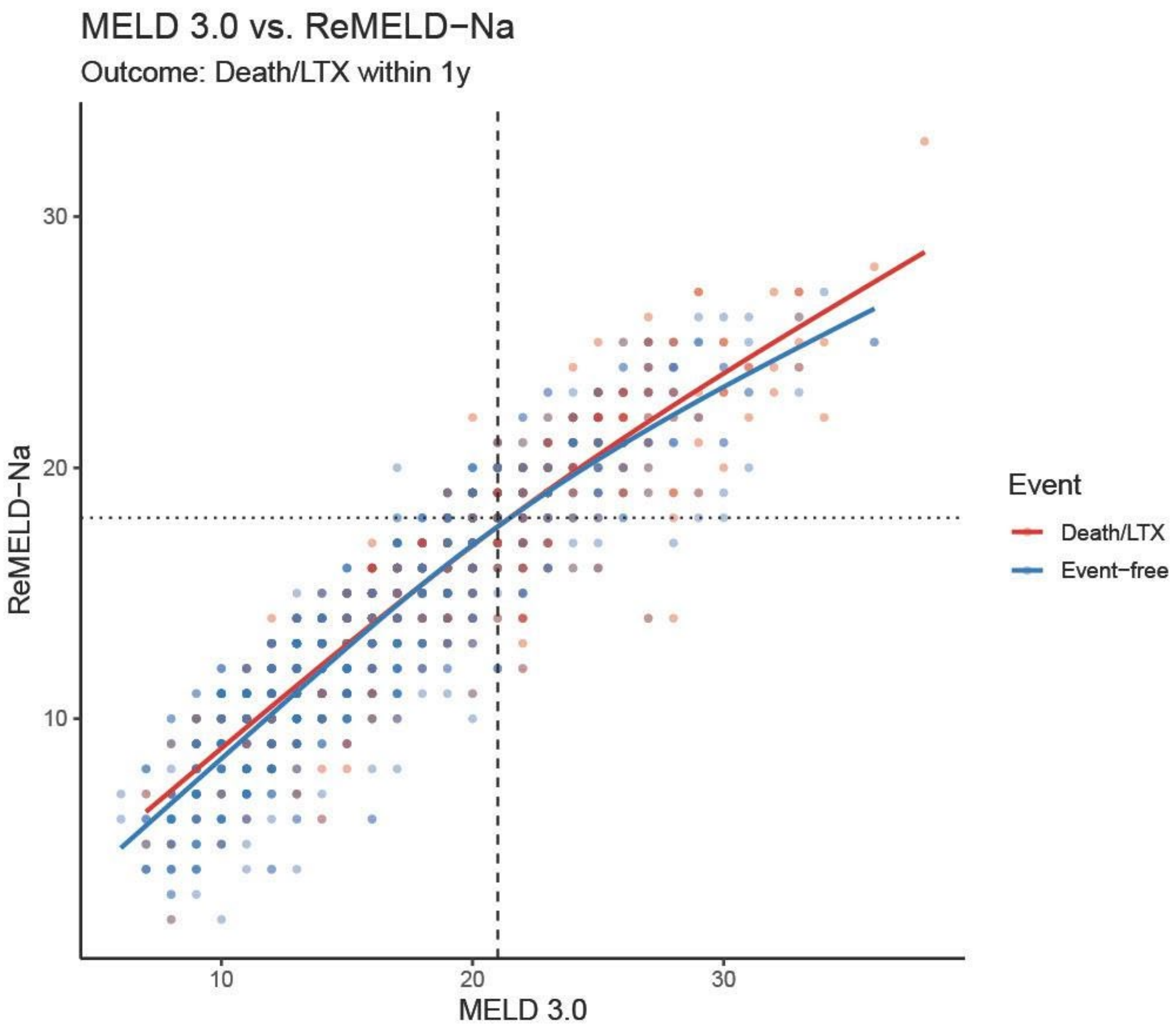
MELD 3.0 vs. ReMELD−Na
Outcome: Death/LTX within 1y
ReMELD−Na
MELD 3.0
10
20
30
10
20
30
Event
Death/LTX
Event−free

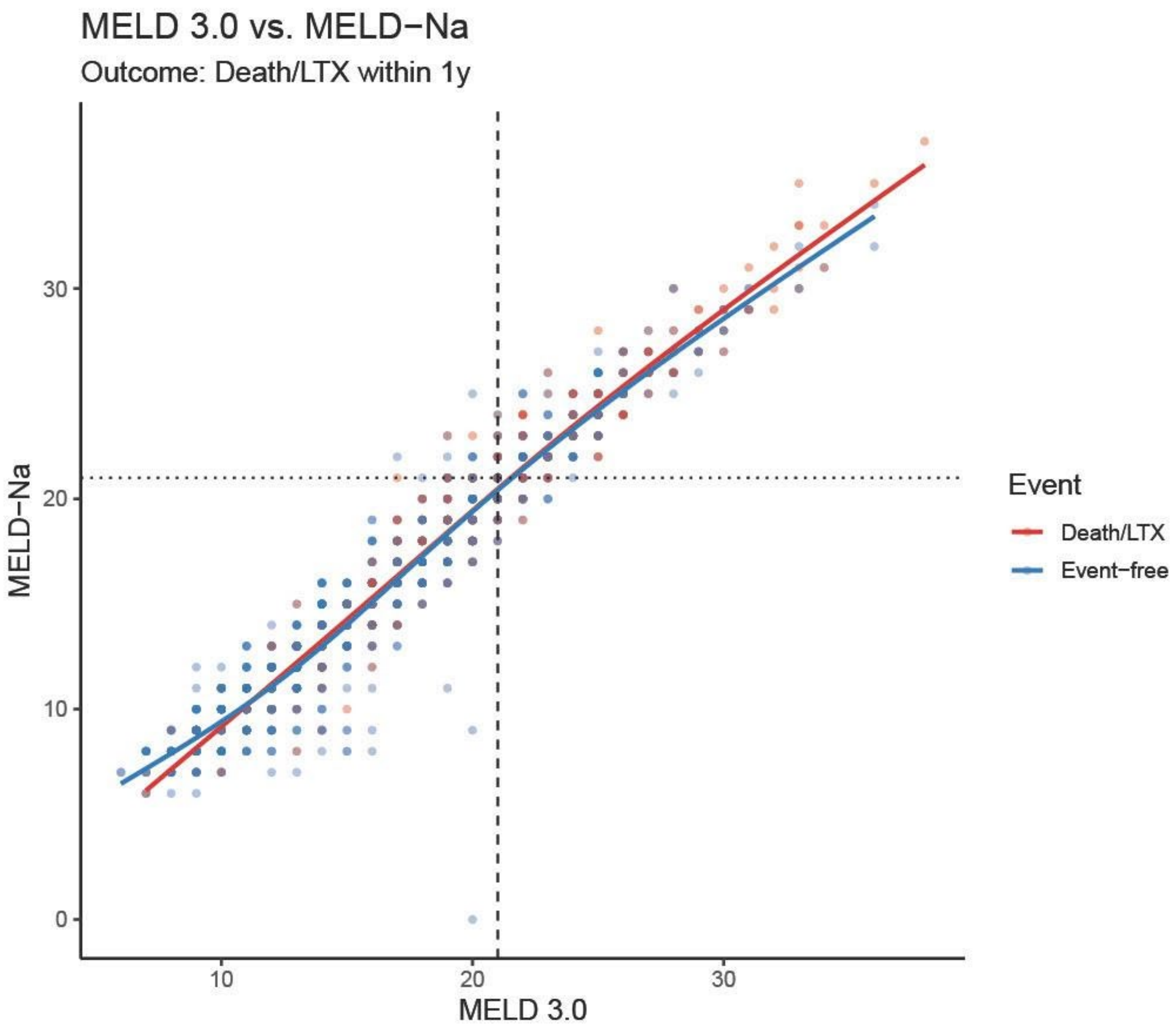
MELD 3.0 vs. MELD−Na
Outcome: Death/LTX within 1y
MELD−Na
0
10
20
30
MELD 3.0
10
20
30
Event
Death/LTX
Event−free

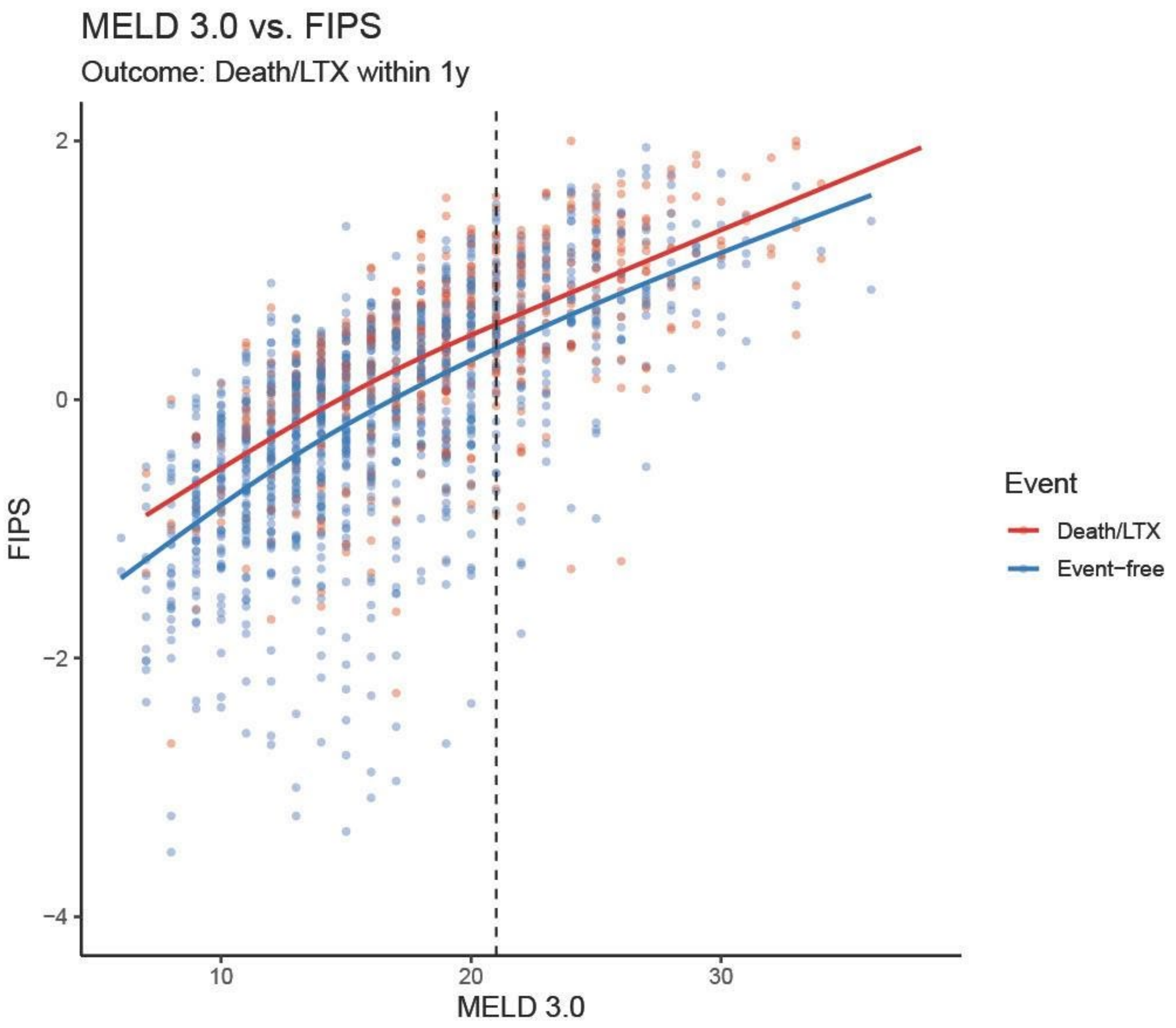

Fig. S6: Descriptive score-vs-score spline smooth analyses of patients who reached the combined endpoint (death/LTx) before censoring or loss-to follow-up (orange) and those who did not (blue) within one year, depicting the association between prognostic scores and events. **a)** Cube positions reflect ReMELD-Na (x-axis) and MELD, MELD-Na, MELD 3.0, or FIPS (y-axis). **b)** Cube positions reflect MELD 3.0 (x-axis) and MELD, MELD-Na, ReMELD-Na, or FIPS (y-axis).

Supplementary tables

1a)						
**Entire Cohort**	**Female (n = 579)**	**Male (n = 1042)**	**Difference (ROC-AUC female-male)**	**Female (n = 579)**	**Male (n = 1042)**	**Difference (ROC-AUC female-male)**
**(90-day combined event (Death/LTx))**	**No alcohol-related cirrhosis**	**No alcohol-related cirrhosis**		**Alcohol-related cirrhosis**	**Alcohol-related cirrhosis**	
**ReMELD-Na**	0.760	0.682	0.078	0.548	0.576	-0.028
**MELD 3.0**	0.793	0.711	0.082	0.551	0.591	-0.04
**FIPS**	0.799	0.733	0.066	0.579	0.614	-0.035
**MELD**	0.765	0.707	0.058	0.549	0.572	-0.023
**MELD-Na**	0.788	0.697	0.091	0.561	0.599	-0.038

1b)						
**Entire Cohort**	**Female (n = 579)**	**Male (n = 1042)**	**Difference (ROC-AUC female-male)**	**Female (n = 579)**	**Male (n = 1042)**	**Difference (ROC-AUC female-male)**
**(90-day combined event (Death/LTx))**	**Age < 59 years**	**Age < 59 years**		**Age > 59 years**	**Age > 59 years**	
**ReMELD-Na**	0.674	0.655	0.019	0.687	0.572	0.115
**MELD 3.0**	0.691	0.667	0.024	0.707	0.598	0.109
**FIPS**	0.697	0.662	0.035	0.701	0.62	0.081
**MELD**	0.665	0.647	0.018	0.69	0.585	0.105
**MELD-Na**	0.703	0.675	0.028	0.703	0.592	0.111

1c)						
**Entire Cohort**	**Female (n = 579)**	**Male (n = 1042)**	**Difference (ROC-AUC female-male)**	**Female (n = 579)**	**Male (n = 1042)**	**Difference (ROC-AUC female-male)**
**(90-day combined event (Death/LTx))**	**Creatinine < 1.26 mg/dl**	**Creatinine < 1.26 mg/dl**		**Creatinine > 1.26 mg/dl**	**Creatinine > 1.26 mg/dl**	
**ReMELD-Na**	0.694	0.641	0.053	0.570	0.541	0.029
**MELD 3.0**	0.704	0.644	0.060	0.600	0.577	0.023
**FIPS**	0.695	0.646	0.049	0.641	0.614	0.027
**MELD**	0.690	0.605	0.085	0.574	0.576	-0.002
**MELD-Na**	0.706	0.640	0.066	0.612	0.581	0.031

1d)						
**Entire Cohort**	**Female (n = 579)**	**Male (n = 1042)**	**Difference (ROC-AUC female-male)**	**Female (n = 579)**	**Male (n = 1042)**	**Difference (ROC-AUC female-male)**
**(90-day combined event (Death/LTx))**	**Albumin < 29 g/l**	**Albumin < 29 g/l**		**Albumin > 29 g/l**	**Albumin > 29 g/l**	
**ReMELD-Na**	0.698	0.566	0.132	0.662	0.66	0.002
**MELD 3.0**	0.739	0.591	0.148	0.672	0.67	0.002
**FIPS**	0.719	0.621	0.098	0.712	0.687	0.025
**MELD**	0.714	0.592	0.122	0.653	0.635	0.018
**MELD-Na**	0.736	0.593	0.143	0.67	0.667	0.003

Table S1: Results of the ROC analysis with ROC-AUC values displayed for ReMELD-Na, MELD 3.0, FIPS, MELD and MELD-Na stratified by sex and a) no alcohol-related cirrhosis/alcohol-related cirrhosis, b) age below or above median of 59 years, c) creatinine below or above median of 1.26 mg/dl, d) albumin below or above median of

29 g/l. Furthermore, differences of ROC-AUC between the female and male subcohort are displayed. Outcome: 90-day combined event (Death/LTx).

**2a)**						
**Entire Cohort**	**Female (n = 579)**	**Male (n = 1042)**	**Difference (ROC-AUC female-male)**	**Female (n = 579)**	**Male (n = 1042)**	**Difference (ROC-AUC female-male)**
**(one-year combined event (Death/LTx))**	**No alcohol-related cirrhosis**	**No alcohol-related cirrhosis**		**Alcohol-related cirrhosis**	**Alcohol-related cirrhosis**	
**ReMELD-Na**	0.706	0.691	0.015	0.64	0.608	0.032
**MELD 3.0**	0.736	0.696	0.04	0.638	0.615	0.023
**FIPS**	0.714	0.721	-0.007	0.652	0.639	0.013
**MELD**	0.699	0.689	0.01	0.63	0.607	0.023
**MELD-Na**	0.73	0.683	0.047	0.653	0.614	0.039

**2b)**						
**Entire Cohort**	**Female (n = 579)**	**Male (n = 1042)**	**Difference (ROC-AUC female-male)**	**Female (n = 579)**	**Male (n = 1042)**	**Difference (ROC-AUC female-male)**
**(one-year combined event (Death/LTx))**	**Age < 59 years**	**Age < 59 years**		**Age > 59 years**	**Age > 59 years**	
**ReMELD-Na**	0.665	0.643	0.022	0.669	0.628	0.041
**MELD 3.0**	0.673	0.645	0.028	0.682	0.638	0.044
**FIPS**	0.68	0.666	0.014	0.663	0.65	0.013
**MELD**	0.657	0.646	0.011	0.658	0.624	0.034
**MELD-Na**	0.681	0.644	0.037	0.686	0.627	0.059

2c)						
**Entire Cohort**	**Female (n = 579)**	**Male (n = 1042)**	**Difference (ROC-AUC female-male)**	**Female (n = 579)**	**Male (n = 1042)**	**Difference (ROC-AUC female-male)**
**(one-year combined event (Death/LTx))**	**Creatinine < 1.26 mg/dl**	**Creatinine < 1.26 mg/dl**		**Creatinine > 1.26 mg/dl**	**Creatinine > 1.26 mg/dl**	
**ReMELD-Na**	0.679	0.665	0.014	0.556	0.561	-0.005
**MELD 3.0**	0.672	0.676	-0.004	0.582	0.570	0.012
**FIPS**	0.652	0.652	0.000	0.615	0.627	-0.012
**MELD**	0.66	0.634	0.026	0.549	0.584	-0.035
**MELD-Na**	0.683	0.658	0.025	0.579	0.570	0.009

2d)						
**Entire Cohort**	**Female (n = 579)**	**Male (n = 1042)**	**Difference (ROC-AUC female-male)**	**Female (n = 579)**	**Male (n = 1042)**	**Difference (ROC-AUC female-male)**
**(one-year combined event (Death/LTx))**	**Albumin < 29g/l**	**Albumin < 29g/l**		**Albumin > 29g/l**	**Albumin > 29g/l**	
**ReMELD-Na**	0.676	0.587	0.089	0.656	0.687	-0.031
**MELD 3.0**	0.699	0.600	0.099	0.659	0.676	-0.017
**FIPS**	0.680	0.630	0.050	0.681	0.707	-0.026
**MELD**	0.682	0.602	0.080	0.633	0.666	-0.033
**MELD-Na**	0.706	0.579	0.127	0.659	0.669	-0.01

Table S2: Results of the ROC analysis with ROC-AUC values displayed for ReMELD-Na, MELD 3.0, FIPS, MELD and MELD-Na stratified by sex and a) no alcohol-related cirrhosis/alcohol-related cirrhosis, b) age below or above median of 59 years, c) creatinine below or above median of 1.26 mg/dl, d) albumin below or above median of 29 g/l. Furthermore, differences of ROC-AUC between the female and male subcohort are displayed. Outcome: one-year combined event (Death/LTx).

TIPS 2004-2013	Overall (n = 585)		TIPS 2014-2024	Overall (n = 1036)	
**90-day combined event (Death/LTx)**	AUC	Range	**90-day (combined event Death/LTx)**	AUC	Range
**ReMELD-Na**	0.591	0.542-0.640	**ReMELD-Na**	0.678	0.627-0.730
**MELD 3.0**	0.616	0.566-0.666	**MELD 3.0**	0.693	0.644-0.742
**FIPS**	0.641	0.593-0.690	**FIPS**	0.700	0.652-0.749
**MELD**	0.588	0.538-0.639	**MELD**	0.693	0.640-0.745
**MELD-Na**	0.619	0.569-0.669	**MELD-Na**	0.686	0.636-0.737

Table S3: Results of ROC analyses stratifying the cohort into patients receiving TIPS from 2004 to 2013 and 2014 to 2024. ROC-AUC values displayed for ReMELD-Na, MELD 3.0, FIPS, MELD and MELD-Na. Outcome: 90-day combined event (Death/LTx).

TIPS 2004-2013	Overall (n = 585)		TIPS 2014-2024	Overall (n = 1036)	
**One-year combined event (Death/LTx)**	AUC	Range	**One-Year combined event (Death/LTx)**	AUC	Range
**ReMELD-Na**	0.630	0.585-0.676	**ReMELD-Na**	0.658	0.618-0.698
**MELD 3.0**	0.641	0.595-0.686	**MELD 3.0**	0.662	0.624-0.701
**FIPS**	0.665	0.621-0.709	**FIPS**	0.671	0.631-0.710
**MELD**	0.624	0.578-0.670	**MELD**	0.660	0.620-0.700
**MELD-Na**	0.643	0.598-0.689	**MELD-Na**	0.656	0.617-0.695

Table S4: Results of ROC analyses stratifying the cohort into patients receiving TIPS from 2004 to 2013 and 2014 to 2024. ROC-AUC values displayed for ReMELD-Na, MELD 3.0, FIPS, MELD and MELD-Na. Outcome: One-year combined event (Death/LTx).

Bare Metal Stents	Overall (n = 187)	Covered Stents	Overall (n = 1434)

90-day combined event (Death/LTx)	AUC	Range	90-day combined event (Death/LTx)	AUC	Range
**ReMELD-Na**	0.635	0.544-0.716	**ReMELD-Na**	0.641	0.602-0.679
**MELD 3.0**	0.666	0.586-0.746	**MELD 3.0**	0.658	0.619-0.697
**FIPS**	0.710	0.635-0.785	**FIPS**	0.663	0.625-0.701
**MELD**	0.627	0.545-0.709	**MELD**	0.646	0.605-0.687
**MELD-Na**	0.668	0.588-0.747	**MELD-Na**	0.656	0.617-0.695

Table S5: Results of ROC analyses stratifying the cohort into patients receiving bare-metal stents and those receiving covered stents. ROC–AUC values are presented for ReMELD-Na, MELD 3.0, FIPS, MELD, and MELD-Na. Outcome: 90-day combined event (Death/LTx).

Bare Metal Stents	Overall (n = 187)		Covered Stents	Overall (n = 1434)	
**One-year combined event (Death/LTx)**	AUC	Range	**One-year combined event (Death/LTx)**	AUC	Range
**ReMELD-Na**	0.665	0.588-0.743	**ReMELD-Na**	0.649	0.617-0.681
**MELD 3.0**	0.689	0.613-0.766	**MELD 3.0**	0.654	0.622-0.685
**FIPS**	0.698	0.623-0.773	**FIPS**	0.665	0.634-0.697
**MELD**	0.658	0.580-0.737	**MELD**	0.646	0.614-0.679
**MELD-Na**	0.693	0.617-0.769	**MELD-Na**	0.651	0.619-0.682

Table S6: Results of ROC analyses stratifying the cohort into patients receiving bare-metal stents and those receiving covered stents. ROC–AUC values are presented for ReMELD-Na, MELD 3.0, FIPS, MELD, and MELD-Na. Outcome: One-year combined event (Death/LTx).

Score	Time	HR	95% CI	P value
**ReMELD-Na**	90-day	1.73	1.30–2.30	<0.001*
**MELD 3.0**	90-day	1.91	1.46–2.50	<0.001*
**MELD**	90-day	2.13	1.64–2.77	<0.001*
**MELD-Na**	90-day	2.00	1.53–2.63	<0.001*
**FIPS**	90-day	2.33	1.77–3.06	<0.001*
**ReMELD-Na**	one-year	1.70	1.39–2.08	<0.001*
**MELD 3.0**	one-year	2.07	1.70–2.53	<0.001*
**MELD**	one-year	1.97	1.61–2.40	<0.001*
**MELD-Na**	one-year	2.05	1.68–2.51	<0.001*
**FIPS**	one-year	2.24	1.83–2.73	<0.001*

Table S7: Results of the fine-gray proportional hazard analysis for each score in the entire study Cohort based on the division of the overall cohort at the 85th (90-day) and 75th (one-year) percentile of each score. Cutoff values used to define high-risk groups for the 90-day endpoint were: ReMELD-Na = 20, MELD 3.0 = 23, FIPS = 0.92, MELD = 19, and MELD-Na = 23. Cutoff values used to define high-risk groups for the one-year endpoint were: ReMELD-Na = 18, MELD 3.0 = 21, FIPS = 0.62, MELD = 17, and MELD-Na = 21. Outcome: 90-day and one-year cumulative incidence of death with the competing risk of LTx. Level of significance for each analysis: *p < 0.001 (Fine-Gray proportional hazard analysis).

90-day combined event (death/LTx))	Overall (n = 1621)		Female (n = 579)		Male (n = 1042)	
	PPV	NPV	PPV	NPV	PPV	NPV
**ReMELDNa**	28.3%	84.4%	37.8%	84.8%	24.4%	84.2%
**MELD 3.0**	29.8%	85.1%	37.6%	86.0%	25.5%	84.5%
**FIPS**	33.1%	85.2%	39.0%	85.1%	30.4%	85.2%
**MELD**	32.0%	85.6%	41.2%	85.8%	28.2%	85.4%
**MELDNa**	30.5%	84.9%	39.7%	85.2%	26.6%	84.8%

Table S8: Positive and negative predictive values (PPV and NPV) for each score in the entire study Cohort based on the division of the overall, female and male training cohort at the 85th percentile of each score. Outcome: 90-day combined event (Death/LTx).

One-year combined event (death/LTx))	Overall (n = 1621)		Female (n = 579)		Male (n = 1042)	
	PPV	NPV	PPV	NPV	PPV	NPV
**ReMELDNa**	39.9%	76.0%	42.9%	77.9%	38.7%	74.8%
**MELD 3.0**	42.8%	77.2%	45.6%	79.9%	41.3%	75.6%
**FIPS**	43.6%	77.0%	45.0%	78.7%	42.9%	76.1%
**MELD**	42.2%	77.1%	42.9%	78.3%	41.9%	76.4%
**MELDNa**	42.7%	76.9%	45.6%	78.6%	41.5%	75.8%

Table S9: Positive and negative predictive values (PPV and NPV) for each score in the entire study Cohort based on the division of the overall, female and male training cohort at the 85th percentile of each score. Outcome: one-year combined event (Death/LTx).

Supplementary references

Author names in bold designate shared co-first authorship.

1. Bloom S, Kemp W, Lubel J. Portal hypertension: pathophysiology, diagnosis and management. Intern. Med. J. 2015;45:16–26.
2. Iwakiri Y, Trebicka J. Portal hypertension in cirrhosis: Pathophysiological mechanisms and therapy. JHEP Rep. Innov. Hepatol. 2021;3:100316.
3. Gu W, Hortlik H, Erasmus H-P, *et al.* Trends and the course of liver cirrhosis and its complications in Germany: Nationwide population-based study (2005 to 2018). Lancet Reg. Health Eur. 2022;12:100240.
4. Volk ML, Tocco RS, Bazick J, *et al.* Hospital Readmissions Among Patients With Decompensated Cirrhosis. Off. J. Am. Coll. Gastroenterol. ACG 2012;107:247.
5. Allaire M, Walter A, Sutter O, *et al.* TIPS for management of portal-hypertension-related complications in patients with cirrhosis. Clin. Res. Hepatol. Gastroenterol. 2020;44:249–263.
6. Larrue H, D'Amico G, Olivas P, *et al.* TIPS prevents further decompensation and improves survival in patients with cirrhosis and portal hypertension in an individual patient data meta-analysis. J. Hepatol. 2023;79:692–703.
7. Trebicka J. Emergency TIPS in a Child-Pugh B patient: When does the window of opportunity open and close? J. Hepatol. 2017;66:442–450.
8. Gu W, Zeleke Y, Hortlik H, *et al.* Use and outcome of TIPS in hospitalized patients in Germany: A Nationwide study (2007-2018). Hepatol. Commun. 2023;7:e0237.
9. Ruf AE, Kremers WK, Chavez LL, *et al.* Addition of serum sodium into the MELD score predicts waiting list mortality better than MELD alone. Liver Transpl. 2005;11:336.
10. Biggins SW, Kim WR, Terrault NA, *et al.* Evidence-based incorporation of serum sodium concentration into MELD. Gastroenterology 2006;130:1652–1660.
11. **Nagai S, Chau LC,** Schilke RE, *et al.* Effects of Allocating Livers for Transplantation Based on Model for End-Stage Liver Disease–Sodium Scores on Patient Outcomes. Gastroenterology 2018;155:1451-1462.e3.

12. Kim WR, Mannalithara A, Heimbach JK, *et al.* MELD 3.0: The Model for End-Stage Liver Disease Updated for the Modern Era. Gastroenterology 2021;161:1887-1895.e4.

13. Ärzteblatt DÄG Redaktion Deutsches. Richtlinie gem. § 16 Abs. 1 S. 1 Nrn. 2 und 5 TPG für die Wartelistenführung und Organvermittlung zur Lebertransplantation Richtlinie gemäß § 16 Abs. 1 S. 1 Nrn. 2 u. 5 TPG für die Wartelistenführung und Organvermittlung zur Lebertransplantation (RL BÄK Leber). Dtsch. Ärztebl. 2025.

14. Rössle M. Liver: Early TIPS in patients with cirrhosis and variceal bleeding. Nat. Rev. Gastroenterol. Hepatol. 2010;7:536–538.

15. Franchis R de. Evolving Consensus in Portal Hypertension Report of the Baveno IV Consensus Workshop on methodology of diagnosis and therapy in portal hypertension. J. Hepatol. 2005;43:167–176.

16. Franchis R de. Revising consensus in portal hypertension: Report of the Baveno V consensus workshop on methodology of diagnosis and therapy in portal hypertension. J. Hepatol. 2010;53:762–768.

17. Franchis R de. Expanding consensus in portal hypertension: Report of the Baveno VI Consensus Workshop: Stratifying risk and individualizing care for portal hypertension. J. Hepatol. 2015;63:743–752.

18. Franchis R de, Bosch J, Garcia-Tsao G, *et al.* Baveno VII – Renewing consensus in portal hypertension. J. Hepatol. 2022;76:959–974.

19. Gerbes AL, Gülberg V, Sauerbruch T, *et al.* S3-Leitlinie „Aszites, spontan bakterielle Peritonitis, hepatorenales Syndrom". Z. Für Gastroenterol. 2011;49:749–779.

20. Gerbes AL, Labenz J, Appenrodt B, *et al.* [Updated S2k-Guideline "Complications of liver cirrhosis". German Society of Gastroenterology (DGVS)]. Z. Gastroenterol. 2019;57:e168.

21. Kamath PS, Kim WR, Advanced Liver Disease Study Group. The model for end-stage liver disease (MELD). Hepatol. Baltim. Md 2007;45:797–805.

22. Bettinger D, Sturm L, Pfaff L, *et al.* Refining prediction of survival after TIPS with the novel Freiburg index of post-TIPS survival. J. Hepatol. 2021;74:1362–1372.

23. Song J, Wang X, Yan Y, *et al.* MELD 3.0 Score for Predicting Survival in Patients with Cirrhosis After Transjugular Intrahepatic Portosystemic Shunt Creation. Dig. Dis. Sci. 2023;68:3185–3192.
24. Song J, Wang X, Yan Y, *et al.* MELD 3.0 Score for Predicting Survival in Patients with Cirrhosis After Transjugular Intrahepatic Portosystemic Shunt Creation. Dig. Dis. Sci. 2023;68:3185–3192.
25. Wiering L, Aigner A, Rosmalen M van, *et al.* Systematic Sex-Based Inequity in the MELD Score-Based Allocation System for Liver Transplantation in Germany. Transpl. Int. 2025;38:13844.
26. Fozouni L, Wang CW, Lai JC. Sex Differences in the Association Between Frailty and Sarcopenia in Patients With Cirrhosis. Clin. Transl. Gastroenterol. 2019;10:e00102.
27. Gödiker J, Schwind L, Jacob T, *et al.* Ultrasound-Defined Sarcopenia Independently Predicts Acute Decompensation in Advanced Chronic Liver Disease. J. Cachexia Sarcopenia Muscle 2024;15:2792–2802.
28. Delgado MG, Mertineit N, Bosch J, *et al.* Combination of Model for End-Stage Liver Disease (MELD) and Sarcopenia predicts mortality after transjugular intrahepatic portosystemic shunt (TIPS). Dig. Liver Dis. Off. J. Ital. Soc. Gastroenterol. Ital. Assoc. Study Liver 2024;56:1544–1550.
29. Schattenberg JM, Chalasani N, Alkhouri N. Artificial Intelligence Applications in Hepatology. Clin. Gastroenterol. Hepatol. 2023;21:2015–2025.
