# Supplementary material for: Comparative performance of ReMELD-Na, MELD 3.0 and established scores after TIPS for refractory ascites: A multicenter study
Source: JHEP Rep. 2026 Feb 21;8(5):101795. doi: 10.1016/j.jhepr.2026.101795 (PMC13091292; doi:10.1016/j.jhepr.2026.101795)
Supplement: Multimedia component 4 [file mmc4.pdf]

JHEP Reports

Comparative performance of ReMELD-Na, MELD 3.0 and established scores after TIPS for refractory ascites: A multicenter study

Authors

Markus Kimmann, Nancy Farouk, Dominik Bettinger, …, Michael Schultheiss, Jonel Trebicka, Michael Praktiknjo

Correspondence

michael.praktiknjo@ukmuenster.de (M. Praktiknjo).

Graphical abstract

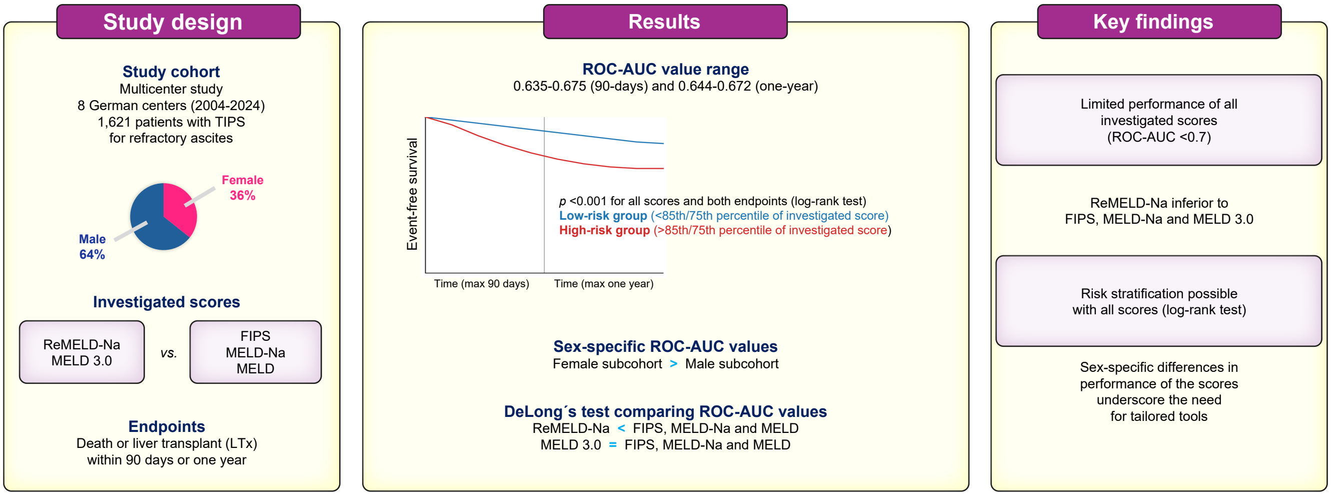

Highlights:

- All investigated prognostic scores demonstrated only modest accuracy for predicting outcomes after TIPS for refractory ascites.
- MELD 3.0 showed prognostic performance comparable to established post-TIPS models.
- ReMELD-Na performed worse than FIPS, MELD 3.0, and MELD-Na.
- Despite limited predictive capabilities, all scores effectively discriminated high-from low-risk patients.
- Sex-specific differences in performance underscore the need for tailored prognostic tools.

Impact and implications:

Accurate prediction of post-TIPS outcomes is essential to optimize management strategies for patients with cirrhosis and refractory ascites. In this large multicenter study, MELD 3.0 demonstrated prognostic performance comparable to established models, whereas ReMELD-Na – recently implemented for liver allocation in the Eurotransplant region – showed inferior predictive performance, raising concerns about its applicability in this setting. These results are particularly relevant as existing models may inadequately capture post-TIPS risk, especially in male patients. Collectively, the findings advocate for a cautious application of ReMELD-Na in clinical decision-making and emphasize the need to develop sex-sensitive, multidimensional prognostic tools to improve patient selection and surveillance.

https://doi.org/10.1016/j.jhepr.2026.101795
© 2026 The Author(s). Published by Elsevier B.V. on behalf of European Association for the Study of the Liver (EASL). This is an open access article under the CC BY license (http://creativecommons.org/licenses/by/4.0/).

Comparative performance of ReMELD-Na, MELD 3.0 and established scores after TIPS for refractory ascites: A multicenter study

Markus Kimmann[1], Nancy Farouk[1], Dominik Bettinger[2], Johannes Chang[3], Roman Kloeckner[4], Cristina Ripoll[5], Felix Piecha[6], Jassin Rashidi-Alavijeh[7], Juliana Stadtmann[1], Ahmad Shikh Mousa[1], Tony Bruns[8], Cornelius Engelmann[9], Benjamin Maasoumy[10], Christian Labenz[11], Lukas Sturm[2], Hauke Heinzow[12], Leon Louis Seifert[1], Michael Köhler[13], Max Masthoff[13], Johannes Kluwe[6], Alexander Zipprich[5], Christian Jansen[3], Carsten Meyer[14], Michael Schultheiss[2,15], Jonel Trebicka[1,16], Michael Praktiknjo[1,*], German Cirrhosis Study Group[†]

JHEP Reports **2026**. vol. 8 | 1–10

Check for updates

Background & Aims: ReMELD-Na and MELD 3.0 are newly introduced prognostic scores for liver graft allocation, but their ability to predict outcomes after transjugular intrahepatic portosystemic shunt (TIPS) for refractory ascites in Western populations remains uncertain. This study compared the prognostic performance of ReMELD-Na and MELD 3.0 with FIPS, MELD-Na, and MELD.

Methods: In this multicenter retrospective study, 1,621 patients with cirrhosis undergoing TIPS for refractory ascites at eight German centers (January 2004-June 2024) were analyzed. Outcomes were the composite of death or liver transplantation (LTx) within 90 days (primary endpoint) and one year (secondary endpoint) after TIPS. Prognostic performance was evaluated using the area under the receiver-operating characteristic curve (AUROC), including sex-stratified analyses, and compared using DeLong's test. High-risk groups (above the 85th percentile for the 90-day endpoint and the 75th percentile for the 1-year endpoint) were compared with non–high-risk groups using Kaplan–Meier analysis, scatter plots, and descriptive score-*vs*.-score spline smoothing.

Results: All scores showed limited predictive performance, with AUROC values ranging from 0.635 to 0.675 for the 90-day outcome and from 0.644 to 0.672 for the 1-year outcome. Female patients demonstrated higher AUROC values, reaching 0.714 for FIPS at 90 days. ReMELD-Na showed significantly lower AUROC values than FIPS, MELD 3.0, and MELD-Na. In contrast, MELD 3.0 demonstrated AUROC values comparable to those of the other scores. All models identified high-risk groups with increased rates of death and LTx.

Conclusions: After TIPS for refractory ascites, all scores exhibited limited prognostic performance, but adequately distinguished high- and low-risk patients. MELD 3.0 performed comparably to established models, while ReMELD-Na was inferior to FIPS, MELD 3.0, and MELD-Na. Higher AUROC values in women suggest sex-specific differences and highlight the need for sex-sensitive prognostic tools.

© 2026 The Author(s). Published by Elsevier B.V. on behalf of European Association for the Study of the Liver (EASL). This is an open access article under the CC BY license (http://creativecommons.org/licenses/by/4.0/).

Introduction

Cirrhosis presents a significant healthcare burden, contributing substantially to both morbidity and mortality.[1,2] Patients with cirrhosis are at an increased risk of hospitalization and a range of life-threatening complications.[3,4] Among these, refractory ascites and variceal bleeding demand effective management strategies. The implantation of a transjugular intrahepatic portosystemic shunt (TIPS) effectively reduces portal pressure and improves prognosis in selected patients.[5,6] However, adequate patient selection plays a major role in optimizing outcomes.[7,8] In this regard, accurate prediction of mortality risk in patients undergoing TIPS remains a critical aspect of clinical decision-making. High-risk subgroups may benefit from more intensive follow-up programs or *a priori* evaluation for liver transplantation (LTx). To address this need, several predictive scoring systems have been developed. The model for end-stage liver disease (MELD) score, initially developed to predict survival in patients with complications of portal hypertension undergoing elective TIPS placement, has become a cornerstone in the management of cirrhosis.[6] Over time, the MELD score was refined, with the inclusion of serum sodium concentration, as hyponatremia has been identified as an independent prognostic factor in cirrhosis.[9,10] In recognition of this, MELD-Na has been adopted for liver graft allocation in the United Network for Organ Sharing region since 2016.[11] The MELD 3.0 score

* Corresponding author. Address: Department of Internal Medicine B, University of Münster, Albert-Schweitzer-Campus 1 48149 Münster, Germany.
E-mail address: michael.praktiknjo@ukmuenster.de (M. Praktiknjo).
† German Cirrhosis Study Group available in appendix section.
https://doi.org/10.1016/j.jhepr.2026.101795

was introduced in 2021 and designed to further improve the prognostic capabilities of the scores especially in women by incorporating sex as an additional variable.[12] In 2025, the Eurotransplant region, which previously used the original MELD score, adopted the Refitted MELD-Na (ReMELD-Na) for liver graft allocation.[13]

The predictive capabilities of ReMELD-Na and MELD 3.0 in patients following TIPS insertion in large Western cohorts are unknown. Thus, the aim of this study was to evaluate the predictive performance of ReMELD-Na and MELD 3.0 for predicting outcomes (death or LTx) compared with the Freiburg Index of Post-TIPS Survival (FIPS), MELD-Na, and MELD in a large Western cohort of patients undergoing TIPS for refractory ascites, with particular attention to sex-specific performance.

Materials and methods

Study cohort

This multicenter retrospective study included 1,621 patients from the German Cirrhosis Study Group who underwent TIPS implantation for refractory ascites at eight German centers (Freiburg, Mainz, Hamburg, Hannover, Aachen, Bonn, Berlin, Münster) between January 2004 and June 2024. Due to data entry requirements, all parameters necessary for the calculation of the investigated scores were complete. Furthermore, patients aged below 18, with non-cirrhotic portal hypertension, Budd-Chiari syndrome and/or hepatocellular carcinoma were not permitted to be entered into the dataset. Cirrhosis was diagnosed by typical clinical, laboratory, ultrasound, and endoscopic findings, or confirmed histologically. Clinical and laboratory data were extracted from electronic medical records. All baseline data, particularly the values required to calculate the investigated scores, were assessed at the time of TIPS implantation in all centers. All decisions related to LTx allocation were determined using the MELD score, as all participating centers were located in Germany and part of the Eurotransplant region.

Ethics

Data collection and analysis within the framework of the German Cirrhosis Study Group were approved by the Ethics Committee of the University Medical Center Freiburg (approval No. EK 355/20; 22-1424-S1). Owing to the retrospective design, the requirement for written informed consent was waived. The study was conducted in accordance with the principles of the Declaration of Helsinki.

Procedure

The technique of TIPS implantation has been described previously.[14] A transjugular approach was used in all patients, with a puncture needle advanced into a hepatic vein. The portal vein was punctured under ultrasound guidance, followed by portography. The parenchymal tract was dilated, and a stent graft placed. Portal and central venous pressures were measured pre- and post-procedure to calculate the portosystemic pressure gradient. Indications for TIPS implantation followed the Baveno consensus and German clinical practice guidelines, which were valid at the time of TIPS placement.[15–20]

Outcomes and statistical analysis

SPSS (version 29, IBM, Armonk, NY, USA) and R (version 4.5.1, The R Foundation, Vienna, Austria) were used to analyze all data. Descriptive statistics were performed for all variables. Categorical variables are presented as counts and percentages. Continuous variables are presented as medians with interquartile ranges. Non-parametric testing (Chi-square test for categorical variables, Mann-Whitney *U* Test for continuous variables) was used to compare female and male patients. The detailed description of the calculation of each score can be found in the supplementary material (Supplementary Text 1).[12,13,21,22]

The primary endpoint was the combined event of death or LTx within 90 days after TIPS, defined as the occurrence of either death or LTx. The secondary endpoint was the combined event of death or LTx within 1-year after TIPS. Both events were considered as outcomes defining failure. Area under the receiver-operating characteristic curve (AUROC) values were calculated for the primary and secondary combined endpoint and each scoring system (ReMELD-Na, MELD 3.0, FIPS, MELD-Na and MELD). The AUROC values were compared with DeLong's test using R (R.app GUI 1.70 (7612 El Capitan build), S. Urbanek & H.-J. Bibiko, R Foundation for Statistical Computing). The ROC analyses were also stratified for male and female patients to evaluate the sex-specific performance of each score. Moreover, DeLong's test was performed comparing the AUROC values of ReMELD-Na and MELD 3.0 with the other scores. To explore potential confounders, we conducted additional sex-specific ROC analyses for factors that could influence the results, including age, etiology of cirrhosis, creatinine, and albumin levels. To stratify the patient cohorts for this analysis, we dichotomized the factors age, creatinine and albumin based on the median value in the entire cohort. To address the extended inclusion period and the evolving procedural and technical management practices, we performed a subgroup analysis (ROC analysis) using temporal stratification (early: 2004-2013; recent: 2014-2024) to compare the performance across these timeframes. Additionally, we conducted a separate analysis comparing bare-metal and covered stents, again employing ROC analysis. High-risk groups were defined as the patients above the 85th percentile (90-day endpoint) or 75th percentile (1-year endpoint) of each score using the values of each score in the entire study cohort to reflect event rates. The cut-off values to define high-risk groups were based on an approximation of the mortality for 90 days and 1 year. The positive and negative predictive values for each score were also calculated based on the respective high-risk and low-risk group. Kaplan-Meier and log-rank analyses were performed, comparing the high-risk group with the low-risk group for each score. Additionally, we further compared ReMELD-Na and MELD 3.0 with the other scores using scatter plots and descriptive score-*vs*.-score spline smooth analysis. Scatter plot analyses were performed to identify discordances between the previously defined high- and low-risk groups. Natural spline smoothing (descriptive score-*vs*.-score spline smooth) was performed to visualize potentially nonlinear score-to-score relationships within event and non-event groups in a descriptive manner. A detailed description can be found in the supplementary material (Supplementary text 2). Furthermore, a Fine–Gray

proportional hazards analysis was performed to investigate the competing events of death and LTx within 90 days and 1 year. Multiple subgroup analyses were conducted, and to maintain transparency, all corresponding *p* values are reported without adjustment for multiplicity. *P* values below 0.05 were considered statistically significant.

Results

General patient characteristics at baseline

This study included a total of 1,621 patients undergoing TIPS placement for refractory ascites. The median age was 59 (52-67) years, and the majority of patients were male (64.3%). The most common etiology of cirrhosis was alcohol-related liver disease (65.5 %). The median values were as follows: ReMELD-Na 14,[11–18] MELD 3.0 17,[13–21] FIPS 0.13 ((-0.48)-0.62), MELD 13[10–17] and MELD-Na 16[12–21] (Table 1). The average number of days at risk until the last follow-up contact among patients who survived the follow-up period was 77 days for the 90-day analysis (277 patients with follow-up of less than 90 days) and 267 days for the 1-year analysis (441 patients with follow-up of less than 1 year). The mortality rates were 16.3% for the 90-day and 25% for the 1-year endpoint. Rates for LTx were 1.4% for the 90-day and 3-9% for the 1-year endpoint. Consecutively, the rates for the combined endpoint (death/LTx) were 17.6% for the 90-day and 28.2% for the 1-year endpoint.

Primary outcome – 90-day combined event (death/LTx)

To assess the prognostic performance of ReMELD-Na, MELD 3.0, FIPS, MELD-Na, and MELD, a ROC analysis was performed in the entire cohort for the combined event (death/LTx) after TIPS insertion. The AUROC values in the entire cohort ranged between 0.639 and 0.675 (Table 2, Fig. S1). Sex-specific AUROC values for the 90-day endpoint were consistently higher in the female subgroup (ranging from 0.681 to 0.714) compared to the male subgroup (ranging from 0.614 to 0.652) (Table 2, Fig. S1).

Table 1. Baseline general characteristics of the entire study cohort as well as female and male patients separately.

Parameter	Entire cohort (N = 1,621)	Female (n = 579; 35.7%)	Male (n = 1,042; 64.3%)
**General and etiology**			
Age	59 (52–67)	60 (52–67)	59 (51–67)
Alcohol-related cirrhosis	1,061 (65.5%)	351 (58.9%)	720 (69.1%)
Viral hepatitis-related cirrhosis	171 (10.5 %)	56 (9.7%)	115 (11.0%)
Active alcohol-consumption	258 (17.2%)	104 (19.6%)	154 (14.8%)
Hepatic encephalopathy before TIPS	233 (14.2%)	91 (15.7%)	142 (13.6%)
ACLF before TIPS	380 (23.4%)	120 (20.7%)	260 (25.0%)
**Laboratory values**			
White blood cells (10^9/L)	6.3 (4.4–8.5)	6.1 (4.3–8.2)	6.4 (4.6–8.7)
Haemoglobin (g/L)	10 (8.8–11.7)	9.7 (8.7–11.0)	10.2 (8.9–12.0)
Platelets (10^9/L)	127 (86–180)	123 (85–179)	128 (87–181)
Creatinine (mg/dl)	1.26 (0.9–1.76)	1.2 (0.85–1.67)	1.30 (0.94–1.80)
Sodium (mmol/L)	135 (132–139)	136 (133–139)	135 (132–139)
Potassium (mmol/L)	4.2 (3.8–4.7)	4.2 (3.8–4.6)	4.3 (3.8–4.7)
Bilirubin (mg/dl)	1.3 (0.8–1.9)	1.2 (0.7–1.8)	1.3 (0.8–1.9)
Albumin (g/L)	29 (25–33)	30 (26–34)	28 (24–33)
INR	1.25 (1.12–1.43)	1.23 (1.12–1.40)	1.27 (1.11–1.46)
**Hemodynamics & TIPS**			
PSG before TIPS	19 (15–22)	19 (15–23)	18 (15–22)
PSG after TIPS	8 (6–10)	8 (6–10)	8 (5.5–10)
Relative PSG reduction	52.6%	52.3%	52.6%
Bare-metal/covered stent	187/1,434 (11.5/88.5%)	57/522 (10%/90%)	130/912 (12.5%/87.5%)
**Scores**			
ReMELD-Na	14 (11–18)	14 (10–17)	15 (11–18)
MELD 3.0	17 (13–21)	16 (13–21)	17 (13–21)
FIPS	0.13 ((–0.48)–0.62)	0.01 ((–0.62)–0.54)	0.19 ((–0.38)–0.65)
MELD	13 (10–17)	13 (10–16)	14 (11–17)
MELD-Na	16 (12–21)	15 (11–20)	16 (12–21)
**Outcomes**			
90-day mortality	265 (16.3%)	98 (16.9%)	167 (16.0%)
One-year mortality	405 (25%)	139 (24%)	266 (25.5%)
Liver transplantation within 90 days post TIPS	23 (1.4%)	9 (1.6%)	14 (1.3%)
Liver transplantation within one year post TIPS	63 (3.9%)	18 (3.1%)	45 (4.3%)
90-day combined event (death/LTx)	285 (17.6%)	105 (18.1%)	180 (17.3%)
One-year combined event (death/LTx)	457 (28.2%)	154 (26.6%)	303 (29.1%)

Categorical values are represented as n, (% of available data). Continuous values are represented as median (IQR). Median time to death/LTx/loss to follow-up was 77 days (for the 90-day analysis) and 267 days (for the 1-year analysis). Complete 90-day follow-up: 82.91%. Complete 1-year follow-up: 72.79%. Availability of parameters: 100% – age, alcohol-related cirrhosis, viral hepatitis-related cirrhosis, hepatic encephalopathy before TIPS, ACLF before TIPS, hemoglobin, platelets, creatinine, sodium, potassium, bilirubin, albumin, INR, Bare-metal/covered stent, ReMELD-Na, MELD 3.0, FIPS, MELD, MELD-Na; 97.9% – Relative PSG reduction; 97.5% – PSG after TIPS; 96.5% – PSG before TIPS; 91.12% – active alcohol-consumption; 75.88% - white blood cells; 91.92% – hemoglobin.

ACLF, acute-on-chronic liver failure; AUROC, area under the receiver-operating characteristic curve; FIPS, Freiburg Index of post-TIPS survival; INR, international normalized ratio; LTx, liver transplant(ation); MELD, model of end-stage liver disease; PSG, portosystemic pressure gradient; ReMELD-Na, Refitted MELD-Na; TIPS, transjugular intrahepatic portosystemic shunt.

Table 2. Results of the ROC analysis in the entire study cohort with AUROC values displayed for ReMELD-Na, MELD 3.0, FIPS, MELD and MELD-Na for all patients as well as female and male patients separately.

Entire cohort (90-day combined event (death/LTx))	Overall (N = 1,621)	Female (n = 579)	Male (n = 1,042)
ReMELD-Na	0.636 (0.601–0.670)	0.681 (0.626–0.737)	0.610 (0.566–0.653)
MELD 3.0	0.654 (0.620–0.689)	0.698 (0.643–0.754)	0.629 (0.585–0.673)
FIPS	0.675 (0.641–0.708)	0.714 (0.661–0.768)	0.652 (0.609–0.694)
MELD	0.639 (0.603–0.675)	0.682 (0.624–0.741)	0.614 (0.569–0.660)
MELD-Na	0.655 (0.621–0.690)	0.700 (0.645–0.755)	0.630 (0.586–0.674)

Outcome: 90-day combined event (death/LTx).
AUROC, area under the receiver-operating characteristic curve; FIPS, Freiburg Index of post-TIPS survival; LTx, liver transplant(ation); MELD, model of end-stage liver disease; ReMELD-Na, Refitted MELD-Na.

Table 3. Comparison of the AUROC values for the different models.

Compared score	*p* (90-day)	AUROC value difference (90-day)	*p* (1-year)	AUROC value difference (1-year)
**Training cohort - Reference ReMELD-Na**				
All				
MELD 3.0	0.019*	-0.019	0.318°	-0.006
FIPS	0.001*	-0.039	0.011*	-0.025
MELD	0.720°	-0.003	0.679°	0.003
MELD-Na	0.018*	-0.019	0.400°	-0.006
Female				
MELD 3.0	0.209°	-0.017	0.414°	-0.01
FIPS	0.066°	-0.033	0.451°	-0.013
MELD	0.928°	-0.001	0.454°	0.009
MELD-Na	0.184°	-0.019	0.206°	-0.015
Male				
MELD 3.0	0.041*	-0.019	0.420°	-0.006
FIPS	0.006*	-0.042	0.009*	-0.031
MELD	0.705°	-0.004	0.982°	0
MELD-Na	0.046*	-0.02	0.963°	0
**Training cohort - Reference MELD 3.0**				
Overall				
ReMELD-Na	0.019*	0.019	0.318°	0.006
FIPS	0.153°	-0.02	0.123°	-0.018
MELD	0.158°	0.016	0.290°	0.009
MELD-Na	0.872°	-0.001	0.784°	0.001
Female				
ReMELD-Na	0.209°	0.017	0.414°	0.01
FIPS	0.480°	-0.016	0.873°	-0.003
MELD	0.381°	0.016	0.222°	0.019
MELD-Na	0.728°	-0.002	0.280°	-0.006
Male				
ReMELD-Na	0.041*	0.019	0.420°	0.006
FIPS	0.203°	-0.023	0.073°	-0.025
MELD	0.279°	0.015	0.595°	0.006
MELD-Na	0.892°	-0.001	0.186°	0.006

(A) Comparison of the AUROC values of ReMELD-Na with MELD 3.0, FIPS, MELD and MELD-Na in the entire study cohort (overall, female and male patients displayed separately). (B) Comparison of the AUROC values of MELD 3.0 with ReMELD-Na, FIPS, MELD and MELD-Na in the entire study cohort. Outcome: 90-day combined event (death/LTx) and 1-year combined event (death/LTx). AUROC value differences displayed for the 90-day and 1-year combined event (death/LTx) outcome separately. *p* values (DeLong's test) displayed for the 90-day and 1-year combined event (death/LTx) outcome for each score as well as all, female and male patients separately. Levels of significance: **p* <0.05 (DeLong's test); °*p* >0.05 (DeLong's test). AUROC, area under the receiver-operating characteristic curve; FIPS, Freiburg Index of post-TIPS survival; LTx, liver transplant(ation); MELD, model of end-stage liver disease; ReMELD-Na, Refitted MELD-Na.

In the entire cohort, DeLong's test showed significantly lower AUROC values of ReMELD-Na compared to MELD 3.0 (p = 0.019), MELD-Na (p = 0.018) and FIPS (p = 0.001), while MELD's AUROC value was comparable (p = 0.720) (Table 3A). For female patients, ReMELD-Na was only numerically inferior to FIPS (p = 0.066), while for male patients, MELD 3.0 (p = 0.041), MELD-Na (p = 0.046) and FIPS (p = 0.006) were all superior compared to ReMELD-Na in terms of AUROC values (Table 3A).

DeLong's test comparing MELD 3.0 with the other scores showed a significantly lower AUROC for ReMELD-Na (p = 0.019). The other scores demonstrated comparable performance for predicting 90-day death/LTx–free survival. In female patients, the AUROC of MELD 3.0 was comparable to that of the other scores, whereas in male patients ReMELD-Na was inferior to MELD 3.0 (p = 0.041) (Table 3B).

Secondary outcome – 1-year combined event (death/LTx)

In the entire cohort, the AUROC values for the 1-year combined event (death/LTx) ranged between 0.644 and 0.672 (Fig. S2, Table 4). However, the sex-specific AUROC values for 1-year LTx-free survival were slightly higher for females compared to men and ranged from 0.658 to 0.682 for females and from 0.635 to 0.666 for males (Fig. S2, Table 4).

Table 4. Results of the ROC analysis in the entire study cohort with AUROC values displayed for ReMELD-Na, MELD 3.0, FIPS, MELD and MELD-Na for all patients, as well as female and male patients separately.

Entire cohort (1-year combined event (death/LTx)	Overall (N = 1,621)	Female (n = 579)	Male (n= 1,042)
ReMELD-Na	0.647 (0.618–0.676)	0.667 (0.619–0.715)	0.635 (0.598–0.671)
MELD 3.0	0.653 (0.624–0.682)	0.677 (0.628–0.725)	0.641 (0.605–0.677)
FIPS	0.672 (0.643–0.700)	0.680 (0.632–0.728)	0.666 (0.630–0.703)
MELD	0.644 (0.614–0.674)	0.658 (0.608–0.708)	0.635 (0.598–0.672)
MELD-Na	0.652 (0.623–0.682)	0.682 (0.634–0.730)	0.635 (0.599–0.672)

Outcome: 1-year combined event (death/LTx).
AUROC, area under the receiver-operating characteristic curve; FIPS, Freiburg Index of post-TIPS survival; LTx, liver transplant(ation); MELD, model of end-stage liver disease; ReMELD-Na, Refitted MELD-Na.

DeLong's test comparing ReMELD-Na with the other scores showed a significantly lower AUROC for ReMELD-Na compared with FIPS (p = 0.011). The other scores showed comparable performance to ReMELD-Na. No significant differences between ReMELD-Na and the other scores were observed in the female subcohort. In contrast, in the male subcohort, ReMELD-Na was again inferior to FIPS (p = 0.009) (Table 3A).

When comparing the AUROC value of MELD 3.0 with the other scores, there were no significant differences in the entire cohort or in the male and female subcohorts. However, in male patients, the numerically higher AUROC of FIPS narrowly failed to reach statistical significance (p = 0.073).

Further stratification of the sex-specific ROC analysis

Sex-specific ROC analyses were conducted for the 90-day (Table S1) and 1-year (Table S2) endpoints, further stratified by alcohol-related cirrhosis status, and dichotomized at the median values of age (59 years), creatinine (1.26 mg/dl), and albumin (29 g/L). For the 90-day endpoint, the female subcohort showed higher AUROC values than the male subcohort in patients without alcohol-related cirrhosis, those aged >59 years, and those with creatinine <1.26 mg/dl or albumin <29 g/L. For the 1-year endpoint, differences were smaller, but AUROC values remained higher in females aged >59 years and in those with albumin <29 g/L.

Score performance stratified by inclusion time (2004-2013 and 2014-2024)

To account for the 20-year inclusion period and evaluate potential temporal differences in score performance, we conducted subgroup ROC analyses for patients who received TIPS between 2004 to 2013 or 2014 to 2024. For the 90-day endpoint, AUROC values were lower in the 2004–2013 subcohort (0.588–0.641) than in the 2014–2024 subcohort (0.678–0.700). For the 1-year endpoint, differences were less pronounced, with AUROC values of 0.624–0.665 in the 2004–2013 subcohort and 0.656–0.671 in the 2014–2024 subcohort (Tables S3 and S4).

Score performance stratified by stent type (bare metal and covered stents)

Furthermore, we conducted subgroup ROC analyses for patients who received either a bare-metal stent (n = 187) or a covered stent (n = 1,434). For the 90-day endpoint, AUROC values ranged from 0.627 to 0.710 in the bare-metal stent subgroup and from 0.641 to 0.663 in the covered-stent subgroup. For the 1-year endpoint, AUROC values ranged from 0.658 to 0.698 in the bare-metal stent subgroup and from 0.646 to 0.665 in the covered-stent subgroup (Tables S5 and S6).

High- and low-risk group stratification

The entire cohort was stratified into high- and low-risk groups based on the 85th (for the 90-day combined endpoint) or 75th (for the 1-year combined endpoint) percentile of each score. Threshold values (85th percentile) were 20 for ReMELD-Na, 23 for MELD 3.0, 0.92 for FIPS, 23 for MELD-Na, and 19 for MELD for the 90-day combined endpoint. For the primary outcome, log-rank tests demonstrated significantly worse survival in the high-risk groups compared to their respective low-risk groups for all scores (all p ≤0.001) (Fig. 1). Threshold values (75th percentile) were 18 for ReMELD-Na, 21 for MELD 3.0, 0.62 for FIPS, 21 for MELD-Na, and 17 for MELD for the 1-year combined endpoint. Similarly, for the secondary outcome, all scores effectively stratified patients, with high-risk groups showing significantly worse survival than low-risk groups (all p <0.001) (Fig. S3).

Additionally, we performed a Fine-Gray proportional hazard analysis by using the same stratification of the cohort and analyzing the 90-day and 1-year cumulative incidence of death under the competing risk of LTx. Similarly, all analyses showed significantly higher cumulative incidences of death in the high-risk group for each score and both endpoints (all p <0.001) (Fig. S4, Table S7).

Subsequent analyses of the high- and low-risk groups were performed by calculating the negative and positive predictive values (NPV and PPV) for each cohort, as well as for male and female subgroups within each cohort. For the primary outcome, NPVs ranged from 84.4% to 85.6% in the entire cohort and were mostly similar in the female and male subcohorts (84.8% to 86% and 84.2% to 85.4%, respectively). PPVs were low, ranging from 28.3% to 33.1%, with slightly higher values in female patients (37.6% to 41.2%) and lower values in male patients (24.4% to 30.4%) (Table S8). For the secondary outcome, NPVs were lower, ranging from 76.0% to 77.2%, and were only slightly higher in the female subcohort (77.9% to 79.9%) compared to the male subcohort (74.8% to 76.4%). PPVs remained low but were slightly higher than for the primary outcome in the entire cohort (39.9% to 43.6%) as well as in the female (42.9% to 45.6%) and male (38.7% to 42.9%) subcohorts (Table S9).

Scatter plots were constructed to assess discordances between ReMELD-Na and other scoring systems. Discordance rates were 5.6%/9.8% for MELD 3.0, 5.9%/9.5% for MELD-Na, 7.3%/8.4% for MELD, and 11.4%/13.6% for FIPS for the

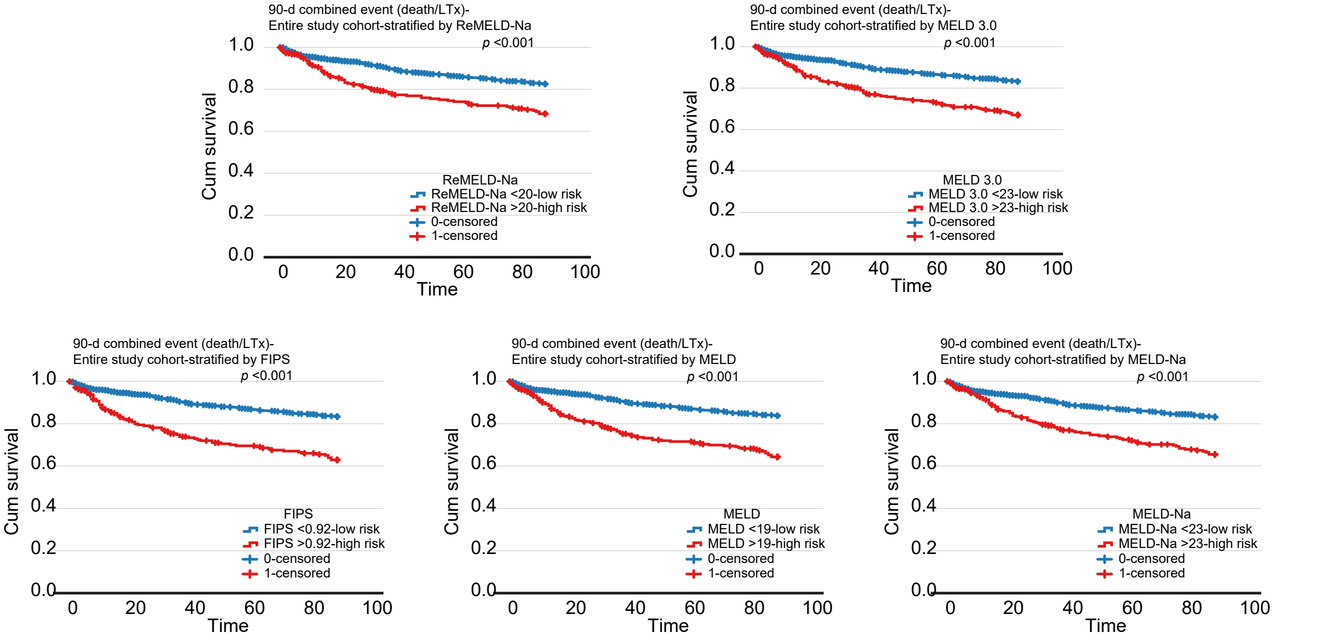

Fig. 1. Kaplan-Meier curves showing 90-day combined event (death/LTx)-free survival for high-*vs*. low-risk groups, stratified by the 85th percentile of each scoring system. Cut-off values used to define high-risk groups were: ReMELD-Na = 20, MELD 3.0 = 23, FIPS = 0.92, MELD = 19, and MELD-Na = 23. Level of significance for each Kaplan-Meier curve: *p* <0.001 (log-rank test).

90-day and 1-year endpoint, respectively (Figs 2A and S4A). When evaluating discordances between MELD 3.0 and the remaining scores, rates were 8.8%/12.6% for MELD, 3.6%/4.7% for MELD-Na, 5.6%/9.1% for ReMELD-Na and 12.4%/16.1% for FIPS for the 90-day and 1-year endpoint, respectively (Figs 2B and S4B).

Descriptive score-*vs*.-score spline smooth analyses demonstrated that ReMELD-Na curves closely aligned with those of MELD, MELD-Na, and MELD for short-term risk estimation. In contrast, when comparing FIPS with ReMELD-Na, the curves diverged slightly, particularly in the intermediate disease severity range, indicating a superior predictive capability of FIPS (Figs 3A and S5A). Similarly, MELD 3.0 curves corresponded closely to MELD, MELD-Na, and ReMELD-Na. However, when comparing MELD 3.0 with FIPS, the curves also diverged. For the 90-day endpoint, differences were most pronounced at low to intermediate scores, whereas for the 1-year endpoint, the divergence occurred relatively uniformly across all scores (Figs 3B and S5B).

Discussion

This study sought to evaluate the predictive performance of ReMELD-Na and MELD 3.0 for a combined outcome (death or LTx) in patients with cirrhosis undergoing elective TIPS placement for refractory ascites and compare it to other established scoring systems (FIPS, MELD and MELD-Na).

Our analysis revealed that all analyzed scoring systems showed limited performance after TIPS insertion for refractory ascites, with AUROC values below 0.7 when including both male and female patients. However, we were able to identify notable differences in performance by stratifying the cohort into subcohorts. On the one hand, ReMELD-Na demonstrated significantly lower AUROC values compared to FIPS in all analyses and for all outcomes when including all patients of the respective cohort as well as male patients only. Additionally, ReMELD-Na was inferior to MELD 3.0 and MELD-Na for the 90-day but not the 1-year endpoint. On the other hand, the predictive performance of MELD 3.0 indexed by AUROC values was mostly comparable to the other scores in most analyses.

This important finding raises the question of whether the newly introduced ReMELD-Na-based liver graft allocation in the Eurotransplant region may be suboptimal for this group of patients receiving TIPS for refractory ascites, because it showed inferiority to FIPS, MELD and MELD-Na. Still, further studies on waitlist mortality for these patients are warranted.

Interestingly, a large Chinese study found that MELD 3.0 was superior to FIPS, MELD and MELD-Na in predicting mortality 3 months, 6 months, 1 year and 2 years after TIPS insertion.[23] However, the divergence to our study's results might be explained due to differences in patient characteristics between our studies, as well as other potential regional factors that might influence the performance of each score. For example, the indication for TIPS was variceal bleeding in 95.2% of patients in the Chinese study, while our study focuses on patients receiving elective TIPS for refractory ascites. Viral hepatitis-related cirrhosis was the most common etiology in the Chinese study (62.6% hepatitis B and 4.2% hepatitis C), while being a minor etiology (10.5%) in our study, which mainly consists of patients with alcohol-related cirrhosis (65.5%). Patients included in the Chinese study had a median age of 51.5 years, while our patients were older with a median age of 59 years at the time of TIPS insertion. Furthermore, significantly lower values of the investigated scores could be observed in the Chinese study with median values of 10.9 for MELD 3.0, -1.1 for FIPS, 10.6 for MELD 10.6 and 11 for MELD-Na. Unfortunately, the performance of ReMELD-Na was not

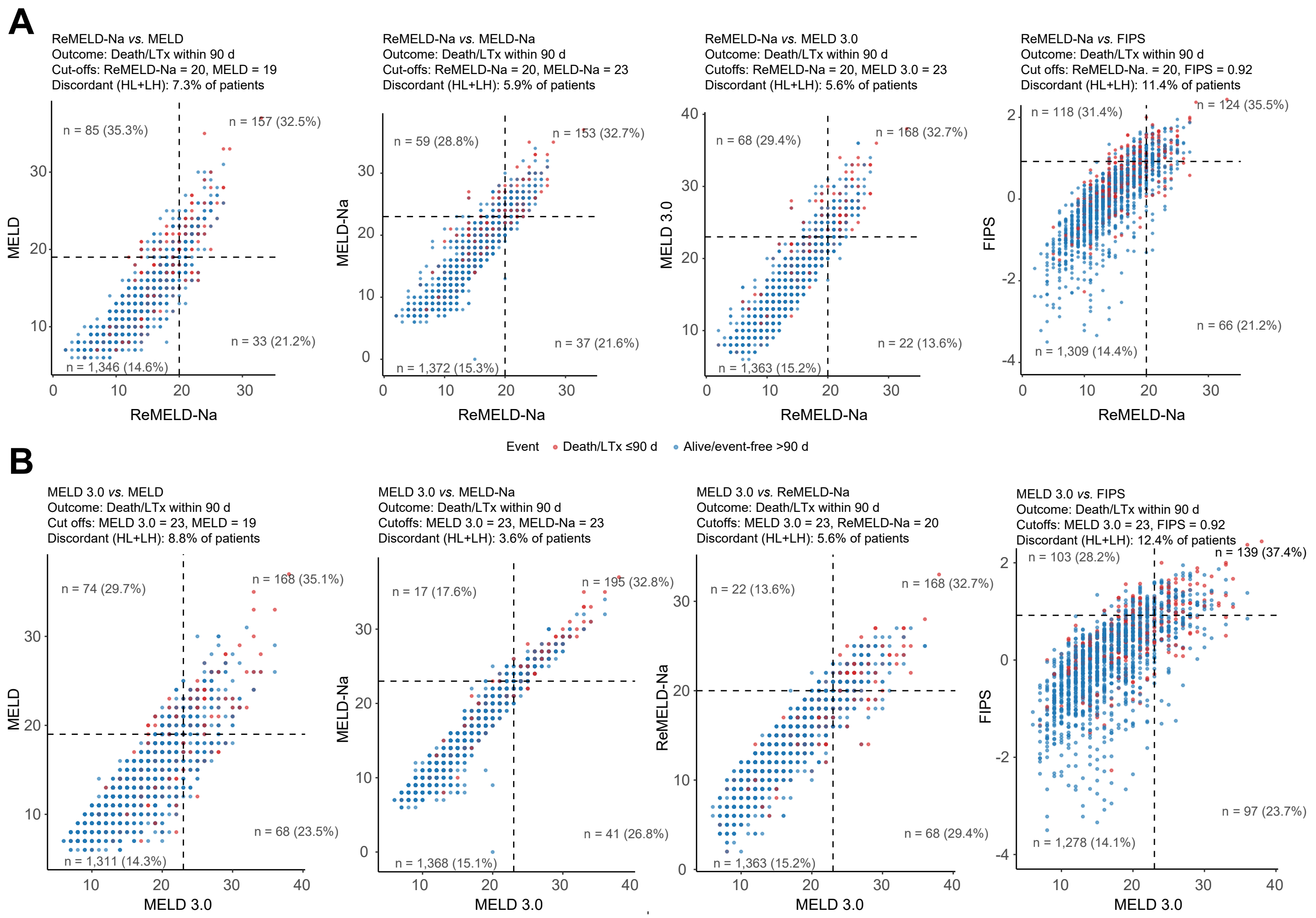

Fig. 2. Scatter plots of patients who reached the combined endpoint (death/LTx) before censoring (orange) and those who did not (blue) within 90 days. (A) Cube positions reflect ReMELD-Na (x-axis) and MELD, MELD-Na, MELD 3.0, or FIPS (y-axis). (B) Cube positions reflect MELD 3.0 (x-axis) and MELD, MELD-Na, ReMELD-Na, or FIPS (y-axis). Based on high-*vs.* low-risk thresholds (ReMELD-Na = 20, MELD 3.0 = 23, FIPS = 0.92, MELD = 19, MELD-Na = 23), a 2 × 2 grid was defined: lower left = low/low-risk, lower right = high/Low-risk, upper left = low/high-risk, upper right = high/high-risk. Lower right + upper left show discordant patients. Each quadrant shows n (patients) and % (events). FIPS, Freiburg Index of post-TIPS survival; LTx, liver transplant(ation); MELD, model of end-stage liver disease; ReMELD-Na, Refitted MELD-Na.

assessed in the Chinese study. Overall, due to the significant differences in terms of patient characteristics and study design, the comparability of both studies is limited.

We want to highlight that sex-specific analyses further emphasized the variability in the predictive capacity of the analyzed scoring systems. For instance, each score demonstrated higher AUROC values in women compared to men. This finding raised our interest, especially because only MELD 3.0 incorporates sex as a variable. This appears counterintuitive, because previous studies demonstrated that female patients are typically disadvantaged by the traditional scoring systems.[24,25] Our data suggest that the opposite could be true in patients receiving TIPS for refractory ascites. Overall, these findings suggest that sex-specific factors may influence the performance of predictive scores and warrant further investigation. Interestingly, our additionally performed ROC analysis (stratified by alcohol-related cirrhosis status, age, creatinine and albumin) showed higher AUROC values in the female subcohort in case of non-alcohol-related cirrhosis, age >59 years, creatinine <1.26 mg/dl and albumin <29 g/L, which is in line with a higher risk for sarcopenia. Thus, one might assume that the better performance of all scores in the female subcohort of patients might be at least partially explained by sarcopenia. Furthermore, the observed differences might be attributed to physiological or hormonal factors that are not fully accounted for in these models, such as sarcopenia.[26,27] It is clear that the disparity between sexes observed in our study calls for the development of more tailored scoring systems that account for sex differences, potentially improving predictive performance and thus patient care.

Our analysis reveals an improvement in the short-term predictive performance of prognostic models. We observed consistently higher 90-day AUROC values within the more recent 2014–2024 subcohort, which clearly points to enhanced predictive accuracy for contemporary patients. This positive development possibly reflects the ongoing advancements in clinical management strategies and potentially more consistent patient characteristics over time. In contrast, the differences between cohorts regarding the 1-year endpoint were less pronounced. This suggests that long-term outcomes show greater inherent variability and are less influenced by these temporal shifts in care.

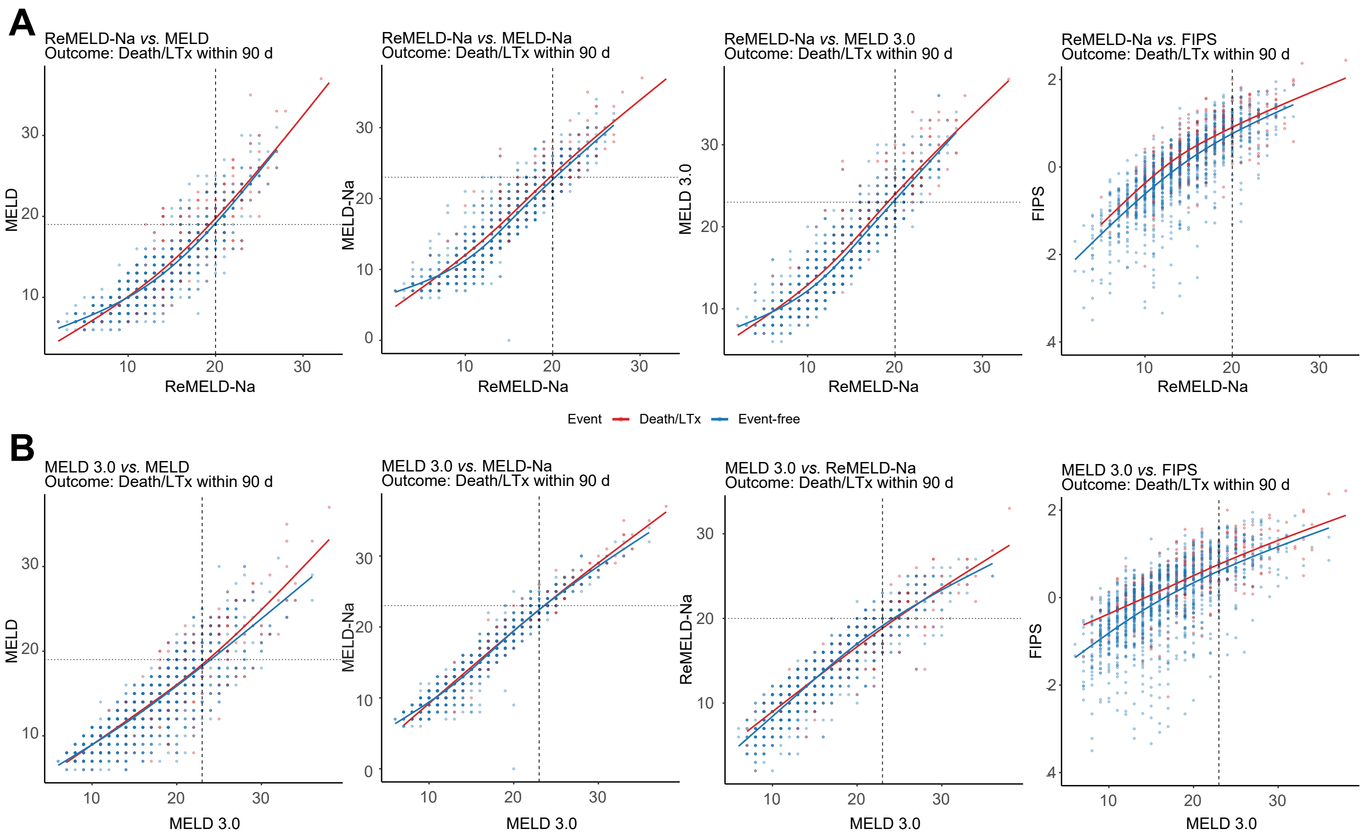

Fig. 3. Descriptive score-*vs.*-score spline smooth analyses of patients who reached the combined endpoint (death/LTx) before censoring or loss-to follow-up (orange) and those who did not (blue) within 90 days, depicting the association between prognostic scores and events. (A) Cube positions reflect ReMELD-Na (x-axis) and MELD, MELD-Na, MELD 3.0, or FIPS (y-axis). (B) Cube positions reflect MELD 3.0 (x-axis) and MELD, MELD-Na, ReMELD-Na, or FIPS (y-axis). FIPS, Freiburg Index of post-TIPS survival; LTx, liver transplant(ation); MELD, model of end-stage liver disease; ReMELD-Na, Refitted MELD-Na.

Furthermore, our comparison of stent types illuminated distinct differences concerning outcome variability. Bare metal stents, for example, presented wider confidence intervals when compared to covered stents. This could be explained either by greater inherent outcome heterogeneity or by smaller sample sizes within this particular subgroup. Importantly, while overall AUROC values themselves differed only modestly, ReMELD-Na and MELD achieved a slightly higher discrimination for the covered stent subgroup, whereas MELD 3.0, FIPS, and MELD-Na performed better among patients who received bare metal stents. However, it is essential to note that currently, only covered stent types are employed for TIPS placement. Given the rather modest differences observed between bare metal and covered stents, alongside the results from the entire cohort, the authors do not believe that specific scores for specific stent types should necessarily be recommended.

When considering risk stratification, dividing cohorts into high- and low-risk groups based on the 85th or 75th percentile for each score revealed significant differences in outcomes between high- and low-risk groups, which supports the potential clinical relevance of each model in identifying high-risk patients. The log-rank tests confirmed that the event rate was significantly higher in the high-risk groups for all the scoring systems at both 90 days and 1 year, underlining the potential utility of all these models in identifying patients who may benefit from more intensive follow-up programs or *a priori* evaluation for LTx. Overall, the scatter plots showed that discordance between all scores was mostly low, especially between MELD variants. Likewise, the descriptive score-*vs.*-score spline smooth analyses demonstrated that overall discriminatory capacity remained broadly comparable across all models.

While our study provides valuable insights into the predictive capabilities of these scores and for the first time evaluated ReMELD-Na and MELD 3.0 in a large Western cohort of patients following TIPS insertion for refractory ascites, there are relevant limitations to consider. The main limitation of our study is its retrospective design. Although patients were enrolled consecutively, both the data assessments and the statistical analysis were conducted retrospectively. Our study is a multicenter study including patients from eight distinct centers with independent practice patterns, numbers of TIPS placements per year and standard operation procedures, which offers substantial internal heterogeneity. However, it must be acknowledged that only patients from Germany were included, which may limit the generalizability of our results to other, particularly non-Western, cohorts. It must be recognized that the relatively weak performance of MELD-based scores, which is known for well-selected TIPS candidates, is likely due to the generally low MELD values in our cohort, which may limit the ability of these MELD variants to capture short-term risk in this

type of patient population. Nevertheless, all scores were still able to distinguish between high- and low-risk groups. Furthermore, our study partially includes patients from the large Western cohort in which the FIPS score was originally derived and validated,[22] which may introduce a bias favoring FIPS.

Overall, our findings indicate that all currently established models for outcome prediction in patients with cirrhosis undergoing TIPS insertion still exhibit limited performance, with AUROC values below 0.7 for each score, highlighting the need to use the scores with caution and the need for improvement. Although these scores were able to sufficiently identify high-risk groups of patients who may benefit from more intensive follow-up programs or *a priori* evaluation for LTx before TIPS insertion, there remains a need for better-tailored risk stratification models. Improvement may be achievable by integrating additional clinical variables such as sarcopenia, frailty, artificial intelligence-based metrics and developing entirely new models that more comprehensively consider individual patient characteristics.[28,29] Incorporating such factors could enhance the predictive performance of these models and support clinical decision-making for optimal patient selection for TIPS insertion. Additionally, the observed sex-specific differences also warrant further research and investigation. Importantly, our study showed that MELD 3.0 demonstrated comparable performance to established scores (FIPS, MELD, and MELD-Na) in predicting 90-day and 1-year death or LTx outcomes in patients undergoing TIPS for refractory ascites. Notably, the ReMELD-Na score, which recently replaced MELD for liver allocation in the Eurotransplant region, was inferior to FIPS in most analyses and to MELD 3.0 and MELD-Na in some analyses. In conclusion, the prognostic performance of ReMELD-Na for patients undergoing TIPS placement for refractory ascites is limited.

Affiliations

[1]*University Hospital Münster, Medical Clinic B, Münster, Germany;* [2]*University Hospital Freiburg, Clinic for Internal Medicine II, Freiburg, Germany;* [3]*University Medical Centre Bonn, Department of Internal Medicine I, Bonn, Germany;* [4]*University Hospital Schleswig-Holstein – Lübeck, Department of Diagnostic and Interventional Radiology, Lübeck, Germany;* [5]*Internal Medicine IV, Department for Gastroenterology, Hepatology, Interdisciplinary Endoscopy and Infectious Diseases, Jena University Hospital, Germany;* [6]*University Medical Centre Hamburg-Eppendorf, I. Medical Clinic and Polyclinic, Hamburg, Germany;* [7]*University Hospital Essen, Clinic for Gastroenterology, Hepatology and Transplantation Medicine, Essen, Germany;* [8]*University Hospital RWTH Aachen, Clinic for Gastroenterology, Metabolic Diseases and Internal Intensive Care Medicine (Medical Clinic III), Aachen, Germany;* [9]*University Medicine Charite Berlin, Division of Hepatology and Gastroenterology, Berlin, Germany;* [10]*Hannover Medical School, Department of Gastroenterology, Hepatology, Infectious Diseases and Endocrinology, Germany;* [11]*University Medicine Mainz, Medical Clinic and Polyclinic, Mainz, Germany;* [12]*Krankenhaus der Barmherzige Brüder Trier, Internal Medicine I, Trier, Germany;* [13]*University Hospital Münster, Clinic for Radiology, Münster, Germany;* [14]*University Hospital Bonn, Clinic for Diagnostic and Interventional Radiology, Germany;* [15]*Berta-Ottenstein-Programme, Faculty of Medicine, University of Freiburg, Germany;* [16]*European Foundation for the Study of Chronic Liver Failure, Barcelona, Spain*

Abbreviations

AUROC, area under the receiver-operating characteristic curve; FIPS, Freiburg Index of post-TIPS survival; LTx, liver transplant(ation); MELD, model of end-stage liver disease; NPV, negative predictive value; PPV, positive predictive value; ReMELD-Na, Refitted MELD-Na; ROC, receiver-operating characteristics; TIPS, transjugular intrahepatic portosystemic shunt.

Financial support

JC: Research Grants: Advanced Clinician Scientist Programme (ACCENT funding code 01EO2107) sponsored by the German Federal Ministry of Education and Research (BMBF), Ernst und Berta Grimmka Foundation No. 6/23.

Conflicts of interest

Dominik Bettinger has received grants from German Research Foundation and Schwiete Foundation, honoraria from W.L. Gore & Associates and Falk Foundation and support for attending meetings/travel from Abbvie. Jassin Rashidi-Alavijeh has received consulting fees from Ipsen, honoraria from Tillotts and support for attending meeting/travel from AbbVie, Ipsen and Gilead. Tony Bruns has received consulting fees from Intercept Pharma, Grifols, Sobi Deutschland, Gilead and SmartDyeLivery, honoraria from Falk Foundation, CSL Behring, Merck, Gilead, Intercept, Advanz Pharma and W.L. Gore & Associates. Benjamin Maasoumy has received grants from Roche, Ewimed and Altona Diagnostics, consulting fees from Ewimed, Norgine, Luvos, Ipsen and Roche, honoraria from AbbVie, AstraZeneca, W.L. Gore & Associates, Gilead, Roche, Norgine, Merz and Falk and support for attending meetings/travel from Gilead, AbbVie and Falk and holds stocks/stock options from AbiVax and Biontech. Christian Labenz has received grants from Merz Therapeutics and Norgine, consulting fees from Norgine Alfasigma, Ewimed and Boehringer Ingelheim, honoraria from Norgine, Allergosan, AbbVie, Merz Therapeutics, Intercept, Gilead Sciences, Falk Foundation and Ipsen, support for attending meetings/travel from Gilead Sciences and participated on a Data Safety Monitoring Board or Advisory Board for Boehringer Ingelheim, Alfasigma, Norgine and Ipsen. Hauke Heinzow has received honoraria from Gilead, Ipsen and AbbVie, support for attending meetings/travel from Alphasigma, Gilead and AbbVie and participated on a Data Safety Monitoring Board or Advisory Board for Johnson&Johnson and AbbVie and holds stocks/stock options from Bayer. Johannes Kluwe has received a grant from the German Federal Ministry of Education and Research. Michael Schultheiss has received honoraria from Falk Foundation, Bentley InnoMed and W.L. Gore & Associates. Jonel Trebicka has been supported by the German Research Foundation (DFG), German Federal Ministry of Education and Research, Hessian Ministry of Higher Education, Research and the Arts (HMWK) and European Union – Horizon 2020 (MICROB-PREDICT ID 825694, DECISION ID 847949, GALAXY ID 668031, LIVERHOPE ID 731875, IHMCSA ID964590) and has received consulting fees and honoraria from AstraZeneca, W.L. Gore & Associates, Boehringer Ingelheim, Falk, Grifols, Genfit, CSL Behring and Versantis, Michael Praktiknjo has been supported by the German Research Foundation (DFG), BONFOR and the Ernst-und Berta Grimmke Foundation and has received consulting fees, honoraria and support for attending meetings/travel from speaking fees from W.L. Gore and Associates, Orphalan, Gilead, Falk, Univar, Ipsen, Roche, AstraZeneca, Boston Scientific and MicroTec.

Please refer to the accompanying ICMJE disclosure forms for further details.

Authors' contributions

All authors approved the final version of the article, including the authorship list. Conceptualization: Markus Kimmann, Michael Praktiknjo. Methodology: Markus Kimmann, Dominik Bettinger, Michael Praktiknjo. Data curation: Markus Kimmann, Nancy Farouk, Johannes Chang, Dominik Bettinger, Michael Praktiknjo. Investigation: Markus Kimmann, Nancy Farouk, Dominik Bettinger, Johannes Chang, Roman Kloeckner, Cristina Ripoll, Felix Piecha, Jassin Rashidi-Alavijeh, Juliana Gödiker, Ahmad Shikh Mousa, Tony Bruns, Cornelius Engelmann, Benjamin Maasoumy, Christian Labenz, Lukas Sturm, Hauke Heinzow, Leon Louis Seifert, Michael Köhler, Max Masthoff, Johnnanes Kluwe, Alexander Zipprich, Christian Jansen, Carsten Meyer, Michael Schultheiss, Michael Praktiknjo. Validation: Markus Kimmann, Nancy Farouk, Michael Praktiknjo. Formal analysis: Markus Kimmann, Nancy Farouk, Michael Praktiknjo. Supervision: Jonel Trebicka, Michael Praktiknjo. Funding acquisition: Michael Praktiknjo. Visualization: Markus Kimmann, Nancy Farouk, Michael Praktiknjo. Project administration: Jonel Trebicka, Michael Praktiknjo. Resources: Jonel Trebicka, Michael Praktiknjo. Writing - original draft: Markus Kimmann, Michael Praktiknjo. Writing - review & editing: Markus Kimmann, Nancy Farouk, Dominik Bettinger, Johannes Chang, Roman Kloeckner, Cristina Ripoll, Felix Piecha, Jassin Rashidi-Alavijeh, Juliana Stadtmann, Ahmad Shikh Mousa, Tony Bruns, Cornelius Engelmann, Benjamin Maasoumy, Christian Labenz, Lukas Sturm, Hauke Heinzow, Leon Louis Seifert, Michael Köhler, Max Masthoff, Johnnanes Kluwe, Alexander Zipprich, Christian Jansen, Carsten Meyer, Michael Schultheiss, Jonel Trebicka, Michael Praktiknjo, German Cirrhosis Study Group: Frank Erhard Uschner, Jörn Arne Meier,

Franziska Weppelmann, Sara Noemi Reinartz Groba, Martin Rössle, Karl Heinz Weiss, Karel Caca, Jens Marquardt, Christian Lange.

Data availability

Requests for data access can be directed to the corresponding author and the German Cirrhosis Study Group and will be granted upon reasonable request.

German Cirrhosis Study Group

Frank Erhard Uschner[1], Jörn Arne Meier[1], Franziska Weppelmann[1], Sara Noemi Reinartz Groba[1], Martin Rössle[2], Marlene Reincke[2], Franziska Schneider[3], Nina Böhling[3], Jakub Grobelski[3], Karl Heinz Weiss[4,5], Karel Caca[6], Jens Marquardt[7], Christian Lange[8]
[1]University Hospital Münster, Medical Clinic B, Münster, Germany
[2]University Hospital Freiburg, Clinic for Internal Medicine II, Freiburg, Germany
[3]University Medical Centre Bonn, Department of Internal Medicine I, Bonn, Germany
[4]Heidelberg University Hospital, Clinic for Gastroenterology, Hepatology, Infectious Diseases, Poisoning, Heidelberg, Germany
[5]Salem Hospital, Internal Medicine, Heidelberg, Germany
[6]RKH Klinikum Ludwigsburg, Internal Medicine, Gastroenterology, Haemato-Oncology, Diabetology and Infectiology, Ludwigsburg, Germany
[7]University Hospital Schleswig-Holstein – Lübeck, Medical Clinic I, Lübeck, Germany
[8]LMU University Hospital of Munich, Department of Internal Medicine II, Munich, Germany

Supplementary data

Supplementary data to this article can be found online at https://doi.org/10.1016/j.jhepr.2026.101795.

References

Author names in bold designate shared co-first authorship

[1] Bloom S, Kemp W, Lubel J. Portal hypertension: pathophysiology, diagnosis and management. Intern Med J 2015;45:16–26.
[2] Iwakiri Y, Trebicka J. Portal hypertension in cirrhosis: pathophysiological mechanisms and therapy. JHEP Rep 2021;3:100316.
[3] Gu W, Hortlik H, Erasmus H-P, et al. Trends and the course of liver cirrhosis and its complications in Germany: nationwide population-based study (2005 to 2018). Lancet Reg Health Eur 2022;12:100240.
[4] Volk ML, Tocco RS, Bazick J, et al. Hospital readmissions among patients with decompensated cirrhosis. Off J Am Coll Gastroenterol ACG 2012;107:247.
[5] Allaire M, Walter A, Sutter O, et al. TIPS for management of portal-hypertension-related complications in patients with cirrhosis. Clin Res Hepatol Gastroenterol 2020;44:249–263.
[6] Larrue H, D'Amico G, Olivas P, et al. TIPS prevents further decompensation and improves survival in patients with cirrhosis and portal hypertension in an individual patient data meta-analysis. J Hepatol 2023;79:692–703.
[7] Trebicka J. Emergency TIPS in a Child-Pugh B patient: when does the window of opportunity open and close? J Hepatol 2017;66:442–450.
[8] Gu W, Zeleke Y, Hortlik H, et al. Use and outcome of TIPS in hospitalized patients in Germany: a Nationwide study (2007-2018). Hepatol Commun 2023;7:e0237.
[9] Ruf AE, Kremers WK, Chavez LL, et al. Addition of serum sodium into the MELD score predicts waiting list mortality better than MELD alone. Liver Transpl 2005;11:336.
[10] Biggins SW, Kim WR, Terrault NA, et al. Evidence-based incorporation of serum sodium concentration into MELD. Gastroenterology 2006;130:1652–1660.
[11] **Nagai S**, **Chau LC**, Schilke RE, et al. Effects of allocating livers for transplantation based on model for end-stage liver disease–sodium scores on patient outcomes. Gastroenterology 2018;155:1451–1462.e3.
[12] Kim WR, Mannalithara A, Heimbach JK, et al. Meld 3.0: the model for end-stage liver disease updated for the modern era. Gastroenterology 2021;161:1887–1895.e4.
[13] Ärzteblatt DÄG Redaktion Deutsches. Richtlinie gem. § 16 Abs. 1 S. 1 Nrn. 2 und 5 TPG für die Wartelistenführung und Organvermittlung zur Lebertransplantation Richtlinie gemäß § 16 Abs. 1 S. 1 Nrn. 2 u. 5 TPG für die Wartelistenführung und Organvermittlung zur Lebertransplantation (RL BÄK Leber). Dtsch Ärztebl 2025.
[14] Rössle M. Liver: early TIPS in patients with cirrhosis and variceal bleeding. Nat Rev Gastroenterol Hepatol 2010;7:536–538.
[15] Franchis R de. Evolving consensus in portal hypertension report of the Baveno IV consensus workshop on methodology of diagnosis and therapy in portal hypertension. J Hepatol 2005;43:167–176.
[16] Franchis R de. Revising consensus in portal hypertension: report of the Baveno V consensus workshop on methodology of diagnosis and therapy in portal hypertension. J Hepatol 2010;53:762–768.
[17] Franchis R de. Expanding consensus in portal hypertension: report of the Baveno VI Consensus Workshop: stratifying risk and individualizing care for portal hypertension. J Hepatol 2015;63:743–752.
[18] Franchis R de, Bosch J, Garcia-Tsao G, et al. Baveno VII – renewing consensus in portal hypertension. J Hepatol 2022;76:959–974.
[19] Gerbes AL, Gülberg V, Sauerbruch T, et al. S3-Leitlinie "Aszites, spontan bakterielle Peritonitis, hepatorenales Syndrom". Z Für Gastroenterol 2011;49:749–779.
[20] Gerbes AL, Labenz J, Appenrodt B, et al. [Updated S2k-Guideline "Complications of liver cirrhosis". German Soc Gastroenterol (DGVS)]. Z Gastroenterol 2019;57:e168.
[21] Kamath PS, Kim WR, Advanced Liver Disease Study Group. The model for end-stage liver disease (MELD). Hepatol Baltim Md 2007;45:797–805.
[22] Bettinger D, Sturm L, Pfaff L, et al. Refining prediction of survival after TIPS with the novel Freiburg index of post-TIPS survival. J Hepatol 2021;74:1362–1372.
[23] Song J, Wang X, Yan Y, et al. MELD 3.0 score for predicting survival in patients with cirrhosis after transjugular intrahepatic portosystemic shunt creation. Dig Dis Sci 2023;68:3185–3192.
[24] Song J, Wang X, Yan Y, et al. MELD 3.0 score for predicting survival in patients with cirrhosis after transjugular intrahepatic portosystemic shunt creation. Dig Dis Sci 2023;68:3185–3192.
[25] Wiering L, Aigner A, Rosmalen M van, et al. Systematic sex-based inequity in the MELD score-based allocation system for liver transplantation in Germany. Transpl. Int 2025;38:13844.
[26] Fozouni L, Wang CW, Lai JC. Sex differences in the association between frailty and sarcopenia in patients with cirrhosis. Clin Transl Gastroenterol 2019;10:e00102.
[27] Gödiker J, Schwind L, Jacob T, et al. Ultrasound-defined sarcopenia independently predicts acute decompensation in advanced chronic liver disease. J Cachexia Sarcopenia Muscle 2024;15:2792–2802.
[28] Delgado MG, Mertineit N, Bosch J, et al. Combination of Model for End-Stage Liver Disease (MELD) and Sarcopenia predicts mortality after transjugular intrahepatic portosystemic shunt (TIPS). Dig Liver Dis Off J Ital Soc Gastroenterol Ital Assoc Study Liver 2024;56:1544–1550.
[29] Schattenberg JM, Chalasani N, Alkhouri N. Artificial intelligence applications in Hepatology. Clin Gastroenterol Hepatol 2023;21:2015–2025.

Keywords: ReMELD-Na; MELD 3.0; transjugular intrahepatic portosystemic shunt; cirrhosis.
Received 9 December 2025; received in revised form 9 February 2026; accepted 17 February 2026; Available online 21 February 2026

Journal of Hepatology, Volume *8*

Supplemental information

Comparative performance of ReMELD-Na, MELD 3.0 and established scores after TIPS for refractory ascites: A multicenter study

Markus Kimmann, Nancy Farouk, Dominik Bettinger, Johannes Chang, Roman Kloeckner, Cristina Ripoll, Felix Piecha, Jassin Rashidi-Alavijeh, Juliana Stadtmann, Ahmad Shikh Mousa, Tony Bruns, Cornelius Engelmann, Benjamin Maasoumy, Christian Labenz, Lukas Sturm, Hauke Heinzow, Leon Louis Seifert, Michael Köhler, Max Masthoff, Johannes Kluwe, Alexander Zipprich, Christian Jansen, Carsten Meyer, Michael Schultheiss, Jonel Trebicka, Michael Praktiknjo, and German Cirrhosis Study Group

Comparative performance of ReMELD-Na, MELD 3.0 and established scores after TIPS for refractory ascites: A multicenter study

Markus Kimmann, Nancy Farouk, Dominik Bettinger, Johannes Chang, Roman Kloeckner, Cristina Ripoll, Felix Piecha, Jassin Rashidi-Alavijeh, Juliana Stadtmann, Ahmad Shikh Mousa, Tony Bruns, Cornelius Engelmann, Benjamin Maasoumy, Christian Labenz, Lukas Sturm, Hauke Heinzow, Leon Louis Seifert, Michael Köhler, Max Masthoff, Johannes Kluwe, Alexander Zipprich, Christian Jansen, Carsten Meyer, Michael Schultheiss, Jonel Trebicka, Michael Praktiknjo, German Cirrhosis Study Group

Table of contents

Supplementary text 1……………………………………………………………………2

Supplementary text 2……………………………………………………………………3

Supplementary figures………………………………………………………………...…..4

Supplementary tables…………………………………………………………………….27

Supplementary references…………………………………………………………......36

Supplementary text 1

Calculation of the scores:

MELD = (0.957*ln(creatinine) + 0.378*ln(bilirubin) + 1.120*ln(INR) + 0.643)*10 (dialysis treatment twice a week sets a creatinine value of 4 mg/dl, lower capping for every value of 1, upper capping of 4 mg/dl for creatinine).

MELD-Na = MELD + 1,32*(137-sodium) – (0.033*MELD*(137-sodium) (lower and upper capping of 125-137 mmol/L for sodium).

MELD 3.0 = 1.33 (if female) + 4.56*ln(bilirubin) + 0.82*(137-Na) – 0.24*(137 Na)*ln(bilirubin) + 9.09*ln(INR) + 11.14*ln(creatinine) + 1.85*(3.5 – albumin) – 1.83*(3.5 – albumin)*ln(creatinine) + 6 (lower and upper capping of 125-137 mmol/L for sodium and 1.5-3.5 g/dl for albumin, upper capping of 3 mg/dl for creatinine).

FIPS = 1.43*log10(bilirubin) – 1.71*1/creatinine + 0.02*age – 0.02*albumin + 0.81

ReMELD-Na = 7,85 + 9,03*ln(creatinine) + 2,97*ln(bilirubin) + 9,52*ln(INR) + 0,392*(138,6-sodium) – 0,351*(138,6-sodium)*ln(creatinine) (lower and upper capping of 0.7-0.25 mg/dl for creatinine, 0.3-27 mg/dl for bilirubin, 1.0-2.6 for INR and 120-138.6 mmol/L for sodium).

Supplementary text 2

Description of the spline analyses using R:

The spline analyses were purely descriptive visualizations and were not embedded in a logistic or Cox regression model. While the study outcomes (90-day and one-year combined event (Death/LTx)-free survival) are intrinsically time-to-event outcomes, the spline figures did not use any form of survival modelling. For plotting purposes, the outcome was used only as a binary grouping variable (event vs. event-free), but no logistic regression was fitted. Instead, for each pairwise score comparison, we visualized the relationship between the x-axis score (ReMELD-Na or MELD 3.0) and the comparator score using separate smooth curves for event and non-event patients. These curves were generated in ggplot2 (R) using:

geom_smooth(method = "lm", formula = y ~ ns(x, df = 3)),

which fits a simple linear model with a natural cubic spline transformation of the x-variable. No risk modelling was applied in these plots.

All spline curves used natural cubic splines (ns() function, splines package in R) with 3 degrees of freedom. For natural cubic splines, the number of interior knots is always equal to df − 1; therefore, df = 3 corresponds to two interior knots, with the remaining flexibility defined by the natural boundary constraints. This specification was applied identically across all comparisons, both for ReMELD-Na and for MELD 3.0, and separate spline fits were generated for the event and non-event groups. The knot locations were not set manually. When using ns() with df = 3, R automatically places the two interior knots at the empirical 33rd and 67th percentiles of the respective x-axis variable (ReMELD-Na or MELD 3.0) within each outcome subgroup, and the boundary knots at the minimum and maximum values. This is the default behaviour of ns() and was used throughout the analysis.

Supplementary figures

1

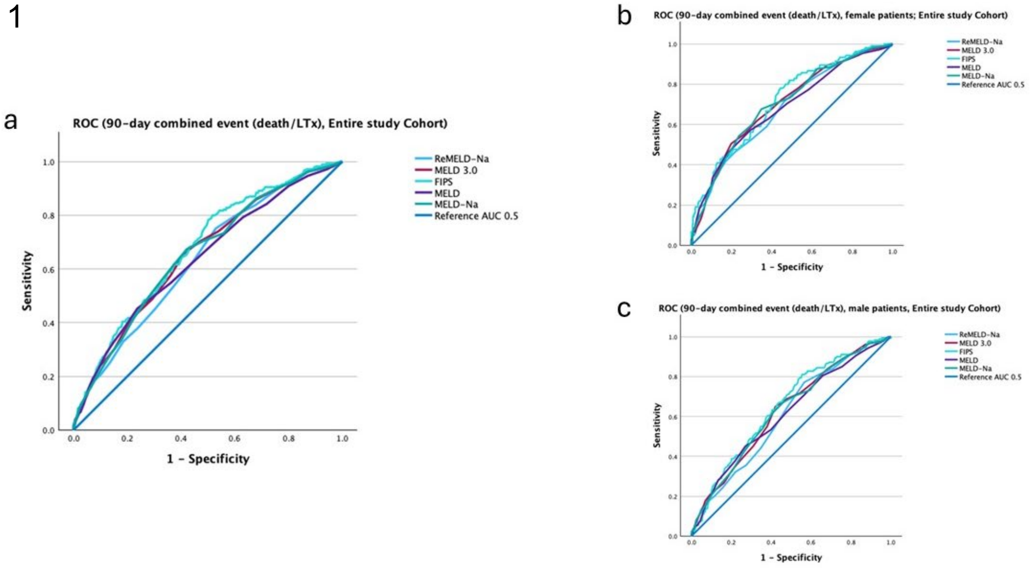

Fig. S1: Results of the Receiver operating characteristic (ROC) analysis in the entire study cohort with ROC curves displayed for ReMELD-Na, MELD 3.0, FIPS, MELD and MELD-Na for the entire cohort (a) as well as the female (b) and male subcohorts (c) separately. Outcome: 90-day combined event (Death/LTx).

2

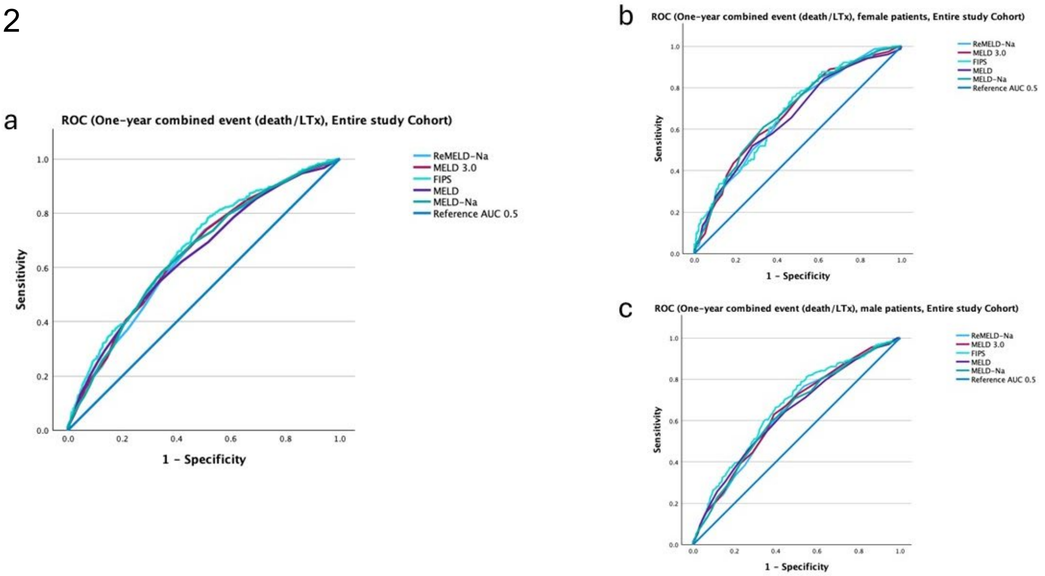

Fig. S2: Results of the Receiver operating characteristic (ROC) analysis in the entire study cohort with ROC curves displayed for ReMELD-Na, MELD 3.0, FIPS, MELD and MELD-Na for the entire cohort (a) as well as the female (b) and male subcohorts (c) separately. Outcome: One-year combined event (Death/LTx).

Supplementary Figure 3

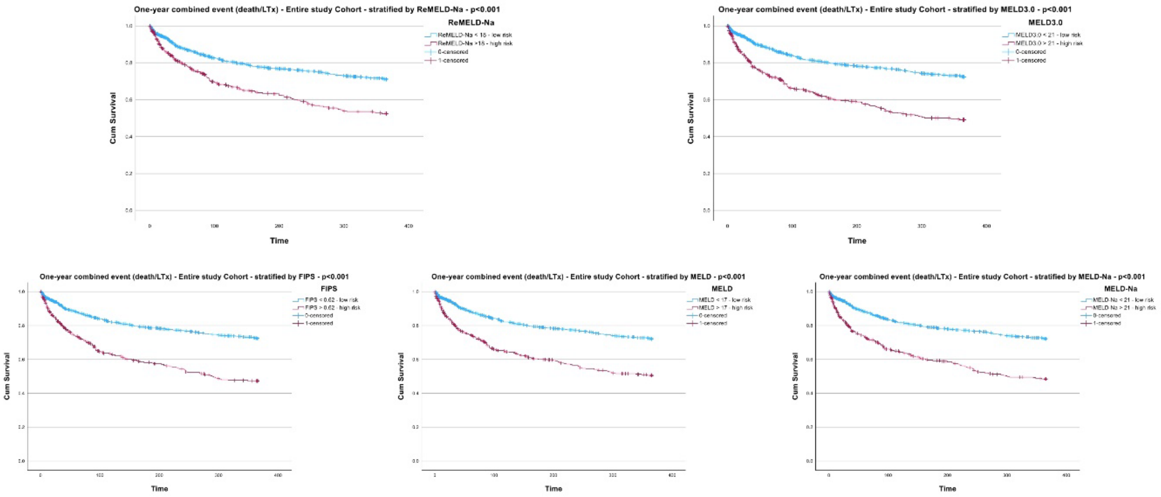

Fig. S3: Kaplan-Meier curves showing one-year combined event (Death/LTx)-free survival for high- vs. low-risk groups, stratified by the 75th percentile of each scoring system. Cutoff values used to define high-risk groups were: ReMELD-Na = 18, MELD 3.0 = 21, FIPS = 0.62, MELD = 17, and MELD-Na = 21. Level of significance for each Kaplan-Meier curve: $p < 0.001$ (log-rank test).

4a

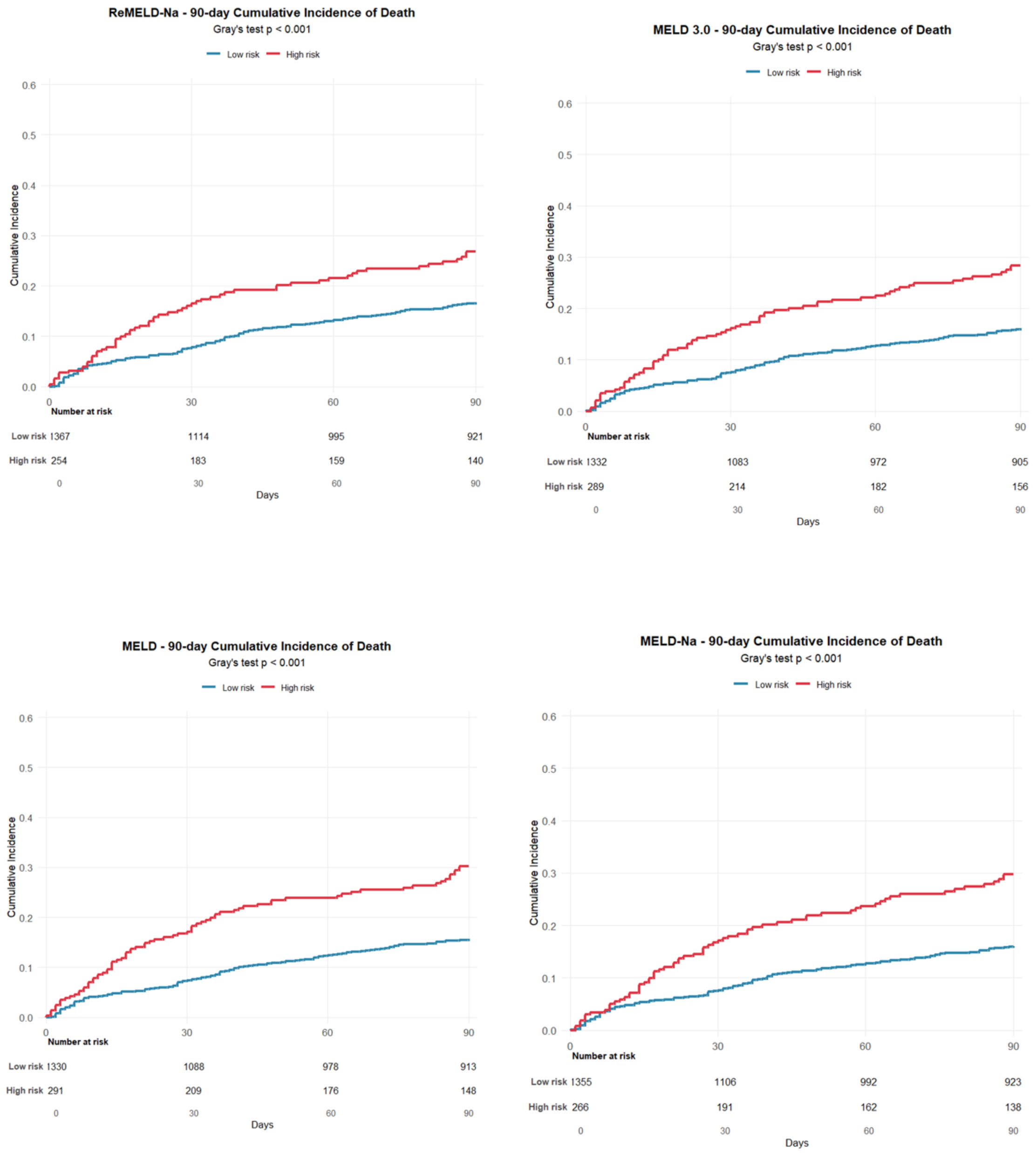
ReMELD-Na - 90-day Cumulative Incidence of Death
Gray's test p < 0.001
Low risk
High risk
Cumulative Incidence
0.0
0.1
0.2
0.3
0.4
0.5
0.6
0
30
60
90
Number at risk
Low risk 1367 1114 995 921
High risk 254 183 159 140
Days
MELD 3.0 - 90-day Cumulative Incidence of Death
Gray's test p < 0.001
Low risk
High risk
Cumulative Incidence
Number at risk
Low risk 1332 1083 972 905
High risk 289 214 182 156
Days
MELD - 90-day Cumulative Incidence of Death
Gray's test p < 0.001
Low risk
High risk
Cumulative Incidence
Number at risk
Low risk 1330 1088 978 913
High risk 291 209 176 148
Days
MELD-Na - 90-day Cumulative Incidence of Death
Gray's test p < 0.001
Low risk
High risk
Cumulative Incidence
Number at risk
Low risk 1355 1106 992 923
High risk 266 191 162 138
Days

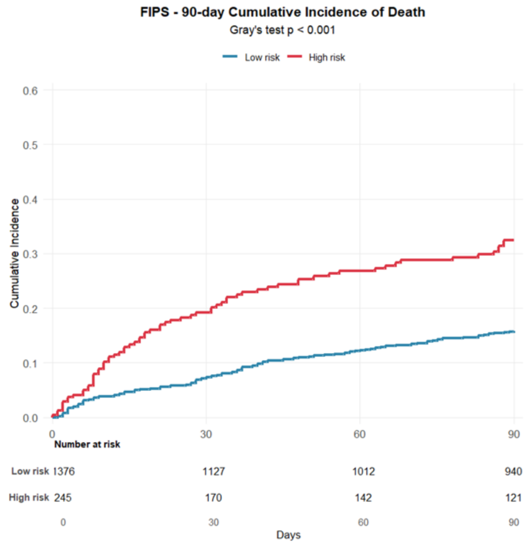

Fig. S4a: Fine-Gray proportional hazard analysis showing 90-day and one-year cumulative incidence of death under the competing risk of LTx for high- vs. low-risk groups, stratified by the 85th (90-day) percentile of each scoring system. Cutoff values used to define high-risk groups were: ReMELD-Na = 20, MELD 3.0 = 23, FIPS = 0.92, MELD = 19, and MELD-Na = 23. Level of significance for each analysis: $p < 0.001$ (Fine-Gray proportional hazard analysis).

4b

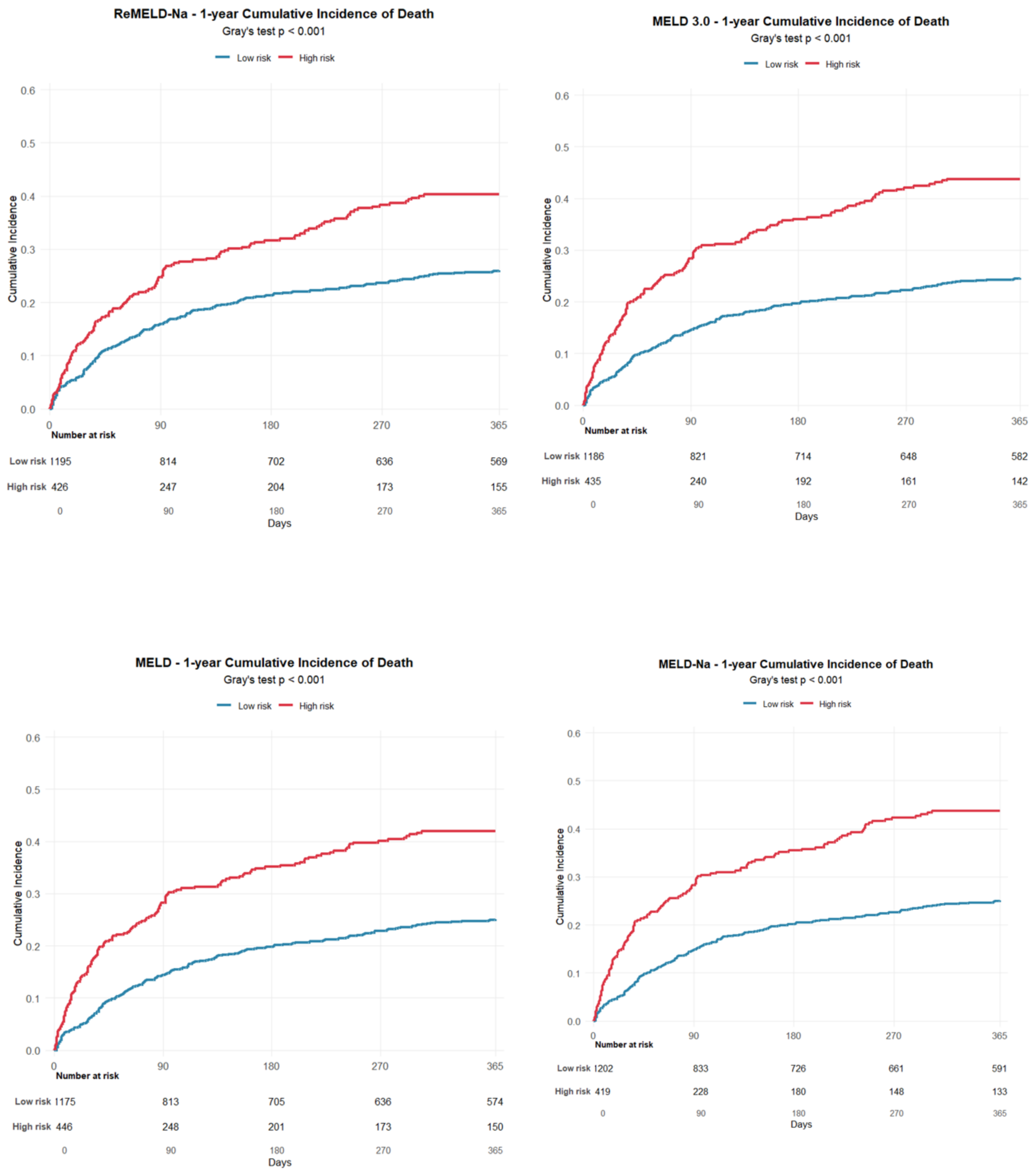
ReMELD-Na - 1-year Cumulative Incidence of Death
Gray's test p < 0.001
Low risk
High risk
Cumulative Incidence
Number at risk
Low risk 1195 814 702 636 569
High risk 426 247 204 173 155
Days
MELD 3.0 - 1-year Cumulative Incidence of Death
Gray's test p < 0.001
Low risk
High risk
Cumulative Incidence
Number at risk
Low risk 1186 821 714 648 582
High risk 435 240 192 161 142
Days
MELD - 1-year Cumulative Incidence of Death
Gray's test p < 0.001
Low risk
High risk
Cumulative Incidence
Number at risk
Low risk 1175 813 705 636 574
High risk 446 248 201 173 150
Days
MELD-Na - 1-year Cumulative Incidence of Death
Gray's test p < 0.001
Low risk
High risk
Cumulative Incidence
Number at risk
Low risk 1202 833 726 661 591
High risk 419 228 180 148 133
Days

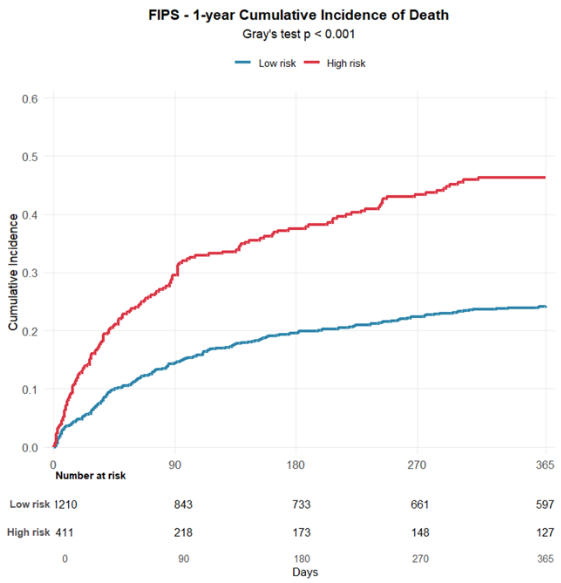

Fig. S4b: Fine-Gray proportional hazard analysis showing 90-day and one-year cumulative incidence of death under the competing risk of LTx for high- vs. low-risk groups, stratified by the 75th (one-year) percentile of each scoring system. Cutoff values used to define high-risk groups were: ReMELD-Na = 18, MELD 3.0 = 21, FIPS = 0.62, MELD = 17, and MELD-Na = 21. Level of significance for each analysis: $p < 0.001$ (Fine-Gray proportional hazard analysis).

Fig. S5a

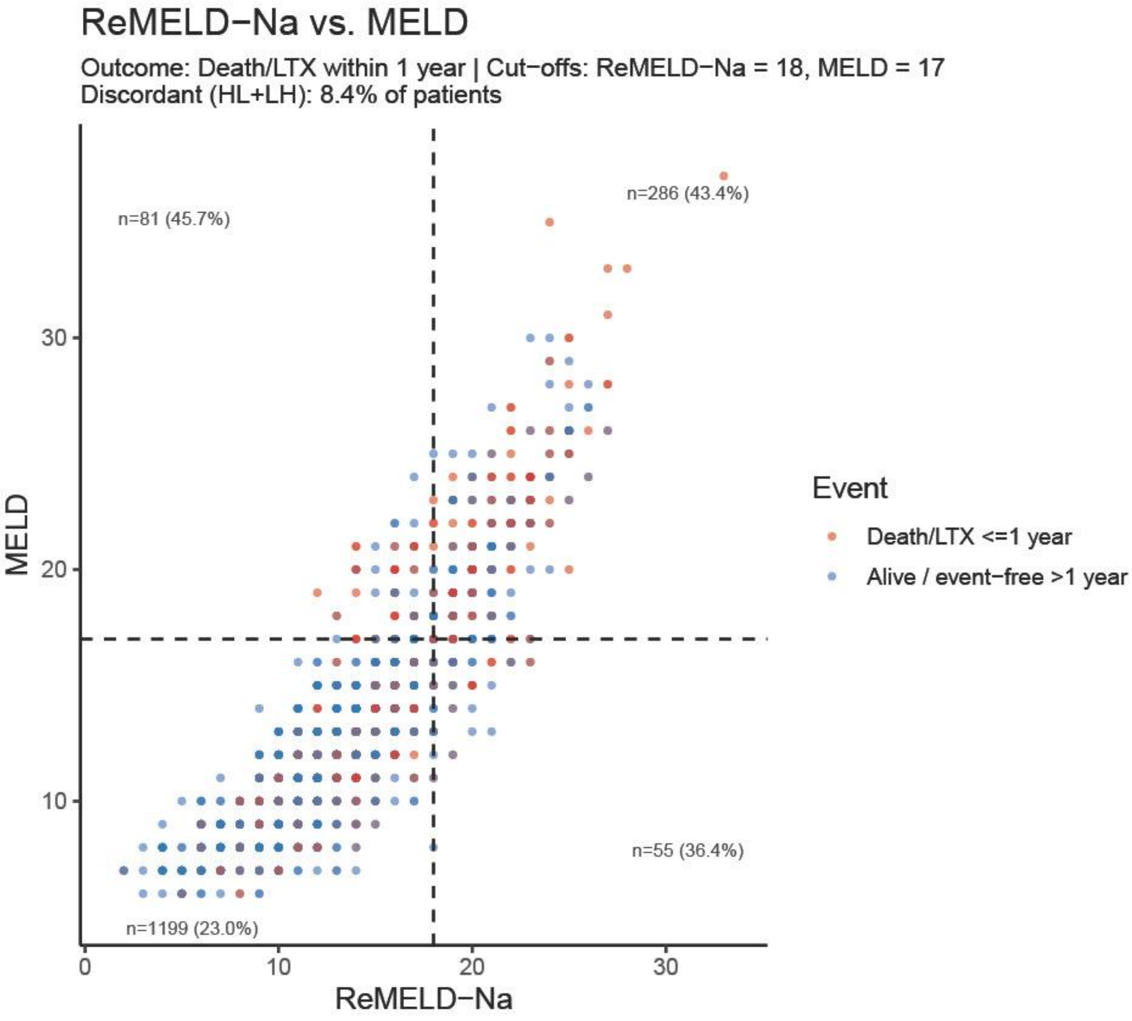
ReMELD−Na vs. MELD
Outcome: Death/LTX within 1 year | Cut−offs: ReMELD−Na = 18, MELD = 17
Discordant (HL+LH): 8.4% of patients
n=81 (45.7%)
n=286 (43.4%)
n=55 (36.4%)
n=1199 (23.0%)
MELD
ReMELD−Na
Event
Death/LTX <=1 year
Alive / event−free >1 year

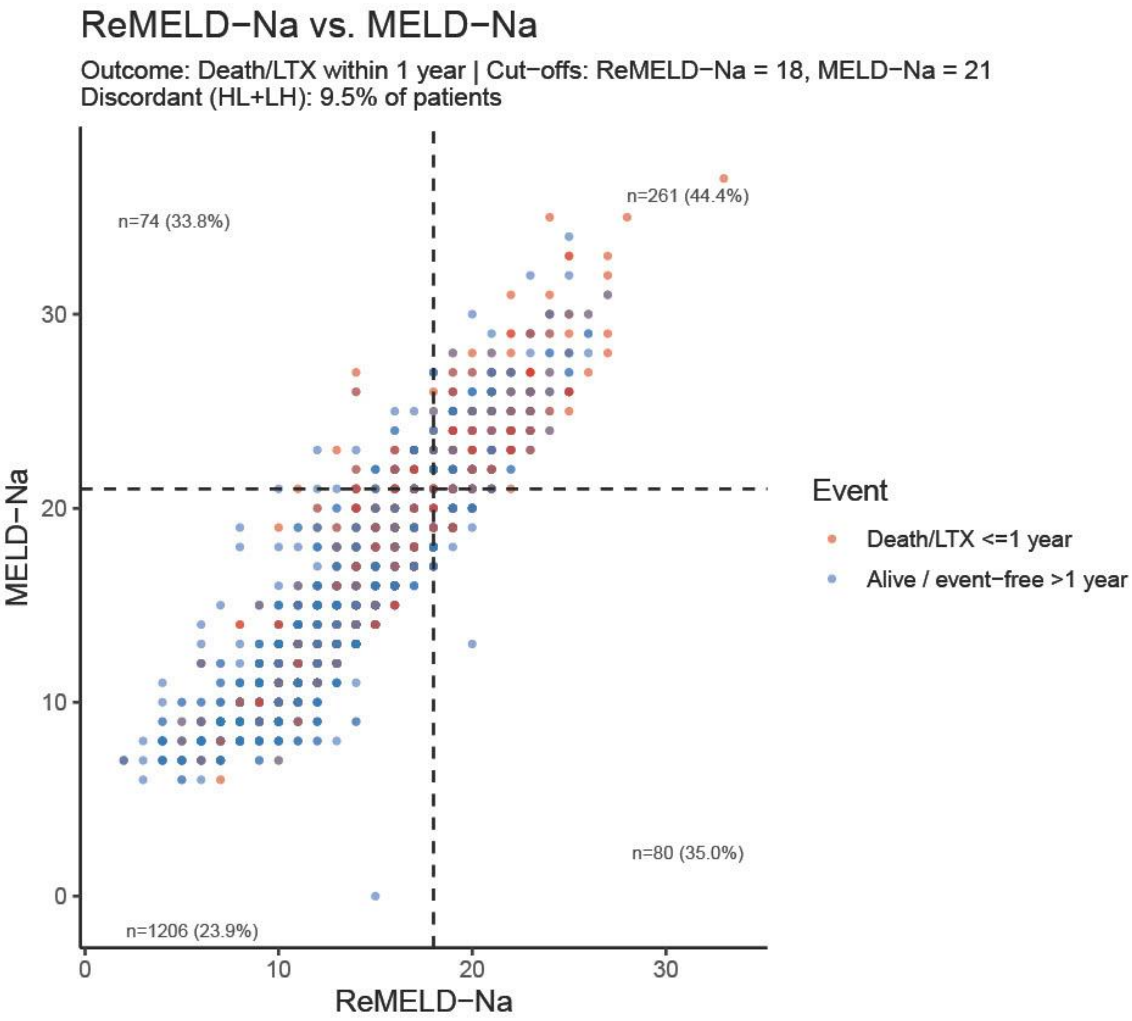
ReMELD−Na vs. MELD−Na
Outcome: Death/LTX within 1 year | Cut−offs: ReMELD−Na = 18, MELD−Na = 21
Discordant (HL+LH): 9.5% of patients
n=74 (33.8%)
n=261 (44.4%)
n=80 (35.0%)
n=1206 (23.9%)
MELD−Na
ReMELD−Na
0
10
20
30
Event
Death/LTX <=1 year
Alive / event−free >1 year

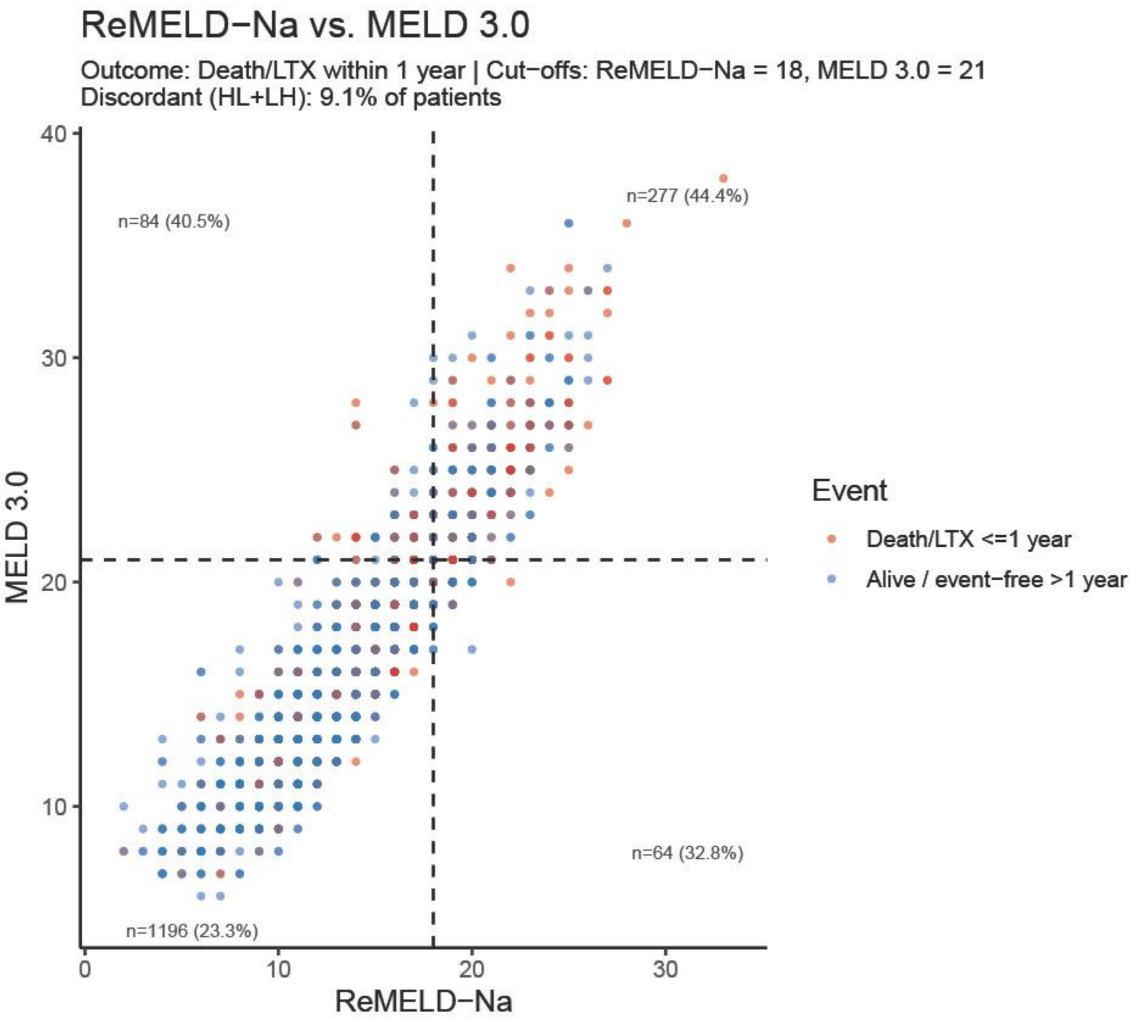

ReMELD−Na vs. MELD 3.0
Outcome: Death/LTX within 1 year | Cut−offs: ReMELD−Na = 18, MELD 3.0 = 21
Discordant (HL+LH): 9.1% of patients
n=84 (40.5%)
n=277 (44.4%)
n=64 (32.8%)
n=1196 (23.3%)
MELD 3.0
ReMELD−Na
Event
Death/LTX <=1 year
Alive / event−free >1 year

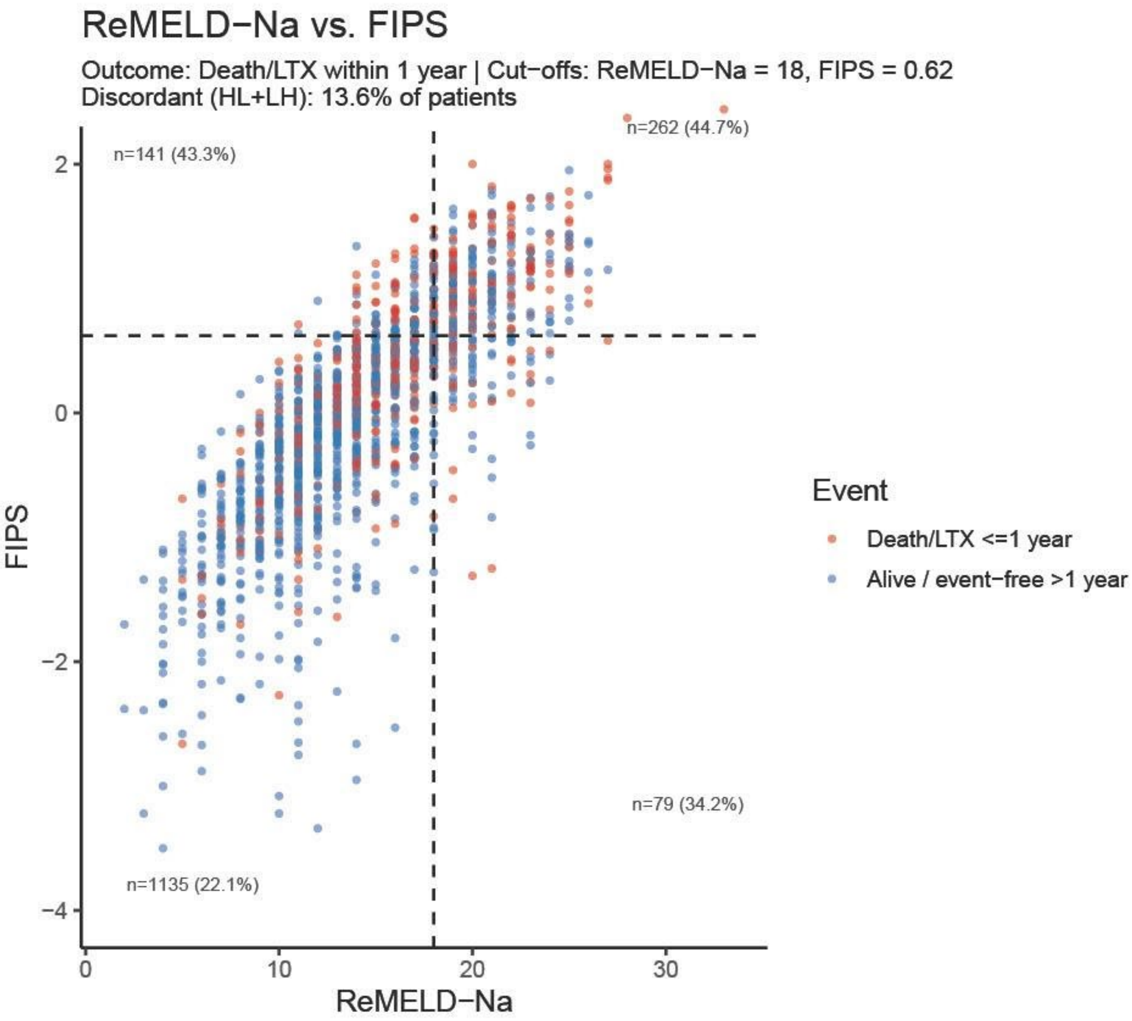
ReMELD−Na vs. FIPS
Outcome: Death/LTX within 1 year | Cut−offs: ReMELD−Na = 18, FIPS = 0.62
Discordant (HL+LH): 13.6% of patients
n=141 (43.3%)
n=262 (44.7%)
n=79 (34.2%)
n=1135 (22.1%)
FIPS
ReMELD−Na
Event
Death/LTX <=1 year
Alive / event−free >1 year

Fig. S5b

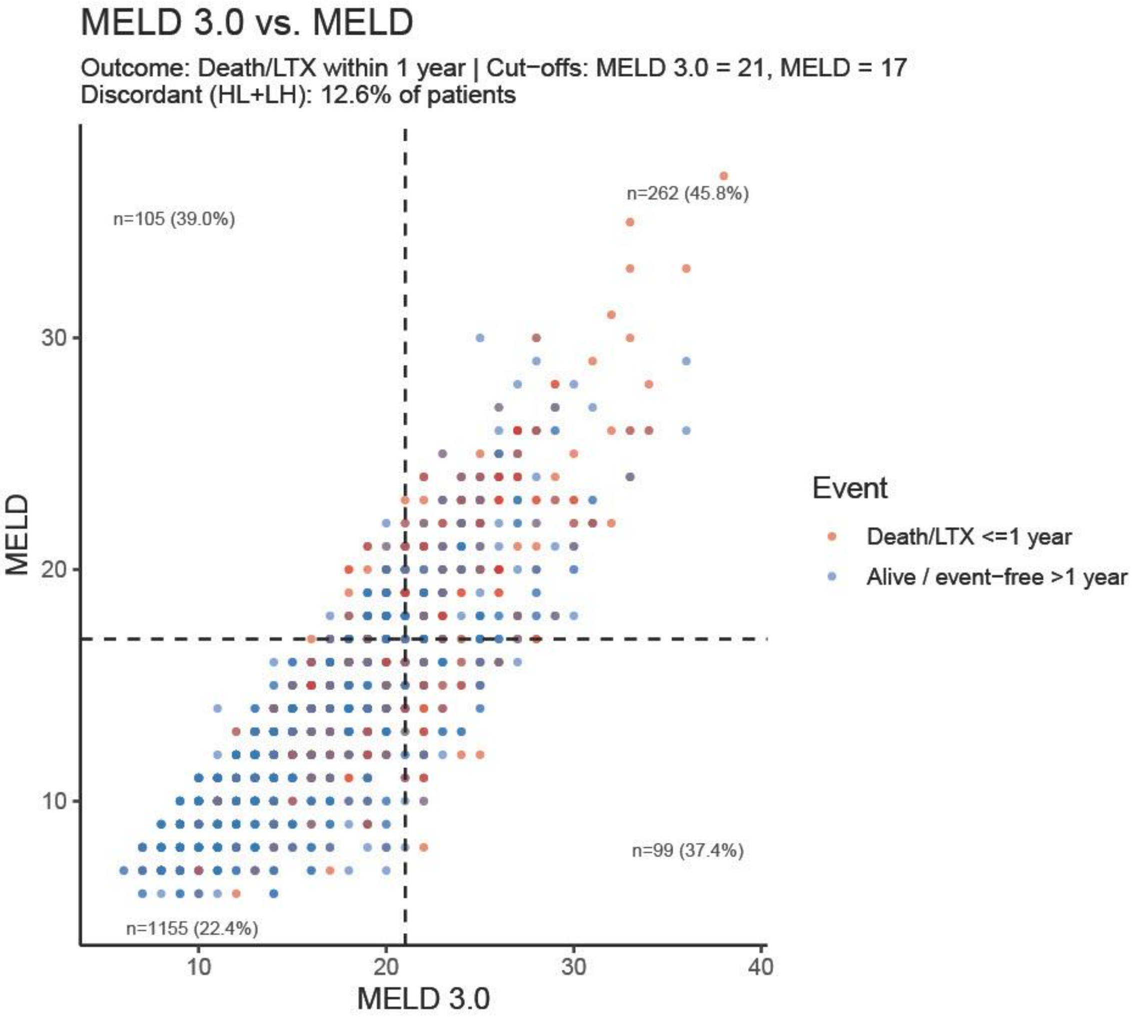

MELD 3.0 vs. MELD
Outcome: Death/LTX within 1 year | Cut−offs: MELD 3.0 = 21, MELD = 17
Discordant (HL+LH): 12.6% of patients
n=105 (39.0%)
n=262 (45.8%)
n=99 (37.4%)
n=1155 (22.4%)
MELD
30
20
10
MELD 3.0
10
20
30
40
Event
Death/LTX <=1 year
Alive / event−free >1 year

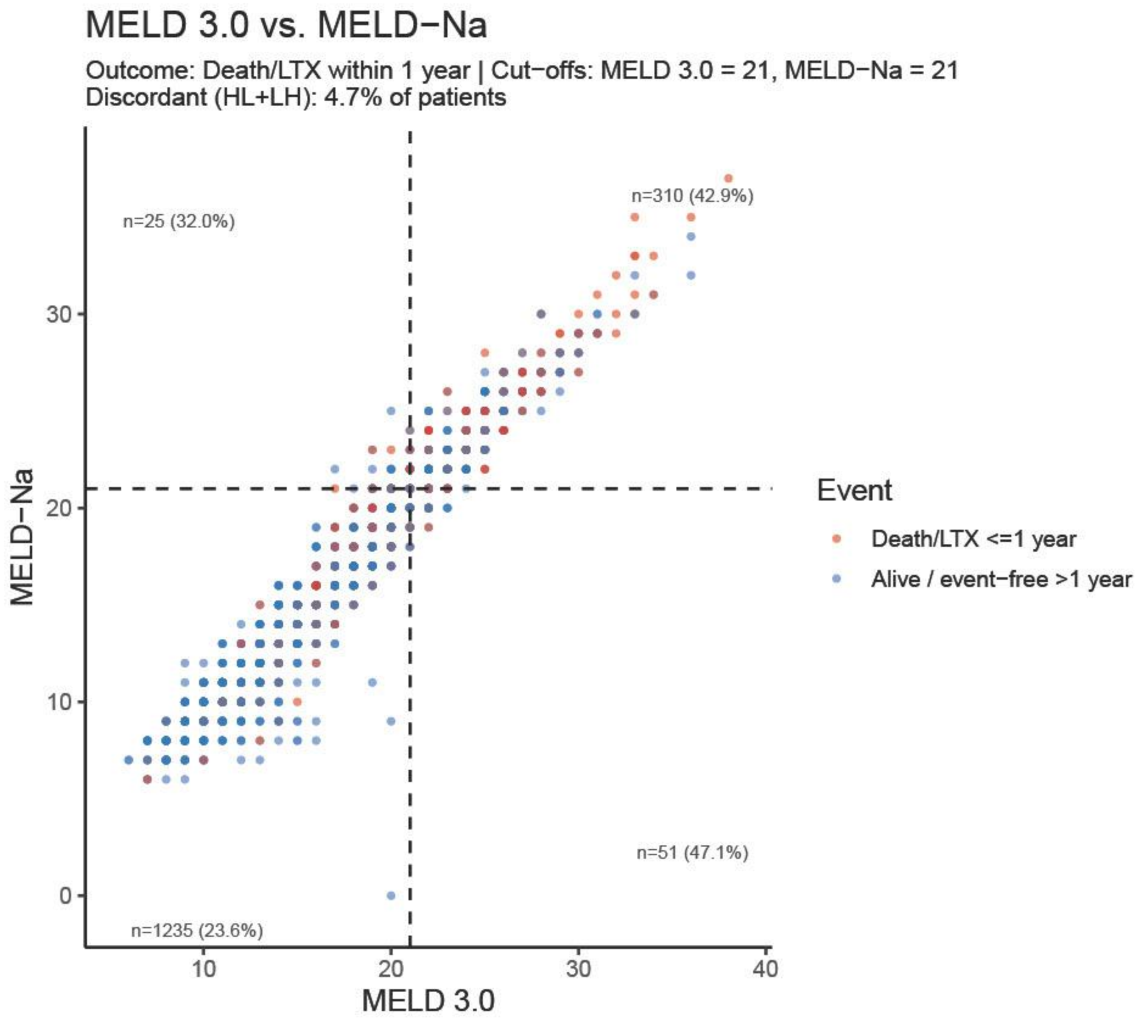
MELD 3.0 vs. MELD−Na
Outcome: Death/LTX within 1 year | Cut−offs: MELD 3.0 = 21, MELD−Na = 21
Discordant (HL+LH): 4.7% of patients
n=25 (32.0%)
n=310 (42.9%)
n=51 (47.1%)
n=1235 (23.6%)
MELD−Na
MELD 3.0
0
10
20
30
40
Event
Death/LTX <=1 year
Alive / event−free >1 year

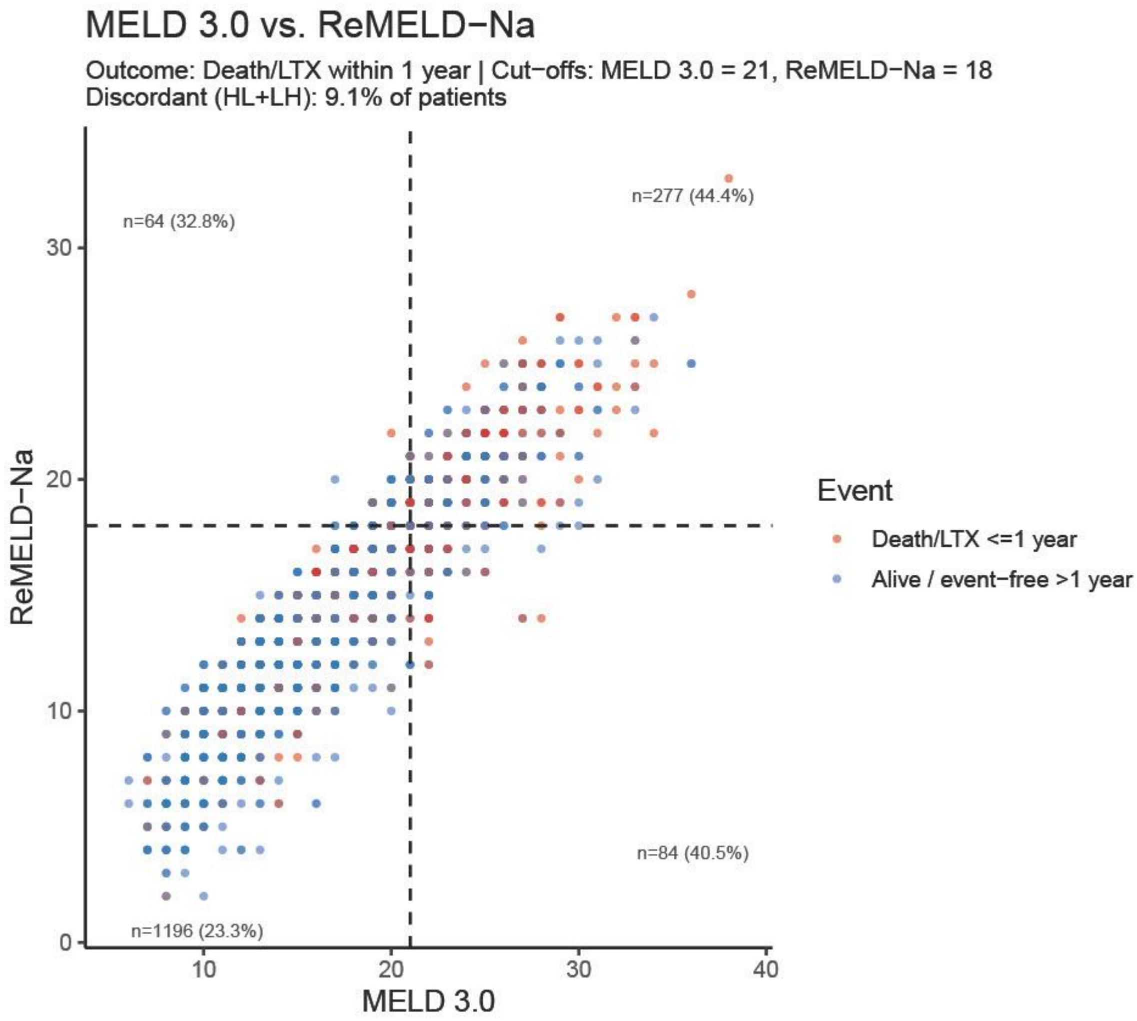
MELD 3.0 vs. ReMELD−Na
Outcome: Death/LTX within 1 year | Cut−offs: MELD 3.0 = 21, ReMELD−Na = 18
Discordant (HL+LH): 9.1% of patients
n=64 (32.8%)
n=277 (44.4%)
n=84 (40.5%)
n=1196 (23.3%)
ReMELD−Na
MELD 3.0
Event
Death/LTX <=1 year
Alive / event−free >1 year

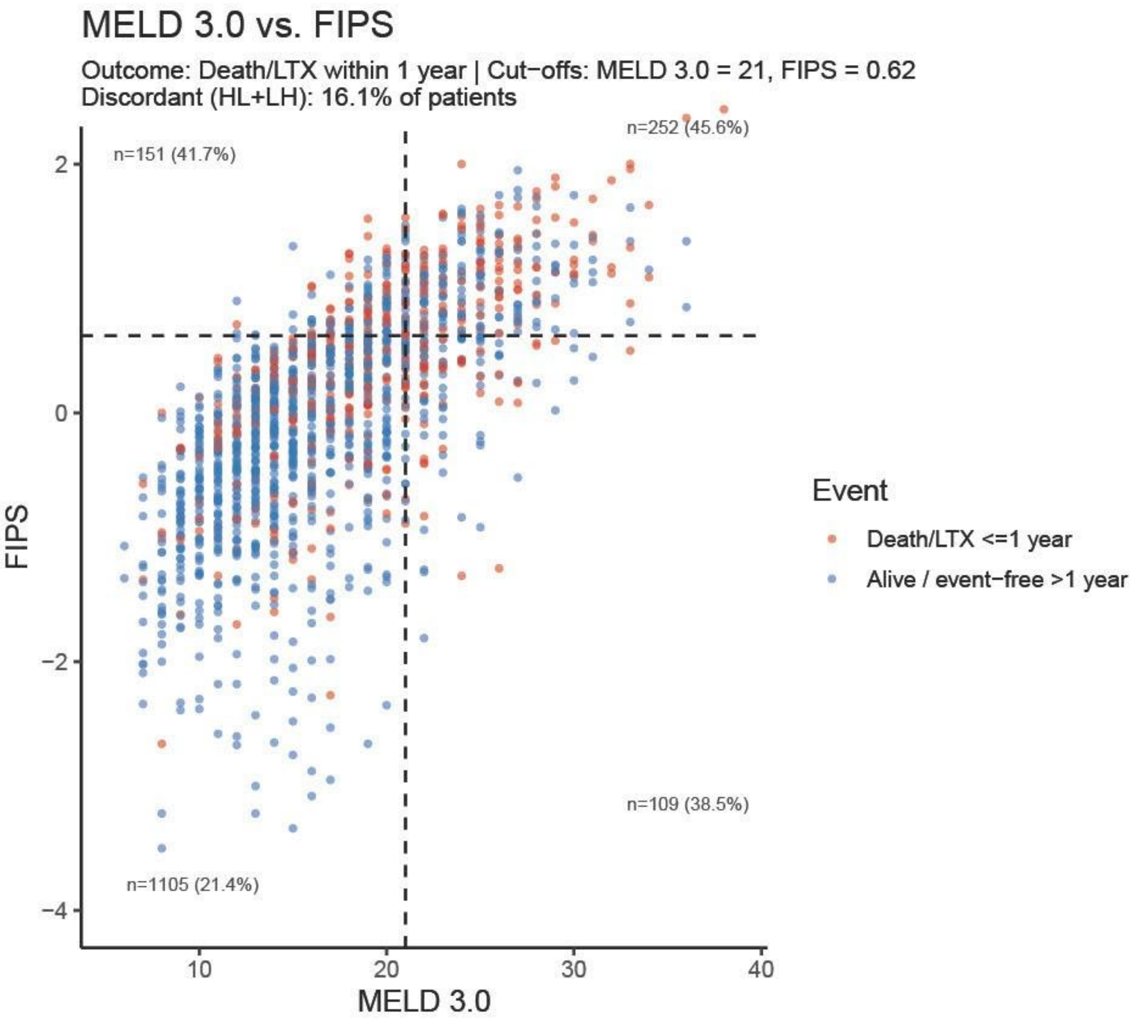

Fig. S5: Scatter plots of patients who reached the combined endpoint (Death/LTx) before censoring (orange) and those who did not (blue) within one year. **a)** Cube positions reflect ReMELD-Na (x-axis) and MELD, MELD-Na, MELD 3.0, or FIPS (y-axis). **b)** Cube positions reflect MELD 3.0 (x-axis) and MELD, MELD-Na, ReMELD-Na, or FIPS (y-axis). Based on high- vs. low-risk thresholds (ReMELD-Na = 18, MELD 3.0 = 21, FIPS = 0.62, MELD = 17, MELD-Na = 21), a 2×2 grid was defined: lower left = low/low-risk, lower right = high/low-risk, upper left = low/high-risk, upper right = high/high-risk. Lower right + upper left show discordant patients. Each quadrant shows n (patients) and % (events).

Fig. S6a

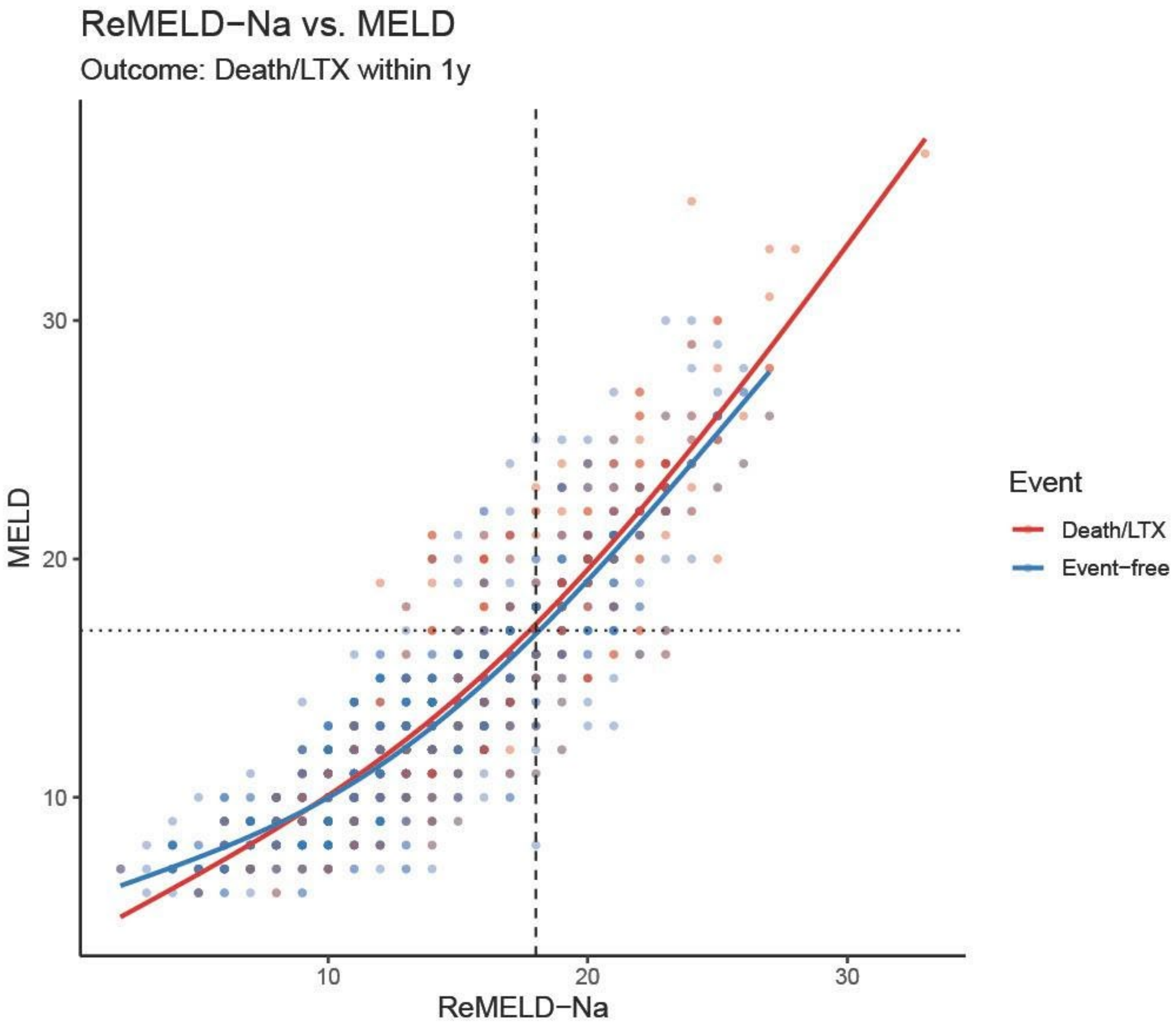

ReMELD−Na vs. MELD
Outcome: Death/LTX within 1y
MELD
30
20
10
ReMELD−Na
10
20
30
Event
Death/LTX
Event−free

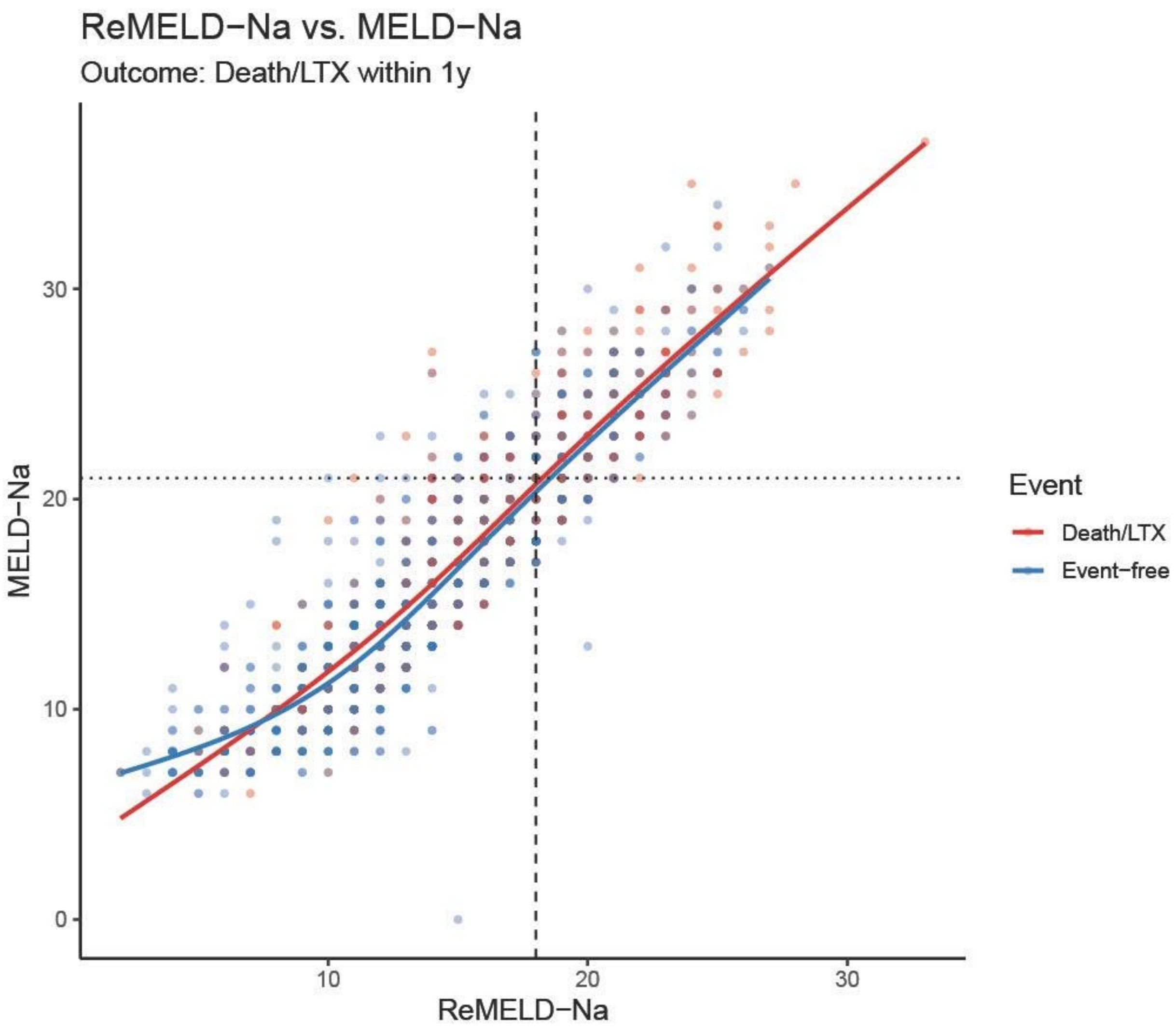
ReMELD−Na vs. MELD−Na
Outcome: Death/LTX within 1y
MELD−Na
ReMELD−Na
0
10
20
30
10
20
30
Event
Death/LTX
Event−free

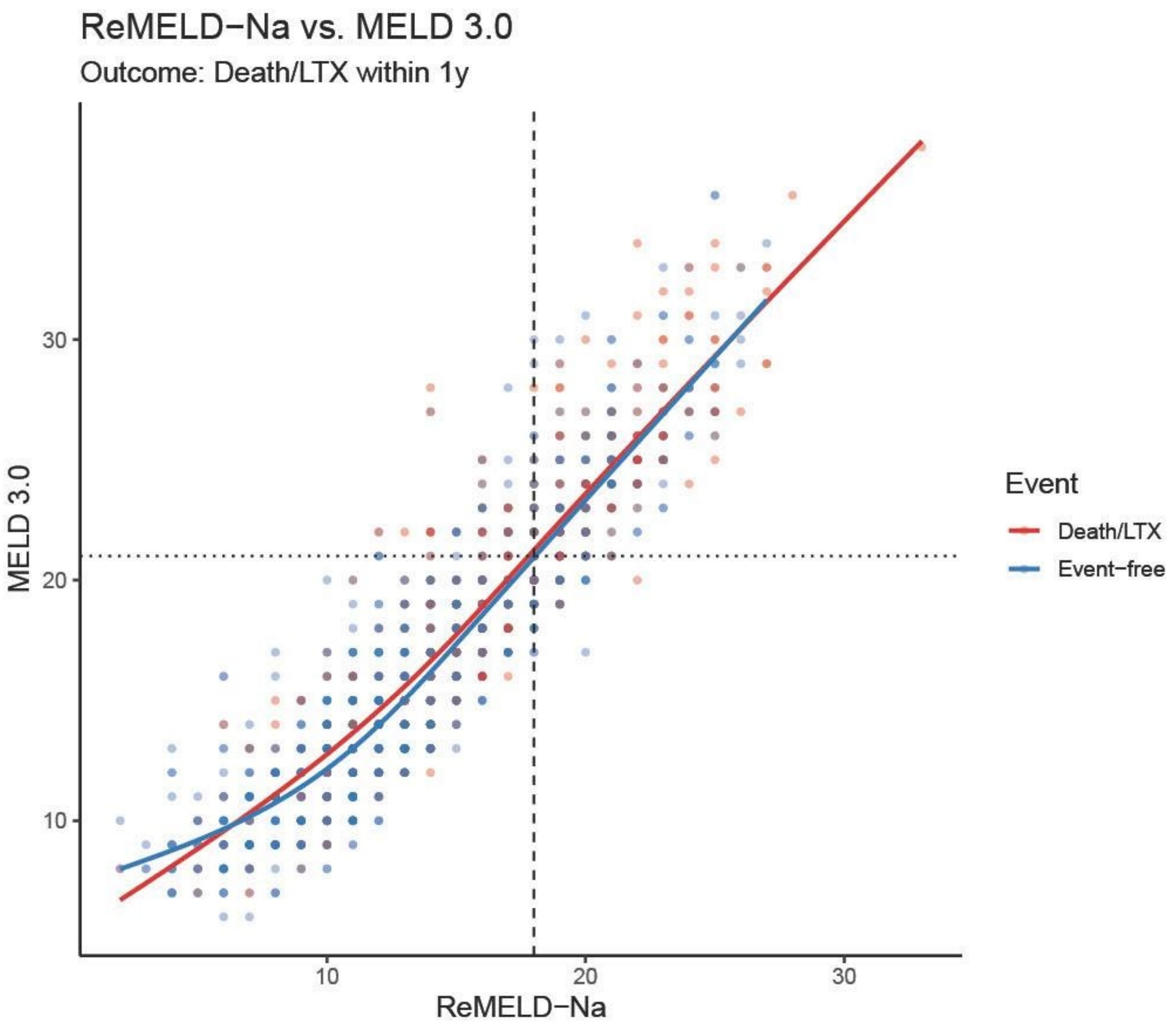
ReMELD−Na vs. MELD 3.0
Outcome: Death/LTX within 1y
MELD 3.0
30
20
10
10
20
30
ReMELD−Na
Event
Death/LTX
Event−free

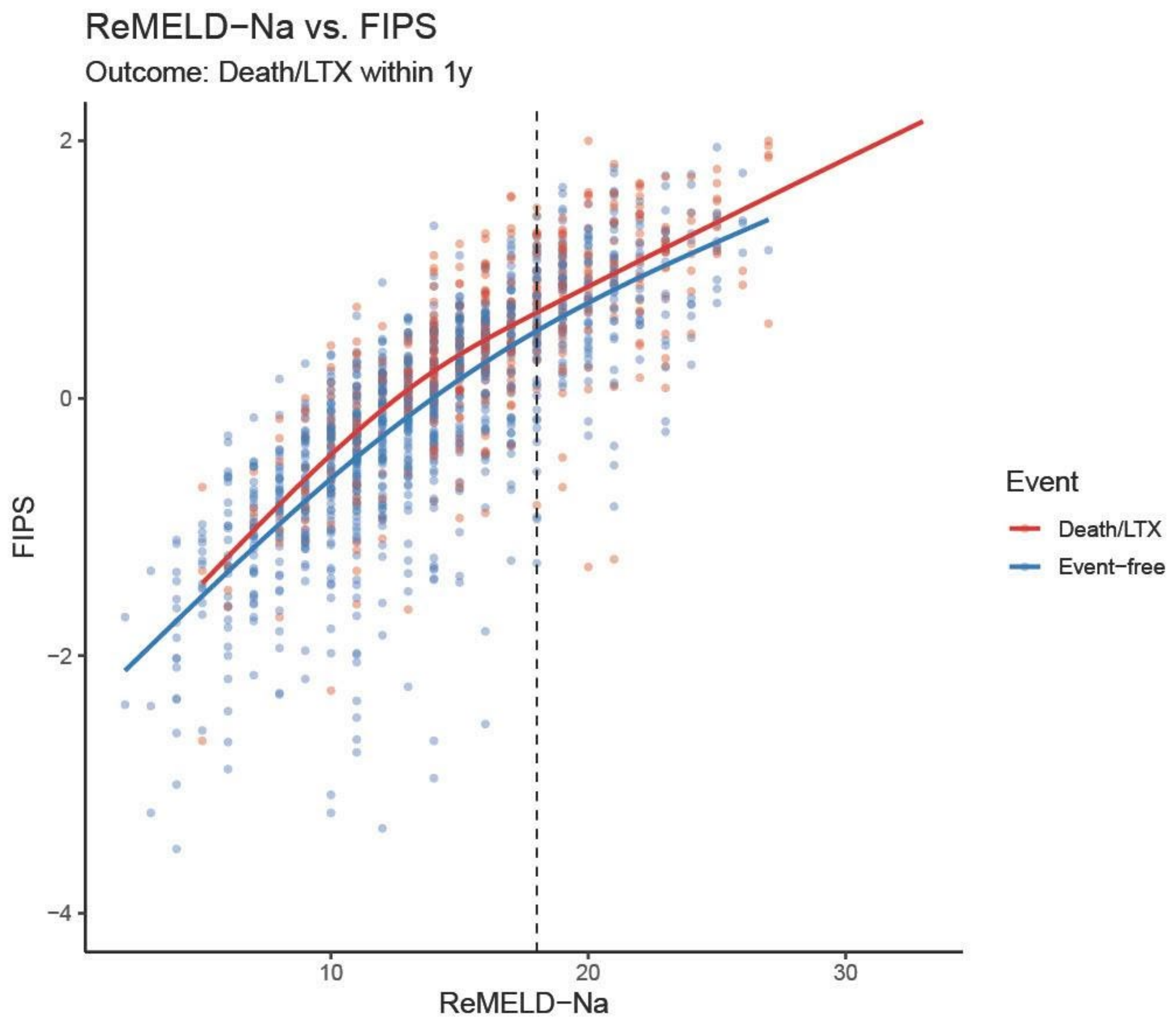
ReMELD−Na vs. FIPS
Outcome: Death/LTX within 1y
2
0
−2
−4
FIPS
10
20
30
ReMELD−Na
Event
Death/LTX
Event−free

Fig. S6b

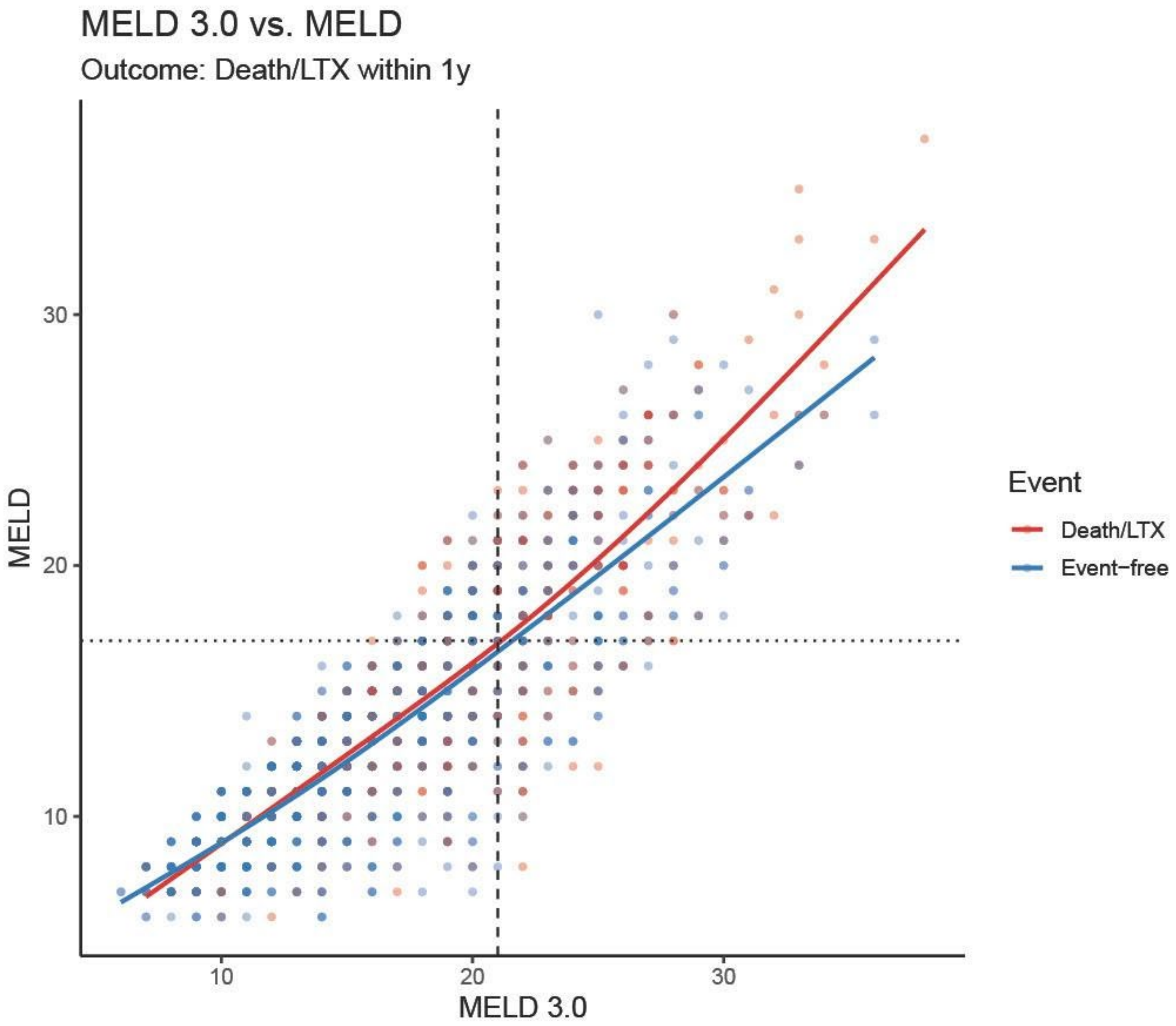
MELD 3.0 vs. MELD
Outcome: Death/LTX within 1y
MELD
30
20
10
10
20
30
MELD 3.0
Event
Death/LTX
Event−free

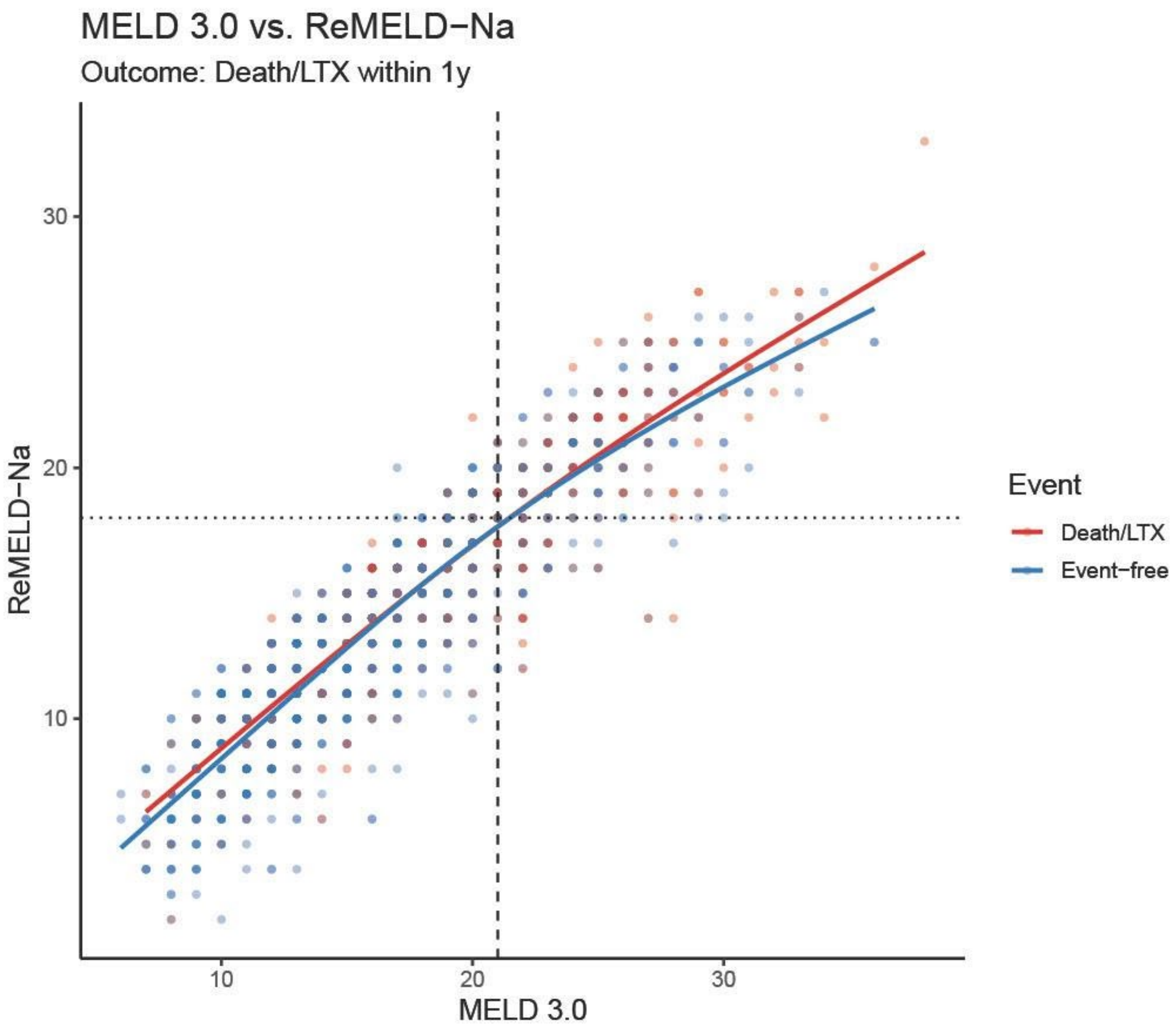
MELD 3.0 vs. ReMELD−Na
Outcome: Death/LTX within 1y
ReMELD−Na
MELD 3.0
10
20
30
10
20
30
Event
Death/LTX
Event−free

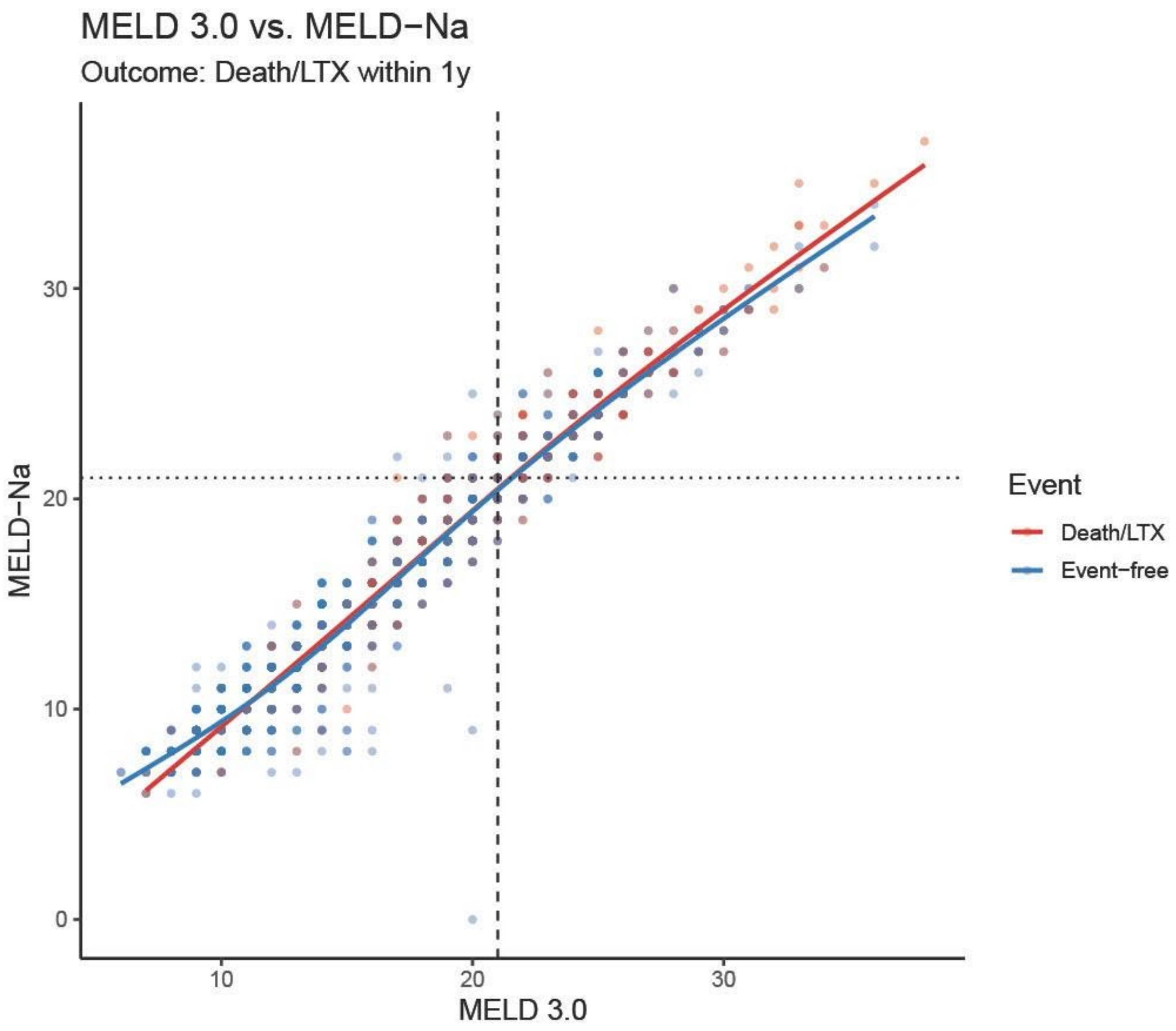
MELD 3.0 vs. MELD−Na
Outcome: Death/LTX within 1y
MELD−Na
MELD 3.0
0
10
20
30
10
20
30
Event
Death/LTX
Event−free

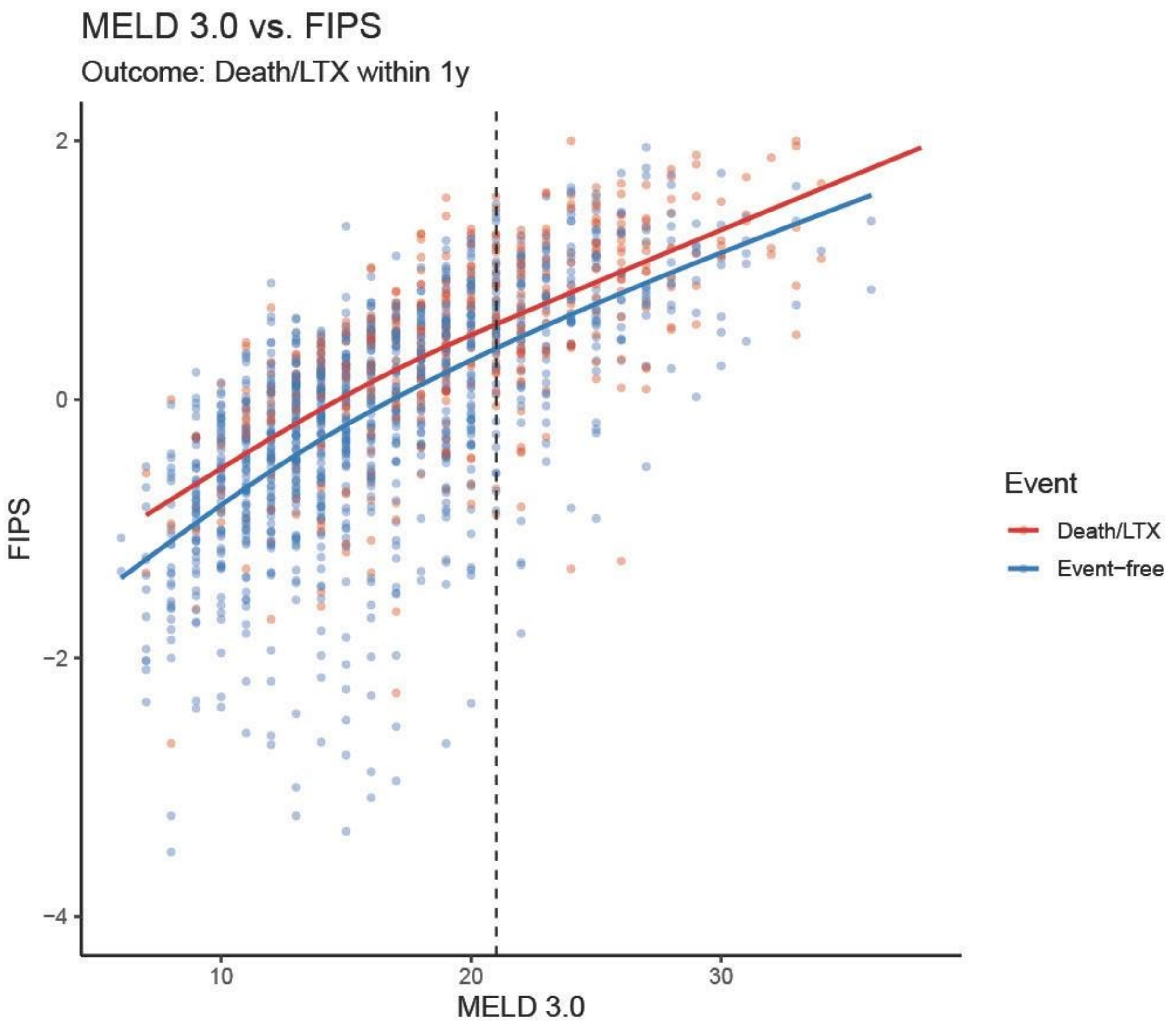

Fig. S6: Descriptive score-vs-score spline smooth analyses of patients who reached the combined endpoint (death/LTx) before censoring or loss-to follow-up (orange) and those who did not (blue) within one year, depicting the association between prognostic scores and events. **a)** Cube positions reflect ReMELD-Na (x-axis) and MELD, MELD-Na, MELD 3.0, or FIPS (y-axis). **b)** Cube positions reflect MELD 3.0 (x-axis) and MELD, MELD-Na, ReMELD-Na, or FIPS (y-axis).

Supplementary tables

1a)						
**Entire Cohort**	**Female (n = 579)**	**Male (n = 1042)**	**Difference (ROC-AUC female-male)**	**Female (n = 579)**	**Male (n = 1042)**	**Difference (ROC-AUC female-male)**
**(90-day combined event (Death/LTx))**	**No alcohol-related cirrhosis**	**No alcohol-related cirrhosis**		**Alcohol-related cirrhosis**	**Alcohol-related cirrhosis**	
**ReMELD-Na**	0.760	0.682	0.078	0.548	0.576	-0.028
**MELD 3.0**	0.793	0.711	0.082	0.551	0.591	-0.04
**FIPS**	0.799	0.733	0.066	0.579	0.614	-0.035
**MELD**	0.765	0.707	0.058	0.549	0.572	-0.023
**MELD-Na**	0.788	0.697	0.091	0.561	0.599	-0.038

1b)						
**Entire Cohort**	**Female (n = 579)**	**Male (n = 1042)**	**Difference (ROC-AUC female-male)**	**Female (n = 579)**	**Male (n = 1042)**	**Difference (ROC-AUC female-male)**
**(90-day combined event (Death/LTx))**	**Age < 59 years**	**Age < 59 years**		**Age > 59 years**	**Age > 59 years**	
**ReMELD-Na**	0.674	0.655	0.019	0.687	0.572	0.115
**MELD 3.0**	0.691	0.667	0.024	0.707	0.598	0.109
**FIPS**	0.697	0.662	0.035	0.701	0.62	0.081
**MELD**	0.665	0.647	0.018	0.69	0.585	0.105
**MELD-Na**	0.703	0.675	0.028	0.703	0.592	0.111

1c)						
**Entire Cohort**	**Female (n = 579)**	**Male (n = 1042)**	**Difference (ROC-AUC female-male)**	**Female (n = 579)**	**Male (n = 1042)**	**Difference (ROC-AUC female-male)**
**(90-day combined event (Death/LTx))**	**Creatinine < 1.26 mg/dl**	**Creatinine < 1.26 mg/dl**		**Creatinine > 1.26 mg/dl**	**Creatinine > 1.26 mg/dl**	
**ReMELD-Na**	0.694	0.641	0.053	0.570	0.541	0.029
**MELD 3.0**	0.704	0.644	0.060	0.600	0.577	0.023
**FIPS**	0.695	0.646	0.049	0.641	0.614	0.027
**MELD**	0.690	0.605	0.085	0.574	0.576	-0.002
**MELD-Na**	0.706	0.640	0.066	0.612	0.581	0.031

1d)						
**Entire Cohort**	**Female (n = 579)**	**Male (n = 1042)**	**Difference (ROC-AUC female-male)**	**Female (n = 579)**	**Male (n = 1042)**	**Difference (ROC-AUC female-male)**
**(90-day combined event (Death/LTx))**	**Albumin < 29 g/l**	**Albumin < 29 g/l**		**Albumin > 29 g/l**	**Albumin > 29 g/l**	
**ReMELD-Na**	0.698	0.566	0.132	0.662	0.66	0.002
**MELD 3.0**	0.739	0.591	0.148	0.672	0.67	0.002
**FIPS**	0.719	0.621	0.098	0.712	0.687	0.025
**MELD**	0.714	0.592	0.122	0.653	0.635	0.018
**MELD-Na**	0.736	0.593	0.143	0.67	0.667	0.003

Table S1: Results of the ROC analysis with ROC-AUC values displayed for ReMELD-Na, MELD 3.0, FIPS, MELD and MELD-Na stratified by sex and a) no alcohol-related cirrhosis/alcohol-related cirrhosis, b) age below or above median of 59 years, c) creatinine below or above median of 1.26 mg/dl, d) albumin below or above median of

29 g/l. Furthermore, differences of ROC-AUC between the female and male subcohort are displayed. Outcome: 90-day combined event (Death/LTx).

**2a)**						
**Entire Cohort**	**Female (n = 579)**	**Male (n = 1042)**	**Difference (ROC-AUC female-male)**	**Female (n = 579)**	**Male (n = 1042)**	**Difference (ROC-AUC female-male)**
**(one-year combined event (Death/LTx))**	**No alcohol-related cirrhosis**	**No alcohol-related cirrhosis**		**Alcohol-related cirrhosis**	**Alcohol-related cirrhosis**	
**ReMELD-Na**	0.706	0.691	0.015	0.64	0.608	0.032
**MELD 3.0**	0.736	0.696	0.04	0.638	0.615	0.023
**FIPS**	0.714	0.721	-0.007	0.652	0.639	0.013
**MELD**	0.699	0.689	0.01	0.63	0.607	0.023
**MELD-Na**	0.73	0.683	0.047	0.653	0.614	0.039

**2b)**						
**Entire Cohort**	**Female (n = 579)**	**Male (n = 1042)**	**Difference (ROC-AUC female-male)**	**Female (n = 579)**	**Male (n = 1042)**	**Difference (ROC-AUC female-male)**
**(one-year combined event (Death/LTx))**	**Age < 59 years**	**Age < 59 years**		**Age > 59 years**	**Age > 59 years**	
**ReMELD-Na**	0.665	0.643	0.022	0.669	0.628	0.041
**MELD 3.0**	0.673	0.645	0.028	0.682	0.638	0.044
**FIPS**	0.68	0.666	0.014	0.663	0.65	0.013
**MELD**	0.657	0.646	0.011	0.658	0.624	0.034
**MELD-Na**	0.681	0.644	0.037	0.686	0.627	0.059

2c)						
**Entire Cohort**	**Female (n = 579)**	**Male (n = 1042)**	**Difference (ROC-AUC female-male)**	**Female (n = 579)**	**Male (n = 1042)**	**Difference (ROC-AUC female-male)**
**(one-year combined event (Death/LTx))**	**Creatinine < 1.26 mg/dl**	**Creatinine < 1.26 mg/dl**		**Creatinine > 1.26 mg/dl**	**Creatinine > 1.26 mg/dl**	
**ReMELD-Na**	0.679	0.665	0.014	0.556	0.561	-0.005
**MELD 3.0**	0.672	0.676	-0.004	0.582	0.570	0.012
**FIPS**	0.652	0.652	0.000	0.615	0.627	-0.012
**MELD**	0.66	0.634	0.026	0.549	0.584	-0.035
**MELD-Na**	0.683	0.658	0.025	0.579	0.570	0.009

2d)						
**Entire Cohort**	**Female (n = 579)**	**Male (n = 1042)**	**Difference (ROC-AUC female-male)**	**Female (n = 579)**	**Male (n = 1042)**	**Difference (ROC-AUC female-male)**
**(one-year combined event (Death/LTx))**	**Albumin < 29g/l**	**Albumin < 29g/l**		**Albumin > 29g/l**	**Albumin > 29g/l**	
**ReMELD-Na**	0.676	0.587	0.089	0.656	0.687	-0.031
**MELD 3.0**	0.699	0.600	0.099	0.659	0.676	-0.017
**FIPS**	0.680	0.630	0.050	0.681	0.707	-0.026
**MELD**	0.682	0.602	0.080	0.633	0.666	-0.033
**MELD-Na**	0.706	0.579	0.127	0.659	0.669	-0.01

Table S2: Results of the ROC analysis with ROC-AUC values displayed for ReMELD-Na, MELD 3.0, FIPS, MELD and MELD-Na stratified by sex and a) no alcohol-related cirrhosis/alcohol-related cirrhosis, b) age below or above median of 59 years, c) creatinine below or above median of 1.26 mg/dl, d) albumin below or above median of 29 g/l. Furthermore, differences of ROC-AUC between the female and male subcohort are displayed. Outcome: one-year combined event (Death/LTx).

TIPS 2004-2013	Overall (n = 585)		TIPS 2014-2024	Overall (n = 1036)	
**90-day combined event (Death/LTx)**	AUC	Range	**90-day (combined event Death/LTx)**	AUC	Range
**ReMELD-Na**	0.591	0.542-0.640	**ReMELD-Na**	0.678	0.627-0.730
**MELD 3.0**	0.616	0.566-0.666	**MELD 3.0**	0.693	0.644-0.742
**FIPS**	0.641	0.593-0.690	**FIPS**	0.700	0.652-0.749
**MELD**	0.588	0.538-0.639	**MELD**	0.693	0.640-0.745
**MELD-Na**	0.619	0.569-0.669	**MELD-Na**	0.686	0.636-0.737

Table S3: Results of ROC analyses stratifying the cohort into patients receiving TIPS from 2004 to 2013 and 2014 to 2024. ROC-AUC values displayed for ReMELD-Na, MELD 3.0, FIPS, MELD and MELD-Na. Outcome: 90-day combined event (Death/LTx).

TIPS 2004-2013	Overall (n = 585)		TIPS 2014-2024	Overall (n = 1036)	
**One-year combined event (Death/LTx)**	AUC	Range	**One-Year combined event (Death/LTx)**	AUC	Range
**ReMELD-Na**	0.630	0.585-0.676	**ReMELD-Na**	0.658	0.618-0.698
**MELD 3.0**	0.641	0.595-0.686	**MELD 3.0**	0.662	0.624-0.701
**FIPS**	0.665	0.621-0.709	**FIPS**	0.671	0.631-0.710
**MELD**	0.624	0.578-0.670	**MELD**	0.660	0.620-0.700
**MELD-Na**	0.643	0.598-0.689	**MELD-Na**	0.656	0.617-0.695

Table S4: Results of ROC analyses stratifying the cohort into patients receiving TIPS from 2004 to 2013 and 2014 to 2024. ROC-AUC values displayed for ReMELD-Na, MELD 3.0, FIPS, MELD and MELD-Na. Outcome: One-year combined event (Death/LTx).

Bare Metal Stents	Overall (n = 187)	Covered Stents	Overall (n = 1434)

90-day combined event (Death/LTx)	AUC	Range	90-day combined event (Death/LTx)	AUC	Range
**ReMELD-Na**	0.635	0.544-0.716	**ReMELD-Na**	0.641	0.602-0.679
**MELD 3.0**	0.666	0.586-0.746	**MELD 3.0**	0.658	0.619-0.697
**FIPS**	0.710	0.635-0.785	**FIPS**	0.663	0.625-0.701
**MELD**	0.627	0.545-0.709	**MELD**	0.646	0.605-0.687
**MELD-Na**	0.668	0.588-0.747	**MELD-Na**	0.656	0.617-0.695

Table S5: Results of ROC analyses stratifying the cohort into patients receiving bare-metal stents and those receiving covered stents. ROC–AUC values are presented for ReMELD-Na, MELD 3.0, FIPS, MELD, and MELD-Na. Outcome: 90-day combined event (Death/LTx).

Bare Metal Stents	Overall (n = 187)		Covered Stents	Overall (n = 1434)	
**One-year combined event (Death/LTx)**	AUC	Range	**One-year combined event (Death/LTx)**	AUC	Range
**ReMELD-Na**	0.665	0.588-0.743	**ReMELD-Na**	0.649	0.617-0.681
**MELD 3.0**	0.689	0.613-0.766	**MELD 3.0**	0.654	0.622-0.685
**FIPS**	0.698	0.623-0.773	**FIPS**	0.665	0.634-0.697
**MELD**	0.658	0.580-0.737	**MELD**	0.646	0.614-0.679
**MELD-Na**	0.693	0.617-0.769	**MELD-Na**	0.651	0.619-0.682

Table S6: Results of ROC analyses stratifying the cohort into patients receiving bare-metal stents and those receiving covered stents. ROC–AUC values are presented for ReMELD-Na, MELD 3.0, FIPS, MELD, and MELD-Na. Outcome: One-year combined event (Death/LTx).

Score	Time	HR	95% CI	P value
**ReMELD-Na**	90-day	1.73	1.30–2.30	<0.001*
**MELD 3.0**	90-day	1.91	1.46–2.50	<0.001*
**MELD**	90-day	2.13	1.64–2.77	<0.001*
**MELD-Na**	90-day	2.00	1.53–2.63	<0.001*
**FIPS**	90-day	2.33	1.77–3.06	<0.001*
**ReMELD-Na**	one-year	1.70	1.39–2.08	<0.001*
**MELD 3.0**	one-year	2.07	1.70–2.53	<0.001*
**MELD**	one-year	1.97	1.61–2.40	<0.001*
**MELD-Na**	one-year	2.05	1.68–2.51	<0.001*
**FIPS**	one-year	2.24	1.83–2.73	<0.001*

Table S7: Results of the fine-gray proportional hazard analysis for each score in the entire study Cohort based on the division of the overall cohort at the 85th (90-day) and 75th (one-year) percentile of each score. Cutoff values used to define high-risk groups for the 90-day endpoint were: ReMELD-Na = 20, MELD 3.0 = 23, FIPS = 0.92, MELD = 19, and MELD-Na = 23. Cutoff values used to define high-risk groups for the one-year endpoint were: ReMELD-Na = 18, MELD 3.0 = 21, FIPS = 0.62, MELD = 17, and MELD-Na = 21. Outcome: 90-day and one-year cumulative incidence of death with the competing risk of LTx. Level of significance for each analysis: *p < 0.001 (Fine-Gray proportional hazard analysis).

90-day combined event (death/LTx))	Overall (n = 1621)		Female (n = 579)		Male (n = 1042)	
	PPV	NPV	PPV	NPV	PPV	NPV
**ReMELDNa**	28.3%	84.4%	37.8%	84.8%	24.4%	84.2%
**MELD 3.0**	29.8%	85.1%	37.6%	86.0%	25.5%	84.5%
**FIPS**	33.1%	85.2%	39.0%	85.1%	30.4%	85.2%
**MELD**	32.0%	85.6%	41.2%	85.8%	28.2%	85.4%
**MELDNa**	30.5%	84.9%	39.7%	85.2%	26.6%	84.8%

Table S8: Positive and negative predictive values (PPV and NPV) for each score in the entire study Cohort based on the division of the overall, female and male training cohort at the 85th percentile of each score. Outcome: 90-day combined event (Death/LTx).

One-year combined event (death/LTx))	Overall (n = 1621)		Female (n = 579)		Male (n = 1042)	
	PPV	NPV	PPV	NPV	PPV	NPV
**ReMELDNa**	39.9%	76.0%	42.9%	77.9%	38.7%	74.8%
**MELD 3.0**	42.8%	77.2%	45.6%	79.9%	41.3%	75.6%
**FIPS**	43.6%	77.0%	45.0%	78.7%	42.9%	76.1%
**MELD**	42.2%	77.1%	42.9%	78.3%	41.9%	76.4%
**MELDNa**	42.7%	76.9%	45.6%	78.6%	41.5%	75.8%

Table S9: Positive and negative predictive values (PPV and NPV) for each score in the entire study Cohort based on the division of the overall, female and male training cohort at the 85th percentile of each score. Outcome: one-year combined event (Death/LTx).

Supplementary references

Author names in bold designate shared co-first authorship.

1. Bloom S, Kemp W, Lubel J. Portal hypertension: pathophysiology, diagnosis and management. Intern. Med. J. 2015;45:16–26.
2. Iwakiri Y, Trebicka J. Portal hypertension in cirrhosis: Pathophysiological mechanisms and therapy. JHEP Rep. Innov. Hepatol. 2021;3:100316.
3. Gu W, Hortlik H, Erasmus H-P, *et al.* Trends and the course of liver cirrhosis and its complications in Germany: Nationwide population-based study (2005 to 2018). Lancet Reg. Health Eur. 2022;12:100240.
4. Volk ML, Tocco RS, Bazick J, *et al.* Hospital Readmissions Among Patients With Decompensated Cirrhosis. Off. J. Am. Coll. Gastroenterol. ACG 2012;107:247.
5. Allaire M, Walter A, Sutter O, *et al.* TIPS for management of portal-hypertension-related complications in patients with cirrhosis. Clin. Res. Hepatol. Gastroenterol. 2020;44:249–263.
6. Larrue H, D'Amico G, Olivas P, *et al.* TIPS prevents further decompensation and improves survival in patients with cirrhosis and portal hypertension in an individual patient data meta-analysis. J. Hepatol. 2023;79:692–703.
7. Trebicka J. Emergency TIPS in a Child-Pugh B patient: When does the window of opportunity open and close? J. Hepatol. 2017;66:442–450.
8. Gu W, Zeleke Y, Hortlik H, *et al.* Use and outcome of TIPS in hospitalized patients in Germany: A Nationwide study (2007-2018). Hepatol. Commun. 2023;7:e0237.
9. Ruf AE, Kremers WK, Chavez LL, *et al.* Addition of serum sodium into the MELD score predicts waiting list mortality better than MELD alone. Liver Transpl. 2005;11:336.
10. Biggins SW, Kim WR, Terrault NA, *et al.* Evidence-based incorporation of serum sodium concentration into MELD. Gastroenterology 2006;130:1652–1660.
11. **Nagai S, Chau LC,** Schilke RE, *et al.* Effects of Allocating Livers for Transplantation Based on Model for End-Stage Liver Disease–Sodium Scores on Patient Outcomes. Gastroenterology 2018;155:1451-1462.e3.

12. Kim WR, Mannalithara A, Heimbach JK, *et al.* MELD 3.0: The Model for End-Stage Liver Disease Updated for the Modern Era. Gastroenterology 2021;161:1887-1895.e4.

13. Ärzteblatt DÄG Redaktion Deutsches. Richtlinie gem. § 16 Abs. 1 S. 1 Nrn. 2 und 5 TPG für die Wartelistenführung und Organvermittlung zur Lebertransplantation Richtlinie gemäß § 16 Abs. 1 S. 1 Nrn. 2 u. 5 TPG für die Wartelistenführung und Organvermittlung zur Lebertransplantation (RL BÄK Leber). Dtsch. Ärztebl. 2025.

14. Rössle M. Liver: Early TIPS in patients with cirrhosis and variceal bleeding. Nat. Rev. Gastroenterol. Hepatol. 2010;7:536–538.

15. Franchis R de. Evolving Consensus in Portal Hypertension Report of the Baveno IV Consensus Workshop on methodology of diagnosis and therapy in portal hypertension. J. Hepatol. 2005;43:167–176.

16. Franchis R de. Revising consensus in portal hypertension: Report of the Baveno V consensus workshop on methodology of diagnosis and therapy in portal hypertension. J. Hepatol. 2010;53:762–768.

17. Franchis R de. Expanding consensus in portal hypertension: Report of the Baveno VI Consensus Workshop: Stratifying risk and individualizing care for portal hypertension. J. Hepatol. 2015;63:743–752.

18. Franchis R de, Bosch J, Garcia-Tsao G, *et al.* Baveno VII – Renewing consensus in portal hypertension. J. Hepatol. 2022;76:959–974.

19. Gerbes AL, Gülberg V, Sauerbruch T, *et al.* S3-Leitlinie „Aszites, spontan bakterielle Peritonitis, hepatorenales Syndrom". Z. Für Gastroenterol. 2011;49:749–779.

20. Gerbes AL, Labenz J, Appenrodt B, *et al.* [Updated S2k-Guideline "Complications of liver cirrhosis". German Society of Gastroenterology (DGVS)]. Z. Gastroenterol. 2019;57:e168.

21. Kamath PS, Kim WR, Advanced Liver Disease Study Group. The model for end-stage liver disease (MELD). Hepatol. Baltim. Md 2007;45:797–805.

22. Bettinger D, Sturm L, Pfaff L, *et al.* Refining prediction of survival after TIPS with the novel Freiburg index of post-TIPS survival. J. Hepatol. 2021;74:1362–1372.

23. Song J, Wang X, Yan Y, *et al.* MELD 3.0 Score for Predicting Survival in Patients with Cirrhosis After Transjugular Intrahepatic Portosystemic Shunt Creation. Dig. Dis. Sci. 2023;68:3185–3192.

24. Song J, Wang X, Yan Y, *et al.* MELD 3.0 Score for Predicting Survival in Patients with Cirrhosis After Transjugular Intrahepatic Portosystemic Shunt Creation. Dig. Dis. Sci. 2023;68:3185–3192.

25. Wiering L, Aigner A, Rosmalen M van, *et al.* Systematic Sex-Based Inequity in the MELD Score-Based Allocation System for Liver Transplantation in Germany. Transpl. Int. 2025;38:13844.

26. Fozouni L, Wang CW, Lai JC. Sex Differences in the Association Between Frailty and Sarcopenia in Patients With Cirrhosis. Clin. Transl. Gastroenterol. 2019;10:e00102.

27. Gödiker J, Schwind L, Jacob T, *et al.* Ultrasound-Defined Sarcopenia Independently Predicts Acute Decompensation in Advanced Chronic Liver Disease. J. Cachexia Sarcopenia Muscle 2024;15:2792–2802.

28. Delgado MG, Mertineit N, Bosch J, *et al.* Combination of Model for End-Stage Liver Disease (MELD) and Sarcopenia predicts mortality after transjugular intrahepatic portosystemic shunt (TIPS). Dig. Liver Dis. Off. J. Ital. Soc. Gastroenterol. Ital. Assoc. Study Liver 2024;56:1544–1550.

29. Schattenberg JM, Chalasani N, Alkhouri N. Artificial Intelligence Applications in Hepatology. Clin. Gastroenterol. Hepatol. 2023;21:2015–2025.
